# Overcoming Barriers in Solid Tumors: Lactate-Fueled Nanomotors Enhance Penetration and Reverse Immunosuppression

**DOI:** 10.1021/jacs.6c05546

**Published:** 2026-08-11

**Authors:** Juan José Esteve-Moreno, Andrea Escudero, Francisco J. Hicke, Víctor Sanz-Álvarez, María Miralles-Buleo, María Dolores Marcos, M. Carmen Martínez-Bisbal, Alba García-Fernández, Paula Díez, Ramón Martínez-Máñez

**Affiliations:** † Instituto Interuniversitario de Investigación de Reconocimiento Molecular y Desarrollo Tecnológico (IDM), 16774 Universitat Politècnica de València, Universitat de València, Camí de Vera, s/n 46022 València, Spain; ‡ Unidad Mixta UPV-CIPF de Investigación en Mecanismos de Enfermedades y Nanomedicina, Centro de Investigación Príncipe Felipe, Universitat Politècnica de València, C/Eduardo Primo Yúfera, 3, 46012 València, Spain; § CIBER de Bioingeniería, Biomateriales y Nanomedicina (CIBER-BBN), Instituto de Salud Carlos III, 28029 Madrid, Spain; ∥ Unidad Mixta de Investigación en Nanomedicina y Sensores, Universitat Politècnica de València, Instituto de Investigación Sanitaria La Fe (IIS La Fe), Avenida Fernando Abril Martorell 106, Torre A, 46026 València, Spain; ⊥ Departamento de Química, Universitat Politècnica de València, Camí de Vera s/n 46022 València, Spain; # Departamento de Química-Física, Universitat de València, Burjassot, 46100, València,Spain

## Abstract

Solid tumors present a therapeutic challenge due to their dense extracellular matrix and metabolically immunosuppressive microenvironment, which limit drug penetration and immune clearance. To overcome these barriers, we describe an engineered lactate-powered Janus nanomotor (NM­(dox)-LOx) composed of a platinum (Pt) nanodendrite and a mesoporous silica nanoparticle (MSN), loaded with doxorubicin (Dox) and functionalized with the enzyme lactate oxidase (LOx). In this design, LOx acts as a protease-sensitive molecular gate for controlled Dox release and catalyzes the oxidation of tumor-accumulated lactate, using this metabolite as a biofuel to generate hydrogen peroxide, which is catalyzed by Pt into oxygen and water. This cascade generates a self-propulsive force that drives the nanomotor. Concurrently, catalytic lactate depletion reprograms the tumor microenvironment, promoting cytotoxic T cell infiltration and restoring immune activity. NM­(dox)-LOx demonstrates efficient tumor accumulation and potent antitumor activity in human SK-MEL-103 and murine B16–F10 melanoma models, including 2D cultures, 3D spheroids, and *in vivo* tumors. Remarkably, treatment in an *in vivo* B16–F10 melanoma model achieved pronounced tumor regression and enhanced cytotoxic T cell infiltration, while maintaining minimal systemic toxicity. Overall, this multifunctional nanomotor couples enhanced drug delivery with immune activation, effectively overcoming the structural and metabolic barriers of solid tumors, and represents a promising strategy for self-propelled therapeutic nanoparticles in cancer treatment.

## Graphical abstract

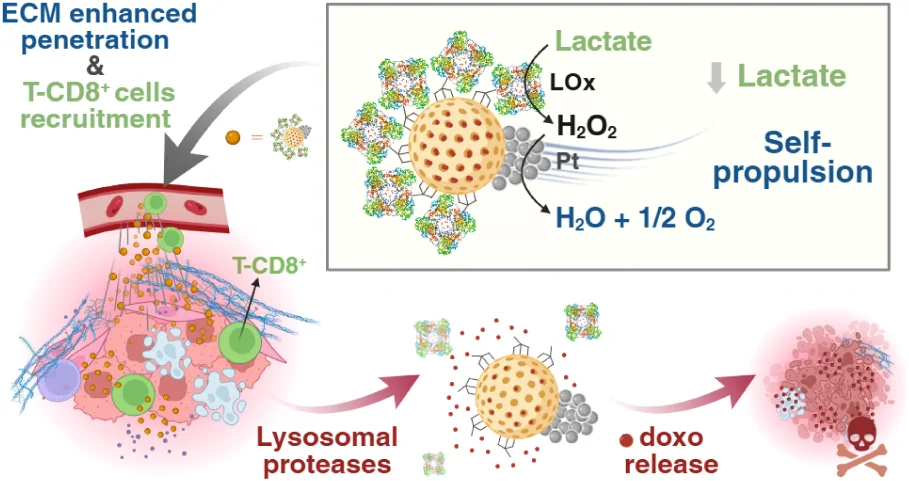

## 1 Introduction

Cancer remains one of the leading causes of death worldwide, responsible for one in five deaths (WHO, 2022). Treating solid tumors is particularly challenging due to their complex microenvironment. Together, the metabolic and structural features of solid tumors create a dual obstacle, with conventional therapies failing to reach deep tumor regions, and the immunosuppressive tumor microenvironment (TME) preventing effective immune clearance.^1^ Among the defining metabolic features of the TME is the accumulation of lactate. Elevated lactate levels correlate with poor prognosis in highly glycolytic cancers such as lung and breast cancer, melanoma, or glioblastoma.^2,3,4^ Lactate production acidifies TME, disrupting intracellular signaling and metabolism, and impair T and natural killer (NK) cell activity while promoting immunosuppressive macrophage phenotypes.^2,5^ In addition, the extracellular matrix (ECM) of solid tumors constitutes a dense physical barrier that not only contributes to cancer progression and immune evasion but also hinders the effective penetration and distribution of therapeutic agents within the tumor tissue. Therefore, the ECM ultimately limits the drugs’ access to cancer cells in deep tumor areas, thereby reducing their therapeutic efficacy.^6^ Consequently, novel onco-therapeutic approaches, including innovative drug combinations and nanotechnology-based strategies, are under active investigation to overcome ECM-mediated cancer resistance and immunosuppressive TME.^7^

Among nanotechnology-based strategies to enhance penetration into the ECM, nanomotors (*i.e*. nanodevices endowed with autonomous motion) have emerged as particularly promising tools for precision medicine applications. Unlike passive nanocarriers, nanomotors generate movement that enables on-demand actuation and navigation through biological barriers, including the ECM,^8^ mucosal membranes,^9^ and the blood–brain barrier,^10^ while providing localized drug release, preserving the protective and selective properties of conventional nanocarriers.^11^ Nanomotors’ movement can be controlled by external sources, such as magnetic fields,^12^ ultrasound,^13^ heat or light,^14^ however nanomotors fueled by endogenous fuels are especially appealing for biomedical applications.^15,16^ Thus, while early designs, exemplified by Pt-powered nanomotors,^17^ prioritized propulsion efficiency using reactive chemical fuels like H_2_O_2_,^18^ their application was often limited by poor biocompatibility of H_2_O_2_ under physiological conditions. In contrast, enzyme-based motors employing redox enzymes, such as oxidases or catalases, offered greater biocompatibility.^19,20,21^ However, their efficiency at physiological substrate concentrations is frequently insufficient, and their integration into functional drug delivery systems remains a significant challenge.^22,23^ This motivated the development of hybrid metal-enzyme nanomotors (e.g., Pt-oxidase platforms), which combine the catalytic robustness of metals with the substrate specificity of enzymes.^16,24,25^ As biocompatible fuels, recent designs take advantage of endogenous metabolites such as glucose, urea, amino acids, ions, and tumor-associated substrates. Some of these metabolite-driven nanomotors integrate propulsion, targeting, and controllable drug release within a single platform, enabling more effective *in vivo* applications. Among endogenous biocompatible fuels explored to date, tumor-enriched metabolites such as lactate offer promising opportunities for more selective and effective therapies. In this scenario, several recent studies have validated the use of lactate as fuel source, including Pt nanoflowers functionalized lactate oxidase (LOx), which exhibited lactate-driven chemotaxis and improved penetration into solid tumors^26^ as well as lactate-powered nanoparticles that enhanced diabetic wound healing.^27^

In the context of leveraging nanomotors to enhance antitumor immunity and overcome immunosuppressive TME, this field is rapidly emerging as a highly relevant frontier in cancer nanomedicine.^28^ Most recent reported nanomotor platforms for this application depend on external activation, commonly near-infrared (NIR) irradiation, to induce immunogenic effects or to improve the intratumoral delivery of immunotherapeutic agents.^29,30^ These externally triggered systems typically rely on photothermal or photodynamic mechanisms to modulate the tumor microenvironment, stimulate immunogenic cell death, or facilitate immune-cell infiltration. However, their dependence on light penetration and localized irradiation limits their applicability in deep or heterogeneous tumors, underscoring the need for metabolically driven, self-sustained propulsion strategies capable of operating autonomously within the immunosuppressive tumor environment.

Although these pioneering studies demonstrate the potential of nanomotors to either potentiate antitumor immune responses or facilitate extracellular matrix penetration, the rational design of nanomotor platforms capable of concurrently addressing these principal barriers of solid tumors, namely the biomechanical constraints imposed by the dense ECM and the immunosuppressive TME, while also integrating spatiotemporally controlled drug-release functionalities remains largely unexplored.

Based on the above, we report herein the development of a lactate-propelled Janus Pt–mesoporous silica (Pt-MSN) nanomotor in which the MSN face is loaded with doxorubicin (Dox) and its external surface functionalized with LOx (Scheme 1). LOx performs two coordinated functions, it acts as a protease-responsive molecular gate that enables on-demand, lysosome-triggered intracellular release of Dox, and it catalyzes the oxidation of tumor-enriched lactate into H_2_O_2_. The generated H_2_O_2_ is subsequently decomposed on the Pt face into O_2_ and H_2_O, establishing a sequential catalytic cascade that induces self-diffusiophoretic propulsion. NM­(dox)-LOx exhibit multiple functional capabilities: (i) enhanced propulsion driven by the endogenous fuel lactate, (ii) lactate consumption, thereby depleting a key immunosuppressive metabolite and promoting an immunostimulatory microenvironment, (iii) controlled selective intracellular release of Dox, (iv) generation of reactive oxygen species (ROS), and (v) contribution to hypoxia alleviation in tumor microenvironment. NM­(dox)-LOx is validated across multiple melanoma platforms, including human SK-MEL-103 and murine B16–F10 models in 2D culture, 3D spheroids, and *in vivo*. Melanoma was selected due to its aggressive growth, high glycolytic activity, and ECM-rich microenvironment, providing an ideal setting to evaluate strategies that simultaneously enhance drug delivery and modulate tumor metabolism. Together, this lactate-powered nanomotor represents a multifunctional therapeutic platform capable of overcoming both the physical and metabolic barriers of solid tumors, enabling deeper tissue penetration, more effective drug delivery, and enhanced immune system engagement.

**Scheme 1:**
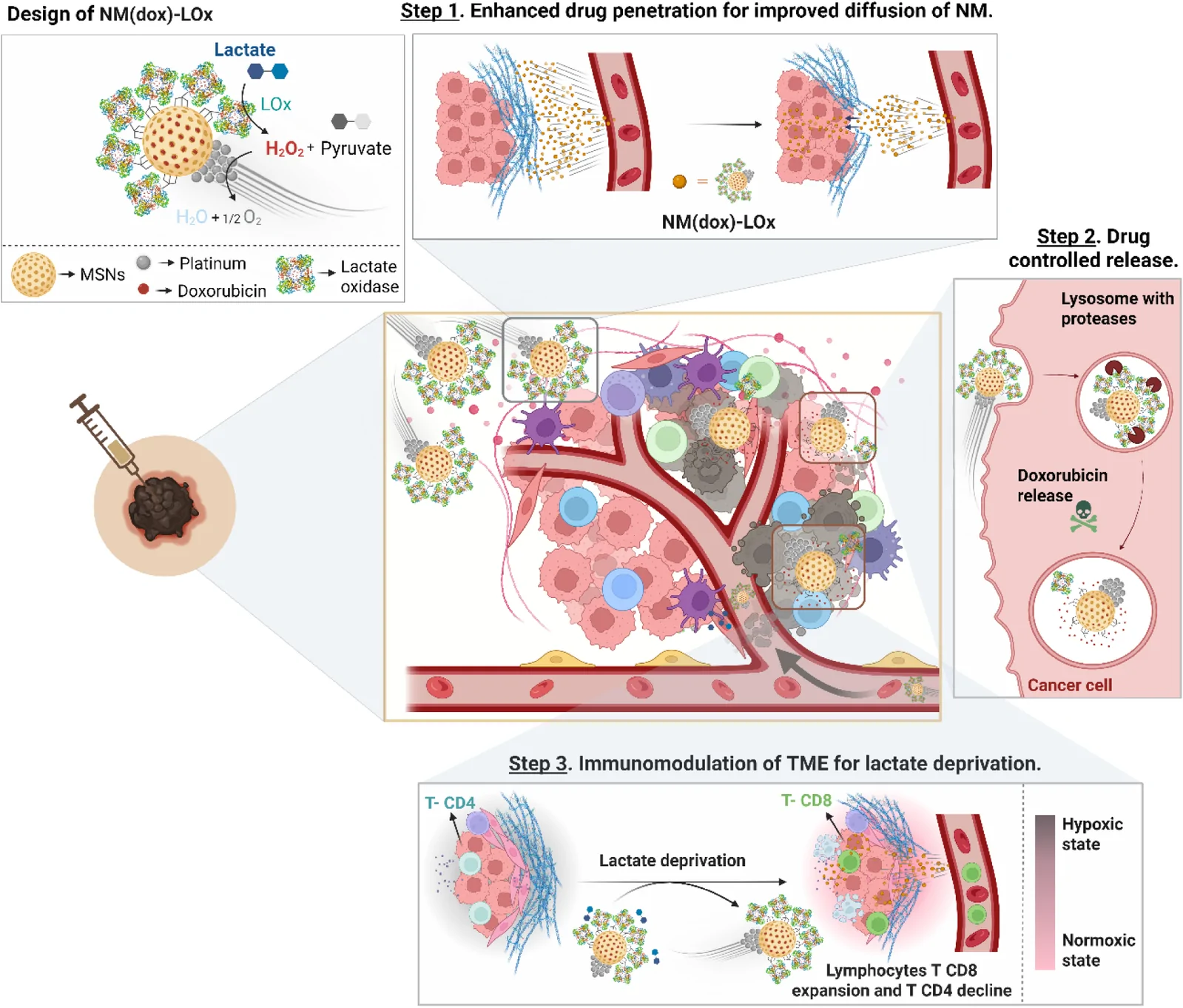
Schematic Representation of the Mechanism of Action of **NM­(dox)-LOx** Nanomotor for Cancer Therapy^a^

a NM­(dox)-LOx consists of a Janus Pt-MSN nanoparticle loaded with doxorubicin and functionalized with lactate oxidase (LOx). LOx catalyzes the oxidation of abundant lactate in the TME, generating hydrogen peroxide, which is subsequently catalyzed on the Pt surface to produce water and oxygen. The generation of these products drives the self-propulsion of the nanomotor (Step 1). In the second step, lysosomal proteases hydrolyze the LOx gatekeeper, thereby enabling doxorubicin release and inducing a direct cytotoxic effect in cancer cells (Step 2). Additionally, lactate deprivation contributes to immunomodulation of the tumor microenvironment, promoting the recruitment and activation of cytotoxic CD8+ T cells, ultimately enhancing the overall antitumor cytotoxic response, and could help to alleviate hypoxia by utilizing endogenous H_2_O_2_ (Step 3).

## 2 Results and Discussion

### 2.1 Design, Synthesis, and Characterization of Nanomotors

To synthesize the NM­(dox)-LOx nanomotor, mesoporous silica nanoparticles (MSNs) were combined with platinum nanoparticles (PtNPs) to obtain Janus Pt-MSN nanoparticles (NMs) as the inorganic framework. Briefly, NMs were synthesized using a Pickering emulsion, in which MSNs were located at the oil–water interface. The exposed face of the MSN was sequentially modified with 3-(mercaptopropyl)­trimethoxysilane and reacted with PtNPs, resulting in NMs. Subsequently, the MSN face in NMs was functionalized with 3-(aminopropyl)­triethoxysilane (APTES) and loaded with Dox in the pores. Finally, carboxylic groups in LOx enzyme were activated with EDC/NHS, enabling amide bond formation with the amine groups of APTES, resulting in the formation of NM­(dox)-LOx.^31^ A scheme of the synthetic procedure to obtain NM­(dox)-LOx is shown in Figure 1A.

**Figure 1:**
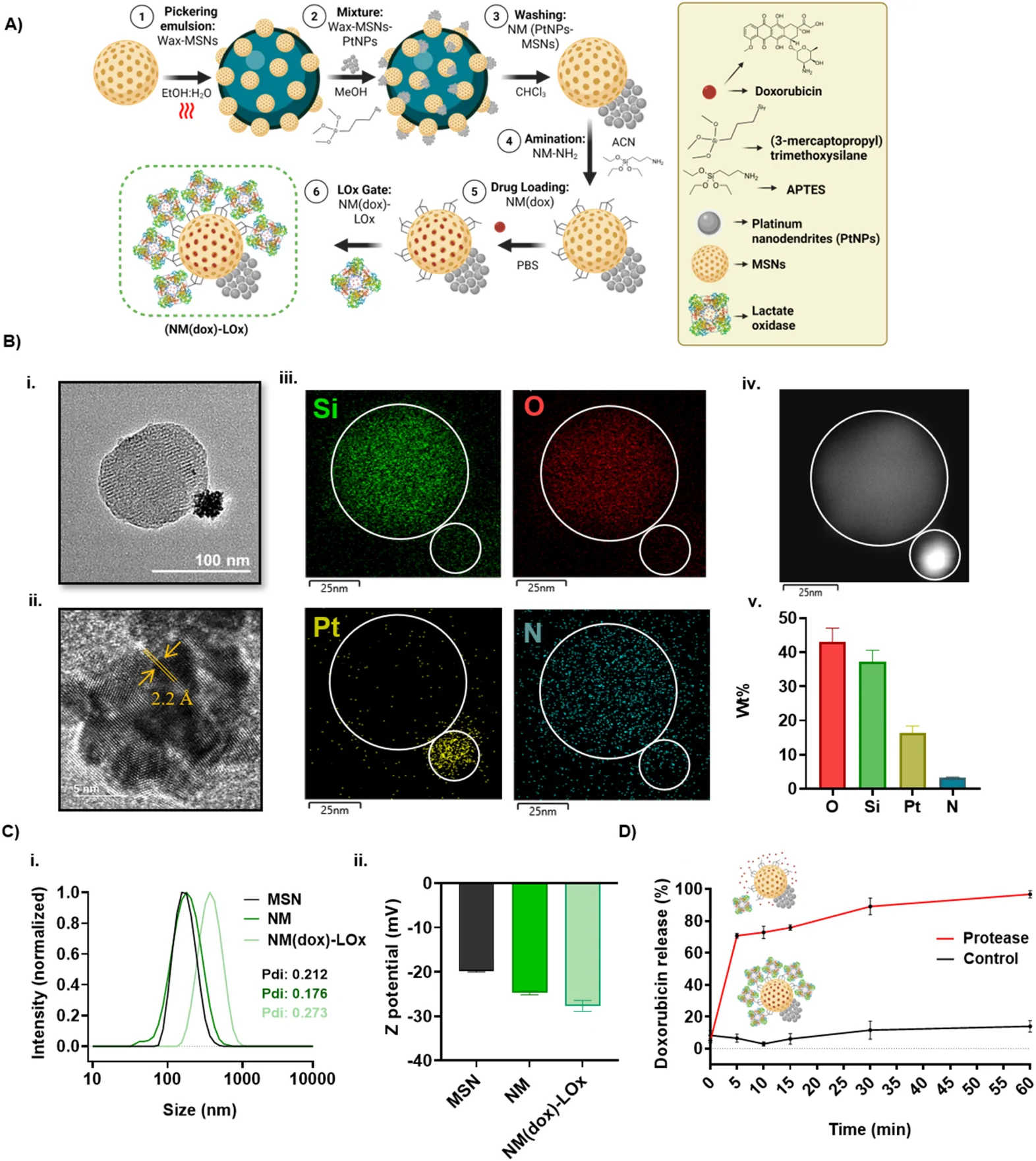
Structural characterization and controlled-release studies. A) Schematic representation of the synthetic procedure to obtain NM­(dox)-LOx. B-i) HR-TEM image of NM showing the hexagonal pore structure of MSNs and successful anisotropic conjugation with PtNPs (scale bar: 100 nm). B-ii) HR-TEM image of the Pt face of NM displaying crystalline (111) planes with an interplanar spacing of 2.2 Å (scale bar: 5 nm). B-iii to B-v) STEM-EDX elemental mapping of NM-LOx, confirming the presence of O (red), Si (green), Pt (yellow) and N (blue) atoms and the quantification of each one. C) Changes in hydrodynamic diameter (i) and z-potential (ii) during NM­(dox)-LOx synthesis (black: MSN; dark green: NM; light green: NM­(dox)-LOx), determined by DLS. D) Normalized Dox release profile from NM­(dox)-LOx monitored by fluorescence spectroscopy (λ_em_ = 555 nm; λ_ex_ = 470 nm) in the presence (red) and absence (black) of *Streptomyces griseus* proteases (1 mg mL^–1^) added at 0 min. Data represent mean ± SEM (n = 3).

We also prepared control nanoparticles in which specific functional components of the nanomotor were removed, thereby intentionally impairing its propulsion or delivery capabilities. Thus, we synthesized nanoparticles lacking the therapeutic drug Dox (NM-LOx) and nanoparticles unable to act as nanomotors due to thermal denaturation of the LOx enzyme (NM­(dox)-LOx-i). An additional control containing all disassembled elements in NM­(dox)-LOx (i.e., MCM, Dox, PtNPs, and LOx) at equivalent concentrations (DA) was also used. Moreover, the corresponding nanomotor and the passive control were covalently labeled with a fluorophore (nanoparticles named NM*-LOx and NM*-LOx-i, respectively) for confocal laser scanning microscopy (CLSM) studies. An overview of all the prepared nanomaterials is shown in Table S1.

The successful synthesis of the various nanoparticles was verified through standard characterization techniques. As demonstrated by high-resolution transmission electron microscopy (TEM), NMs showed a characteristic “snowman”-like morphology with two distinct faces of MSN and PtNPs (Figure 1B-i). Moreover, HR-TEM revealed that the Pt face exhibits lattice fringes with an interplanar spacing of 2.2 Å, corresponding to the (111) plane of a face-centered cubic structure (Figure 1B-ii). Powder X-ray diffraction (PXRD) also confirmed the crystalline structure of NMs (Figure S1). Bragg peaks at high angles, characteristic of PtNPs, indexed as planes (111), (200), and (220), were found. Moreover, the low-angle spectrum revealed the typical diffraction peaks of MCM-41 materials; i.e., a main peak at 2.4° (indexed as plane (100)) and two minor peaks at 4.2 and 4.8° (corresponding to planes (110) and (200)). These PXRD patterns were consistent with the reference diffraction peaks of both Pt (JCPDS/ICDD standard) and the ordered mesoporous structure of MCM-41. Moreover, they remained unaltered in the final nanomotor, demonstrating that the processes of loading and functionalization did not alter the NM structure. Scanning transmission electron microscopy coupled with energy-dispersive X-ray spectroscopy (STEM-EDX) revealed the expected atomic composition (wt %) from each face of NM­(dox)-LOx (Figure 1B-iii to 1B-v). Oxygen and silicon were the predominant elements (42.8% and 37.4%, respectively), corresponding to the MSN face, while platinum was localized on the opposite face, representing 16.4% of the total composition. Nitrogen atoms, attributed to the immobilized enzyme in the MSN face, and doxorubicin in the pores were also detected (3.4%).

The N_2_ adsorption–desorption isotherms corroborated the formation of the mesoporous network in MSN, its preservation in NM and the effective pore blocking achieved after LOx functionalization, which resulted in a significant decrease in N_2_ uptake (Figure S2). Application of the Barrett–Joyner–Halenda (BJH) model yielded pore diameter and specific volume values of 2.69 nm and 0.98 cm^3^ g^–1^ for MSN, 2.49 nm and 0.32 cm^3^ g^–1^ for NM, and nearly zero and 0.064 cm^3^ g^–1^ for NM-LOx. Similarly, Brunauer–Emmett–Teller (BET) analysis revealed total surface areas of 1329 m^2^ g^–1^ for MSN, 663.4 m^2^ g^–1^ for NM, and 70.1 m^2^ g^–1^ for NM-LOx (Table S2). LOx functionalization in NM-LOx was confirmed by FTIR with the appearance of characteristic protein absorption bands corresponding to the amide I bond (1650 cm^–1^), C–N stretching (1541 cm^–1^), and CH_2_ groups (2915 cm^–1^) (Figure S3). Moreover, LOx content was quantified by bicinchoninic acid assay (BCA), resulting in 314 and 356 μg per mg of NM-LOx and NM­(dox)-LOx, respectively (Table S3).

Each step in the construction of NM­(dox)-LOx was also studied by dynamic light scattering (DLS) and z-potential. DLS showed increasing values in the size starting from 133 ± 25 nm for MSN, reaching 170 ± 1 nm for NM, and finally obtaining a value of 409.7 ± 17.3 nm for NM­(dox)-LOx (Figure 1C-i). For z-potential values, MSN presented a negative charge of −19.8 ± 0.4 mV, which shifted to −24.7 ± 0.7 for NM, to 7.3 ± 0.3 mV when functionalized with APTES and finally to −27.2 ± 2.8 mV for NM­(dox)-LOx due to the presence of LOx, which presents a high content of acidic residues (Asp and Glu), resulting in a net negative charge at physiological pH (pI = 4.3)^24^ (Figure 1C-ii). The size and z-potential of control nanoparticles were also evaluated, obtaining comparable results (Figure S4).

Finally, we assessed the reproducibility of NM­(dox)-LOx synthesis by analyzing three independently prepared batches (S1–S3) generated from separate Pickering emulsions using multiple characterization techniques. The results showed a high degree of consistency in the size and MSN:Pt ratio of the nanoparticles, as well as in the surface charge and hydrodynamic diameter of the final nanomotors. These results confirm that the Pickering emulsion approach combined with EDC/NHS chemistry for enzyme conjugation is a robust and reliable method for the preparation of biomedical nanodevices (Figure S5 and Table S4).

### 2.2 NM­(dox)-LOx Selectively Delivers Doxorubicin in the Presence of Proteases

Next, we assessed the nanomotor ability to control drug release under specific conditions. NM­(dox)-LOx is designed to show “zero” release, yet to deliver Dox in the lysosomes of cancer cells due to the high activity of lysosomal proteases that are expected to hydrolyze the LOx gatekeeper.^32^ We evaluated Dox release from NM­(dox)-LOx in the presence of *Streptomyces griseus* proteases and purified lysosomal extract to reproduce the intracellular environment. Dox release from NM­(dox)-LOx (measured by fluorescence spectroscopy) occurred in the presence of proteases (Figure 1D) that induced a marked increase in drug release, reaching a maximum after approximately 60 min (red curve), consistent with LOx degradation. In contrast, in the absence of proteases, the nanomotor retained the drug within its pores (black curve). Dox released was quantified as 72.3 μg per mg of NM­(dox)-LOx (Table S5). Moreover, forced-release experiments performed on three independent batches of NM­(dox)-LOx using DMSO for 24 h determined a drug content of 105.6 ± 12.5 μg per mg of nanoparticles (Tables S4 and S5). This indicates that 68% of the loaded drug is released in the presence of proteases, while also confirming good batch-to-batch reproducibility of the drug content. Controlled doxorubicin release assays using purified lysosomal were also carried out (Figure S6). As observed, the drug is preferably released in the presence of lysosomal extract compared with the control. Overall, these results demonstrated that the nanomotor effectively regulates payload delivery in the presence of proteases, achieving controlled release of doxorubicin. However, it should be noted that, although the LOx gatekeeper is conceived to be cleaved predominantly by lysosomal proteases after cellular internalization, the extracellular tumor microenvironment is also enriched in matrix metalloproteinases and other secreted proteases, which could promote partial extracellular degradation of the gatekeeper, potentially leading to partial extracellular drug release. However, the active motion of the nanomotors is expected to favor rapid tumor penetration and cellular internalization (*vide infra*).

### 2.3 Functions of the Nanomotor in Response to Lactate: Substrate Depletion and Propulsion

Nanomotor propulsion is expected to be achieved through the enzymatic conversion of lactate (overexpressed in the tumor microenvironment)^33^ into H_2_O_2_ by LOx (Lactate + O_2_ → Pyruvate + H_2_O_2_), followed by decomposition of the *in situ* produced H_2_O_2_ on the Pt face (H_2_O_2_ → H_2_O + 1/2 O_2_) (Figure 2A). Given the essential role of this catalytic mechanism in the motility of nanomotors, several studies were conducted.

**Figure 2:**
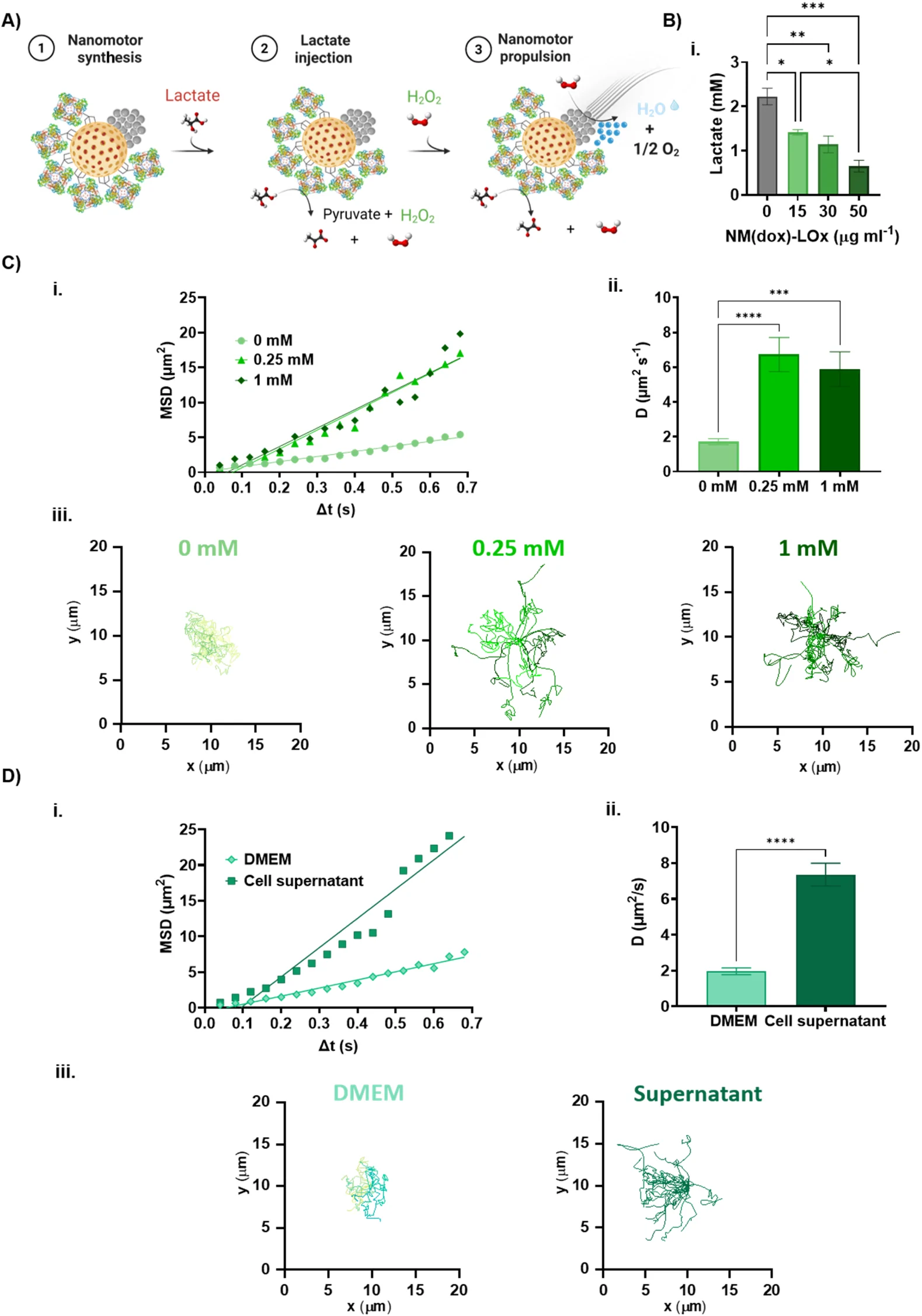
Analysis of nanomotor functionalities in response to lactate: lactate depletion and propulsion. A) Schematic illustration of cascade reactions in NM­(dox)-LOx leading to its motion. B) Characterization of lactate consumption by the nanomotor at concentrations ranging from 0 to 50 μg mL^–1^. C–D) Evaluation of NM­(dox)-LOx motion by NTA. C-i) MSD vs Δt at different lactate concentrations (0 mM, light blue; 0.25 mM, blue; 1 mM, dark blue), C-ii) D_0_ and D_eff_ values calculated from MSD plots and C-iii) representative spatial trajectories. D-i) MSD vs Δt with DMEM (light blue) or with SK-MEL-103 cell supernatant (dark blue), D-ii) D_0_ and D_eff_ values calculated from MSD plots and D-iii) representative spatial trajectories. Data are expressed as mean ± SEM for 25 nanodevices with sizes from 250 to 450 nm. Statistical significance was determined using one-way ANOVA (*p < 0.1, **p < 0.01, ***p < 0.001, ****p < 0.0001).

First, lactate production by different cells (WI-38, A549 SK-MEL-103) was evaluated at different time points (24 and 72 h, Figure S7 and Table S6), using the L-Lactate Assay Kit. Results indicated that melanoma SK-MEL-103 cells produced higher lactate levels than the other cell types, with a time-dependent accumulation of the metabolite in the culture supernatant. SK-MEL-103 cells produced 0.25 mM lactate at 24 h, increasing up to 2.2 mM at 72 h.^34,35^

Additionally, the lactate consumption driven by the catalytic activity of LOx was assessed. As shown in Figure 2B, the concentration of lactate in the supernatant of SK-MEL-103 cells (cultured for 72 h) decreased after 1 h incubation with increasing nanomotor concentrations. The nanomotor decreases lactate levels by roughly 50% at 30 μg mL^–1^ and up to 70% at 50 μg mL^–1^. These findings demonstrate that the nanomotor efficiently catalyzes the depletion of lactate produced by cancer cells.

To support this statement, the catalytic activity of LOx immobilized on three different batches of NM­(dox)-LOx was determined using a spectrophotometric assay (Tables S4 and S7). The immobilized enzyme exhibited an activity of 33.7 ± 2.5 U g^–1^ of NM­(dox)-LOx. Similar values were obtained for NM-LOx and NM*-LOx. In addition to LOx activity, the H_2_O_2_-reducing capability of the nanomotors Pt face was demonstrated by ultraviolet–visible (UV–vis) spectrophotometry. The catalytic constants for both (Pt and LOx in NM), including apparent affinity constant (K_M_), maximum velocity (*V*_max_), and turnover rate (*k*_cat_) were calculated (Table S8). Pt showed a significantly higher *k*_cat_ than LOx (1.52 × 10^6^ s^–1^ and 0.07 s^–1^, respectively), indicating a faster and more efficient catalytic activity (*k*_cat_/K_M_ = 9.9 × 10^7^ M^–1^ s^–1^ for Pt and 70 M^–1^ s^–1^ for LOx). These results are consistent with a scenario in which lactate is first oxidized to H_2_O_2_ (slower LOx step) and then is rapidly decomposed into O_2_ at the Pt surface (faster step).

Once the lactate consumption was confirmed, the motion of the nanomotor was studied (i) in response to different lactate concentrations (Figure 2C) and (ii) in the presence of culture media of melanoma cells (Figure 2D). For that purpose, the NanoSight N2300 equipment, the nanoparticle tracking analysis (NTA) software, and a custom R macro were employed. NTA software was used to extract the *x* and *y* coordinates of each particle, which were then processed to calculate its mean square displacement (MSD). By plotting MSD as a function of the time increment (Δt), a linear correlation was obtained, from which the diffusion coefficients of the nanomotor under different conditions were determined (D_0_ and D_eff_, nanoparticles in the absence and the presence of lactate, respectively).

The lactate concentrations used for motion analysis were selected based on the levels detected in SK-MEL-103 cells after 24 h of culture (0.25 mM). In addition, the propulsion of the nanomotor was evaluated using 1 mM lactate. This value is above the reported K_M_ for LOx (0.5–1 mM), allowing near-maximal enzymatic activity without reaching substrate saturation,^34^ moreover, it remains within physiologically relevant ranges,^35^ thereby ensuring efficient catalytic performance under biologically realistic conditions. NM­(dox)-LOx exhibited Brownian motion in the absence of lactate, with a D_0_ of 1.7 ± 0.2 μm·s^–1^ (Figure 2C-i and C-ii, light blue) and short trajectories (Figure 2C-iii, light blue). Conversely, 0.25 mM lactate produced an increase in NM­(dox)-LOx diffusion, reaching a D_eff_ value of 6.7 ± 0.9 μm^2^ s^–1^ (Figure 2C-i and C-ii, blue), corresponding to an approximately 4-fold increase in diffusivity upon fuel addition. This was accompanied by longer random spatial trajectories (Figure 2C-iii, blue). After incubation with 1 mM lactate, the D_eff_ of the nanomotor reached a value of 5.9 ± 1.0 μm^2^ s^–1^, (Figure 2C-i to C-iii, dark blue). These data highlight that NM­(dox)-LOx shows enhanced non-Brownian diffusivity in the presence of lactate.

To more rigorously assess the robustness of nanomotor propulsion under physiological relevant conditions, we analyzed their motility in cell culture medium (DMEM), which is devoid of lactate, and in the supernatant of SK-MEL-103 melanoma cells after 24 h of incubation, characterized by endogenous lactate secretion (Figure S7). In DMEM, NM­(dox)-LOx exhibited Brownian motion with a D_0_ value of 1.9 ± 0.2 μm^2^ s^–1^ (Figure 2D-i and D-ii, light blue) and short trajectories (Figure 2D-iii, light blue), which is consistent with the absence of fuel. In contrast, the nanomotor displayed enhanced diffusivity when suspended in the SK-MEL-103 supernatant with a D_eff_ value of 7.4 ± 0.6 μm^2^ s^–1^ (Figure 2D-i and D-ii, dark blue) and more extensive trajectories (Figure 2D-iii, dark blue). These findings demonstrate that the endogenous lactate generated by SK-MEL-103 cells provides an adequate metabolic substrate to sustain nanomotor propulsion.

To validate the pivotal contribution of lactate catalysis to nanomotor propulsion, the LOx enzyme was thermally inactivated, yielding the NM­(dox)-LOx-i variant. This catalytically inactive nanoparticle was unable to oxidize lactate, as evidenced by its near-absent LOx enzymatic activity (Table S7). As a result, NM­(dox)-LOx-i exhibited a Brownian behavior in the presence of lactate (D_0_ value of 1.8 ± 0.1 μm^2^ s^–1^ for 0.25 mM of lactate and 1.9 ± 0.1 μm^2^ s^–1^ for 1 mM of lactate), with no significant changes in MSD, D_0_, or trajectory patterns compared to PBS (D_0_ value of 1.6 ± 0.1 μm^2^ s^–1^), (Figure S8). Likewise, no propulsion was detected upon incubation of the inactive NM­(dox)-LOx-i with the culture media of SK-MEL-103 melanoma cells (D_0_ value of 1.5 ± 0.2 μm^2^ s^–1^ with DMEM and D_0_ of 2.0 ± 0.2 μm^2^ s^–1^ with cell supernatant, Figure S9), confirming that LOx activity is essential for lactate-induced autonomous propulsion.

Taken together, these findings demonstrate that the sequential decomposition of lactate and H_2_O_2_ on the anisotropic surface of Janus snowman-type nanomotors generates a propulsive force, most likely through a self-diffusiophoretic mechanism.^36^ Overall, the NM­(dox)-LOx nanomotor enables (i) protease-responsive and spatially controlled Dox release, (ii) efficient lactate depletion, and (iii) autonomous propulsion driven by catalytic cascade reactions at physiological lactate levels, a biocompatible metabolic fuel abundant within the TME.

### 2.4 Nanomotor-Mediated Cytotoxicity, Cellular Uptake in 2D and Tissue Penetration in 3D Melanoma Models

After confirming the lactate-driven capacity of NM­(dox)-LOx, we moved on to study its anticancer effect in melanoma models (human SK-MEL-103 and murine B16–F10 cell lines). We first confirmed that the hybrid cascade reaction in NM-LOx promoted ROS generation in SK-MEL-103 cells (Figure S10). Moreover, to ascertain whether nanomotor self-propulsion translates into superior biological efficacy, we investigated its impact on cellular internalization and cytotoxicity in both 2D and 3D melanoma cell cultures (Figure 3). To evaluate the internalization capability of the nanomotor by CLSM, both the nanomotor and the passive control (lacking enhanced movement) were labeled with a fluorophore, yielding NM*-LOx and NM*-LOx-i, respectively. CLSM images shown in Figure S11 confirmed the enhanced intracellular localization of NM*-LOx compared to the passive nanoparticle in SK-MEL-103 cells, demonstrating that movement improved cell internalization in 2D culture. The successful endocytosis of the nanomotor was also verified by TEM imaging, which revealed that the Janus morphology of the nanodevices was preserved inside the cells (Figure S12).

**Figure 3:**
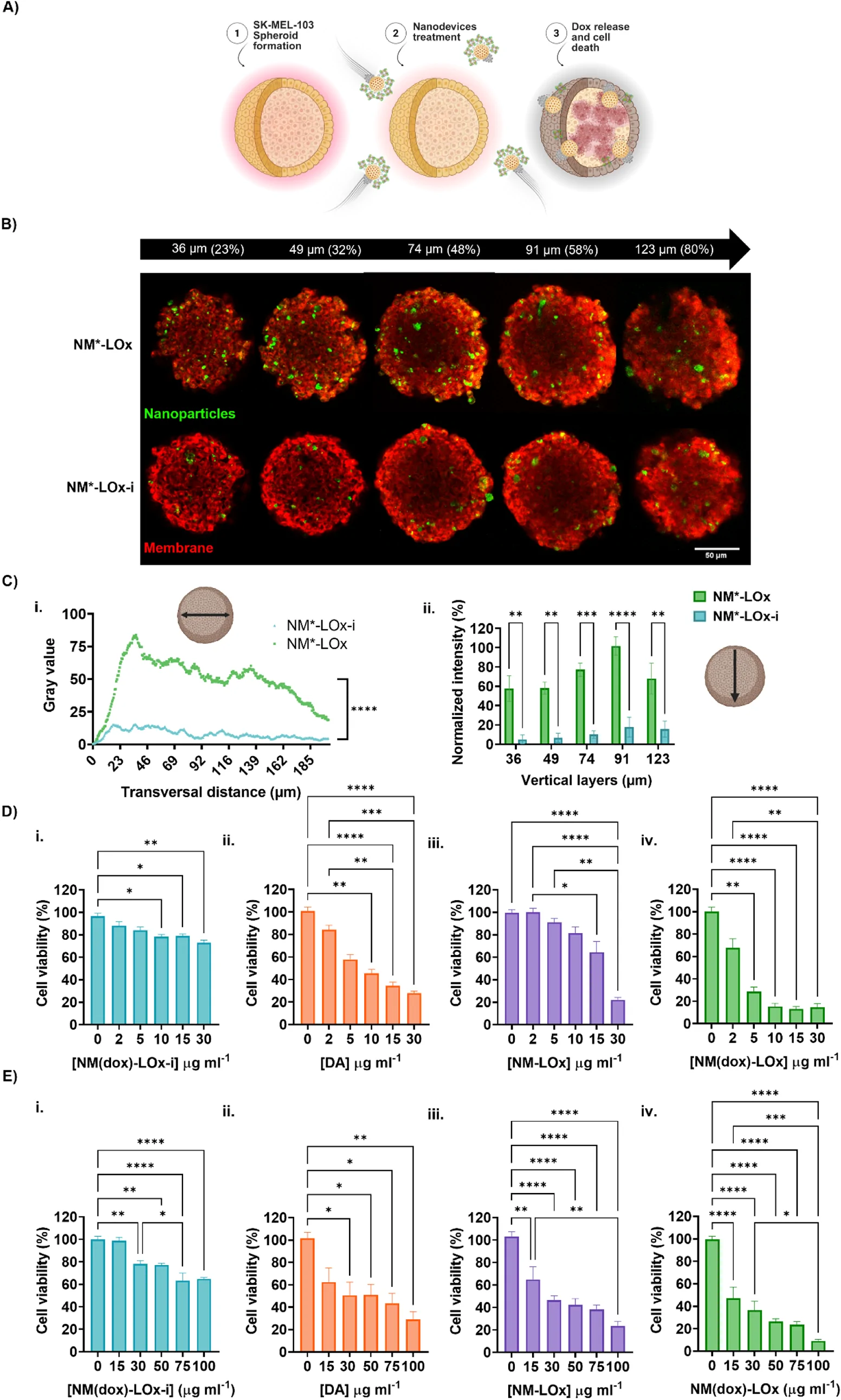
Analysis of nanomotor-mediated cytotoxicity and tissue penetration in 2D and 3D melanoma models. A) Scheme of nanomotor penetration in melanoma spheroids. B) CLSM images of representative spheroids upon treatment with NM*-LOx (top) and NM*-LOx-i (bottom) at 50 μg mL^–1^ for 24 h. Images are ordered from the outer (36 μm, 23% of total depth) to the deepest (123 μm, 80% of total depth) layer of the spheroids (red: cell membrane; green: nanoparticles). Scale bar: 50 μm. C) Quantification of fluorescence intensity in (i) the transverse axis (along the diameter of the spheroid); and (ii) the longitudinal axis (average intensity for layers 36, 49, 74, 91, and 123 um). Both measurements were performed between 23% and 80% of the total diameter of 5 randomly selected spheroids. Analyses in graphs C-i and C-ii were carried out with the program ImageJ (NM*-LOx: light green, NM*-LOx-i: dark green). Data are presented as mean ± SEM, n = 3. Statistical significance was assessed using a two-way ANOVA Test (*p < 0.05, **p < 0.01, ***p < 0.001, ****p < 0.0001). D) Effect of nanodevices on the cell viability of SK-MEL-103 cells and E) spheroids. Cells were treated with increasing concentrations (from 2 to 30 μg mL^–1^ and from 15 to 100 μg mL^–1^) of (i) NM­(dox)-LOx-i (blue), (ii) DA (orange), (iii) NM-LOx (purple) and (iv) NM­(dox)-LOx (green) for 24 h. Data are presented as mean ± SEM, n = 4. Statistical significance was determined by a one-way ANOVA Test (*p < 0.05, **p < 0.01, ***p < 0.001, ****p < 0.0001).

In a further step, nanomotor penetration was assessed in SK-MEL-103 tumor spheroids, a 3D multicellular model that more accurately recapitulates tumor architecture *in vivo*.^37^ For this purpose, SK-MEL-103 spheroids were exposed to NM*-LOx and NM*-LOx-i for a 24 h incubation period (Figure 3A). Figure 3B shows representative CLSM images of spheroids subjected to each treatment (red fluorescence corresponds to cell membranes and green fluorescence to NM*-LOx or NM*-LOx-i) from a superficial layer (36 μm, 23% penetration of spheroid diameter) to a deeper layer (123 μm, 80% penetration of spheroid diameter). In spheroids treated with the passive NM*-LOx-i nanoparticle, the green fluorescence signal remained restricted to the outer sections, consistent with diffusion-limited penetration and poor active transport to the core of each layer. In contrast, spheroids exposed to the catalytically active nanomotor exhibited a pronounced green fluorescence in the central core of each section and across the deepest layers, indicative of enhanced penetration driven by active motility overcoming the diffusional constraints of the 3D matrix. These differences were analyzed in Figures 3C-i, C-ii, S13, and S14. The fluorescence profile of each layer (transversal axis, Figure 3C-i) and the quantification of total fluorescence intensity across sections from the outermost to the innermost layers (longitudinal axis, Figure 3C-ii) revealed significant differences between NM*-LOx (green) and NM*-LOx-i (dark green). The active nanomotors exhibited enhanced penetration into the innermost layers of the spheroid (74–123 μm, 48–80% of the depth), where they accounted for approximately 75% of the fluorescence signal attributed to active nanomotor (Figure S14). This superior infiltration, ascribed to their lactate-driven self-propulsion within the spheroid microenvironment, contrasts sharply with the minimal penetration (<10%) achieved by inactive nanoparticles.

Regarding the cytotoxic effect, the final nanomotor (NM­(dox)-LOx, green), the control nanoparticles lacking a key component (NM-LOx (lacking Dox), purple; and NM­(dox)-LOx-i (lacking enhanced movement), blue) and the disassembled control (DA, orange) were tested in SK-MEL-103 cells (Figure 3D) and spheroids (Figure 3E) at 24 h. Figure 3D-iv shows a significant decrease in SK-MEL-103 cell viability to 28% upon treatment with NM­(dox)-LOx at a dose as low as 5 μg mL^–1^ (IC_50_ = 3 μg mL^–1^, Figure S15A and Table S9) in the presence of endogenous lactate produced by cancer cells. Increasing nanomotor concentrations further decreased cell viability, reaching 15% at 30 μg mL^–1^. In contrast, control nanoparticles did not induce comparable cytotoxicity effects showing IC_50_ values of 119.3, 8.4, and 18.4 for NM­(dox)-LOx-i, DA and NM-LOx, respectively (Figure S15A and Table S9). Viability assays in SK-MEL-103 spheroids, shown in Figure 3D, revealed that NM­(dox)-LOx significantly reduced cell viability to 47% at 15 μg mL^–1^, to 26% at 50 μg mL^–1^ and to 9% at 100 μg mL^–1^ (IC_50_ = 14 μg mL^–1^, Figure S15B and Table S9), whereas IC_50_ values were significantly higher for control nanoparticles (155.4, 37.6, and 30.9 for NM­(dox)-LOx-i, DA and NM-LOx, respectively).

Additionally, the cytotoxic capability of NM­(dox)-LOx was evaluated in B16–F10 spheroids, a murine melanoma model widely used for immunotherapy studies.^38–40^ In this model, the nanomotor was able to penetrate and release Dox, displaying an IC_50_ of 2 μg mL^–1^ (Figure S16 and Table S10), a value even lower than that observed in SK-MEL-103 spheroids. This increased sensitivity is likely attributable to the reduced structural compactness and lower mechanical resistance characteristics of spheroids derived from this cell line.

Overall, these findings demonstrate that NM­(dox)-LOx was the only construct exhibiting a pronounced therapeutic effect, inducing substantial cancer cell death even at very low concentrations. This enhanced efficacy is consistent with the synergistic interplay of lactate-driven self-propulsion, which facilitates enhanced and deeper penetration in cells and spheroids; LOx-mediated ROS generation; and protease-triggered drug release within lysosomal compartments in cells.

### 2.5 Evaluation of Nanomotor-Mediated Anticancer Activity in Human Melanoma Xenografts

Given the enhanced therapeutic effect showed by the nanomotor *in vitro*, we next evaluated its antitumor efficacy *in vivo* in a stepwise manner. First, a melanoma xenograft mouse model was selected to assess cytotoxicity and intratumoral penetration. This strategy allows for a clear mechanistic understanding before moving in the next section to a more complex, systemic and immunocompetent model. To this end, human SK-MEL-103 cells were subcutaneously injected into both flanks of athymic Nude-Foxn1nu mice. Once the tumors were formed, mice were randomly divided into 5 groups (n = 6 animals per group), and treatments were initiated. The groups consisted of the untreated control (i); the inactive nanodevice, NM­(dox)-LOx-i (ii); the nanomotor lacking Dox, NM-LOx (iii); the unassembled components, DA (iv); and the complete nanomotor, NM­(dox)-LOx (v). The nanosystems were administered intratumorally at a dose of 50 μg tumor^–1^ every other day for 1 week. Tumor progression and animal welfare were monitored daily until tumors in the control group reached the maximum size permitted by the institutional ethics committee, at which point mice were euthanized (Figure 4A).

**Figure 4:**
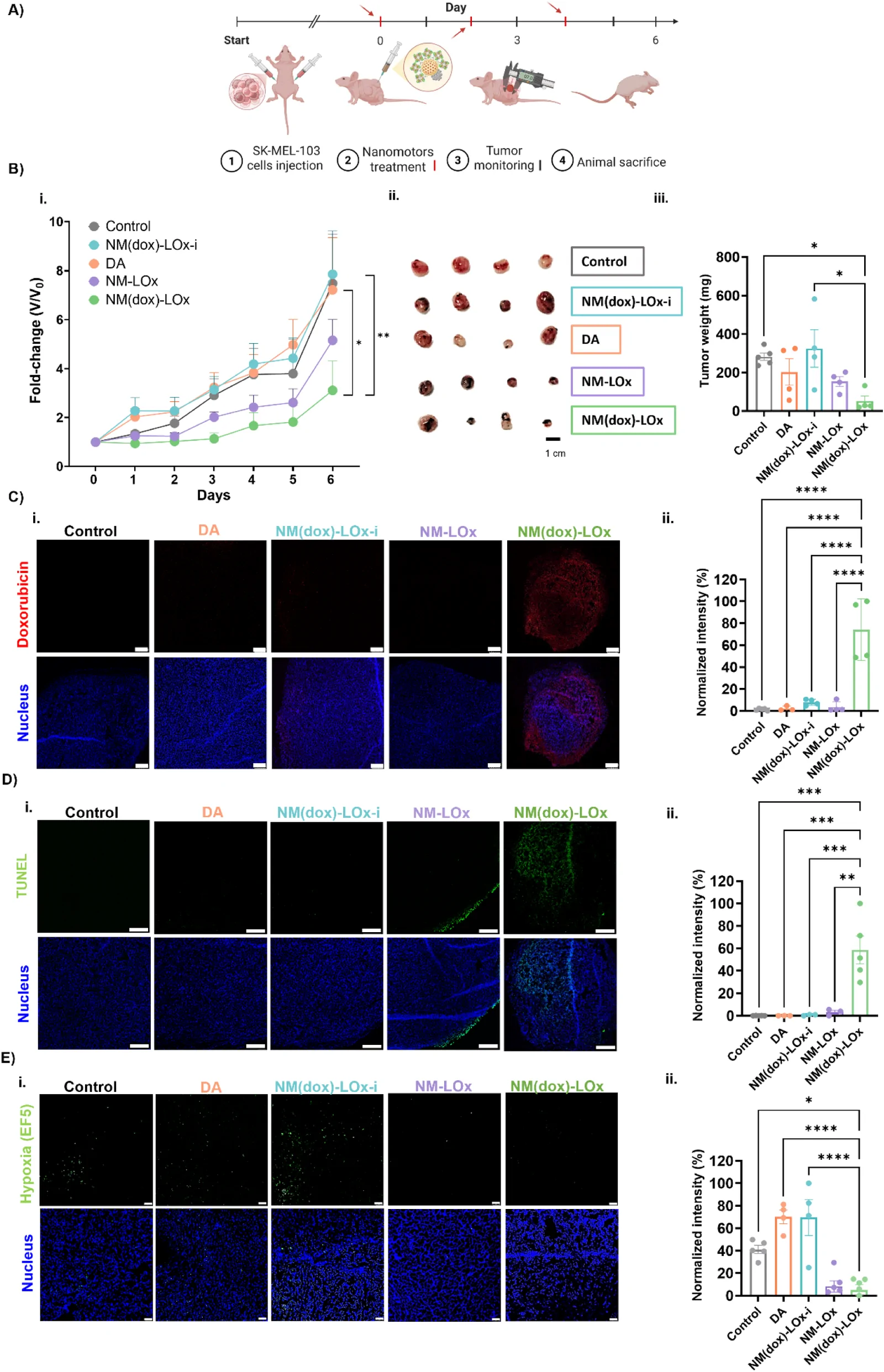
*In vivo* evaluation of the nanomotor therapeutic effect in a human melanoma xenograft model. A) Scheme of nanomotor therapy in athymic Nude-Foxn1nu mice. B-i) Monitorization of tumor growth after treatment with NM­(dox)-LOx (green), NM-LOx (purple), NM­(dox)-LOx-i (blue) or DA (orange), compared to control (gray). For each tumor, the fold-change represents the relative volume change compared to the baseline volume before treatment. Data are expressed as mean ± SEM, n = 6. Statistical significance was determined by a two-way ANOVA, followed by Tukey’s test (*p < 0.05, **p < 0.01). B-ii) Photographs of tumors at the end of the experiment. B-iii) Weight of tumors collected at the end of the experiment. Each data point represents an individual tumor, and bars denote mean ± SEM, n = 4. C-i) CLSM images of tumor slides from all treatment groups showing the release of Dox (red: Dox; blue: nucleus). Scale bar: 250 μm. C-ii) Quantification of Dox-related fluorescence signal. Data expressed as mean ± SEM, n = 4. D-i) CLSM images of the TUNEL assay used for the detection of apoptotic cells in tumor sections from all treatment groups (green: apoptotic cells; blue: nucleus). Scale bar: 250 μm. D-ii) Quantification of TUNEL-related fluorescence signal. Data expressed as mean ± SEM, n = 4. E-i) CLSM images of hypoxia evaluation using EF5 in tumor slides from all treatment groups (green: hypoxic adducts; blue: nucleus). Scale bar: 50 μm. E-ii) Quantification of EF5 fluorescence signal. Data expressed as mean ± SEM, n = 4. Statistical significance was determined by a one-way ANOVA, followed by Tukey’s test (*p < 0.05, **p < 0.01, ***p < 0.001, ****p < 0.0001).

As shown in Figures 4B-i and S17, the complete nanomotor NM­(dox)-LOx (green line) significantly inhibited tumor growth, resulting in a 58% reduction compared to the untreated control (gray line). The nanomotor lacking the Dox payload (NM-LOx, purple line) also induced a measurable reduction in tumor growth (31% relative to the control), although its efficacy remained lower than that of NM­(dox)-LOx. The antitumor activity of NM-LOx is consistent with the intrinsic catalytic functions of the nanodevice, including lactate depletion and local O_2_ generation, that could be attributed to the elevated endogenous H_2_O_2_ levels, which shift the cascade equilibrium toward O_2_ production at the Pt surface. In contrast, the more pronounced tumor growth inhibition achieved with NM­(dox)-LOx likely arises from the synergistic contribution of these catalytic processes combined with protease-triggered Dox release. Conversely, NM­(dox)-LOx-i (blue line) and the nonassembled components (DA, orange line) failed to produce any significant therapeutic benefit, as tumor progression closely mirrored that of untreated controls. These findings highlighted the relevance of the nanomotor architecture to enable its therapeutic function. These results were also corroborated by photographs of tumors and weight of tumors collected at the end of the experiment (Figure 4B-ii and B-iii). Notably, the well-being of the animals during the treatment was not affected, as demonstrated by the absence of body weight loss (Figure S18).

Moreover, lactate concentrations in SK-MEL-103 tumor xenografts were quantified by NMR at 14.8 μmol g^–1^ tissue (Figure S19), confirming the presence of this metabolite in the tumor environment.^34^ Comparable results were obtained using a standard colorimetric assay, which yielded a value of 14.9 μmol g^–1^ tissue. To further provide *in vivo* evidence of lactate depletion, we quantified tumor lactate levels in nanomotor-treated tumors using the same assay, obtaining values of 12.7 μmol g^–1^ tissue. Together, these findings indicate a partial reduction in intratumoral lactate following nanomotor treatment, consistent with LOx-mediated activity.

TEM imaging revealed that the nanodevices were internalized by cancer cells across different tumor sections (Figure S20). Furthermore, CLSM images of tumors from animals treated with NM­(dox)-LOx revealed a markedly stronger red fluorescence signal, corresponding to Dox, compared with tumors treated with control nanoparticles (Figure 4C-i). Quantitative analysis of Dox-associated fluorescence intensity (Figure 4C-ii) corroborated that the nanomotor substantially enhanced intracellular Dox delivery *in vivo*. To assess whether targeted Dox release resulted in increased apoptosis, a TUNEL assay was conducted. Tumors treated with NM­(dox)-LOx exhibited elevated nuclear green fluorescence, indicative of apoptotic cells, relative to control groups (Figure 4D-i). Quantitative analysis of green fluorescence intensity confirmed that the nanomotor NM­(dox)-LOx significantly enhanced apoptosis within the tumor tissue (Figure 4D-ii).

In addition, Hematoxylin and Eosin (H&E) staining was performed to assess the overall impact of the nanomotor on tumor tissue. Tumors treated with NM­(dox)-LOx exhibited extensive necrotic areas, characterized by loss of nuclear structure and cellular debris, indicating substantial damage to both tumor cells and the extracellular matrix. In contrast, control nanoparticles induced only minimal histopathological alterations (Figure S21). Finally, a hypoxia detection assay was conducted to determine whether the catalytic activity driving nanomotor motion contributed to alleviating tumor hypoxia (Figure 2B). CLSM images (Figure 4E-i) revealed a marked reduction in EF5-derived green fluorescence signal, indicative of hypoxic adducts, exclusively in tumors treated with NM-LOx and NM­(dox)-LOx. Quantification of fluorescence intensity (Figure 4E-ii) confirmed that LOx-functionalized nanodevices significantly increased intratumoral O_2_ levels, thereby contributing to mitigating hypoxic conditions.

In summary, these results prove that the local administration of NM­(dox)-LOx effectively inhibited tumor growth in a human xenograft mouse model. This outcome confirms that the synergistic functionalities previously characterized for the nanomotor remain operative *in vivo*, collectively yielding a significant anticancer therapeutic effect.

### 2.6 Nanomotor Overcomes Tumor Barriers and Enhances Antitumor Immunity in a Syngeneic Murine Melanoma Model

Motivated by these promising results, we investigated the therapeutic potential of the nanomotor in an immunocompetent melanoma model, where both tumor response and the contribution of the immune system could be evaluated. As we mentioned above, the immunosuppressive TME constitutes a major barrier to effective therapy, limiting immune-mediated tumor clearance. Lactate plays a key immunosuppressive role, inhibiting the activity of cytotoxic T cells and NK cells, while promoting the polarization of macrophages toward an anti-inflammatory M2 phenotype.^41^ Recent studies have shown that lactate deprivation promotes an immunostimulatory state within the TME, enhancing antitumor efficacy.^42^ In this study, we aimed to determine whether coupling lactate depletion with self-propelled nanomotors could simultaneously enhance drug delivery and potentiate immune-mediated tumor clearance.

For this purpose, the syngeneic immunocompetent melanoma B16–F10 model was employed as a standard immunocompetent system for evaluating antitumor immune responses for this study. Murine B16–F10 melanoma cells were subcutaneously injected into the dorsal flank of C57BL/6 mice. After the tumors were established, mice were randomly divided in 4 groups (n = 11 mice per group) and treatments were started. The groups were: the untreated control (i); the nanomotor lacking Dox, NM-LOx (ii); the inactive nanodevice, NM­(dox)-LOx-i (iii); and the complete nanomotor, NM­(dox)-LOx (iv). The nanoparticles were administered intraperitoneally at a dose of 20 mg kg^–1^ every other day for 1 week. Tumor growth and animal welfare parameters were monitored daily until the control reached the humane end point (Figure 5A). As shown in Figures 5B-i and S22, NM­(dox)-LOx (green line) induced a markedly greater tumor growth inhibition compared with the control group (gray line). In contrast, control nanoparticles did not elicit a comparable antitumor effect. These findings were further supported by digital imaging and by the final tumor weight measurements obtained from excised tumors at the end of the study (Figure 5B-ii and B-iii). Moreover, CLSM of tumor sections revealed enhanced tumoral accumulation of Dox in animals treated with NM­(dox)-LOx compared to those treated with control nanoparticles (Figure 5C). Likewise, TUNEL assay of NM­(dox)-LOx-treated tumors indicated a marked increase in apoptotic cells (Figure 5D), whereas H&E staining showed pronounced tissue damage, in contrast to the relatively preserved morphology observed in control groups (Figure 6A). Taken together, these findings corroborate the capacity of the lactate-powered nanomotor to enhance drug delivery and promote cancer cell elimination in a mouse melanoma model, which is consistent with its performance in the human melanoma xenograft study detailed above. Moreover, the preservation of therapeutic efficacy under systemic (intraperitoneal) administration (versus the intratumoral administration in the above model) highlights the translational potential of this platform, indicating that its performance is not restricted to direct intratumoral delivery but can be leveraged through clinically relevant administration routes.

**Figure 5:**
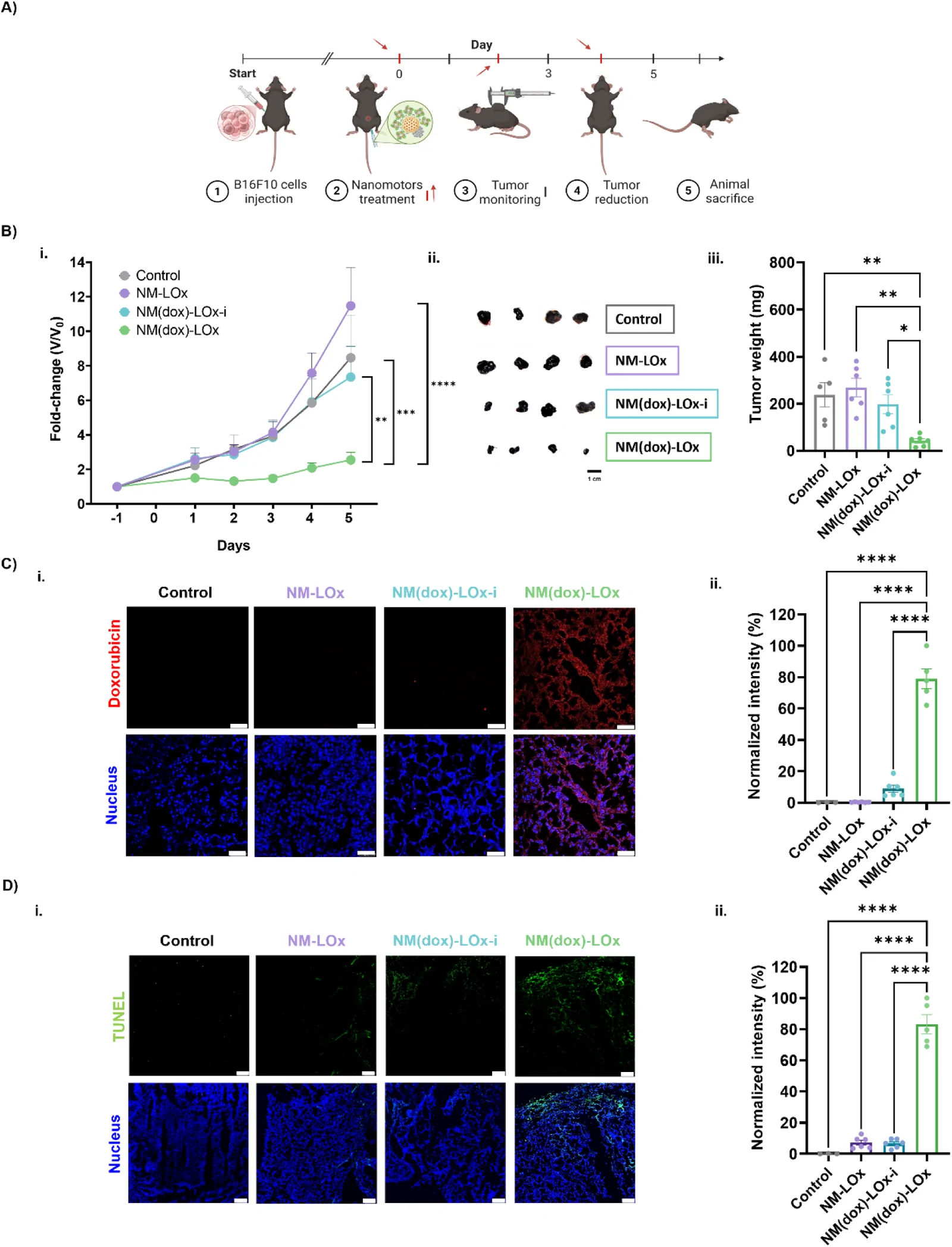
*In vivo* evaluation of nanomotor in a syngeneic melanoma model. A) Schematic representation of nanomotor-based therapeutic strategy in C57BL/6 mice. B-i) Monitorization of tumor growth during treatment with NM­(dox)-LOx (green), NM-LOx (purple) and NM­(dox)-LOx-i (blue) comparted to control group (red). Fold-change represents the relative change compared to the baseline volume prior to treatment initiation. Data are presented as mean ± SEM, n = 11. B-ii) Representative images of excised tumors at the end of the experiment. B-iii) Final tumor weight collected from B16–F10 melanoma-bearing mice after treatment. Each data point represents an individual tumor, and bars denote mean ± SEM, n = 5. C-i) CLSM images of tumor slides from groups: NM­(dox)-LOx (green), NM-LOx (purple) or NM­(dox)-LOx-i (blue), compared to control (gray), showing the release of Dox (red: Dox; blue: nucleus). Scale bar: 50 μm. C-ii) Quantification of Dox-related fluorescence signal. Data expressed as mean ± SEM, n = 4. D-i) CLSM images of the TUNEL assay used for the detection of apoptotic cells in tumor sections from all treatment groups (green: apoptotic cells; blue: nucleus). Scale bar: 100 μm. D-ii) Quantification of TUNEL-related fluorescence signal. Data expressed as mean ± SEM, n = 5. Statistical significance was determined using one-way ANOVA followed by Tukey’s multiple comparisons test (****p < 0.0001).

**Figure 6:**
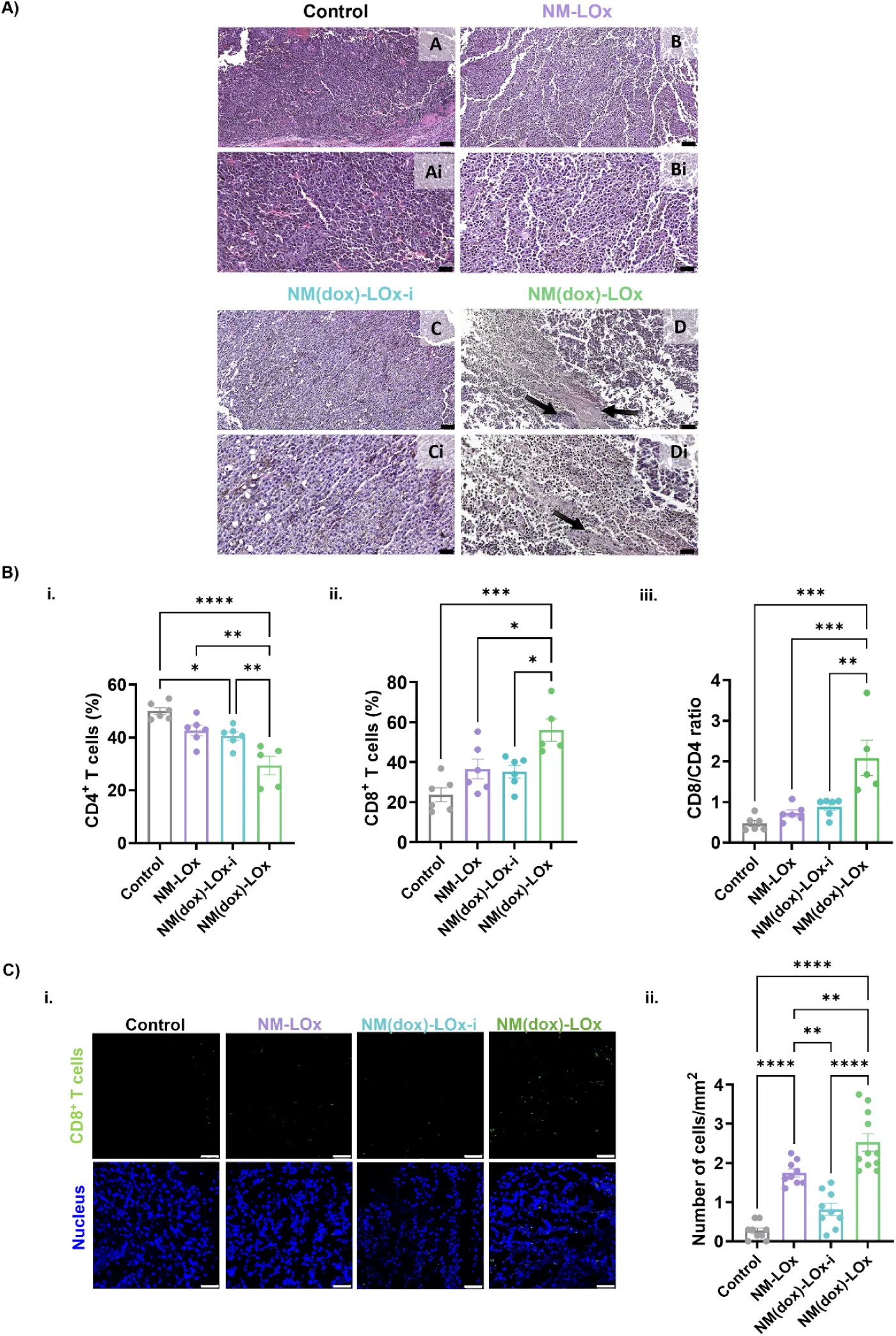
*Ex vivo* evaluation of nanomotor-induced immune system activation in a syngeneic melanoma model. A) H&E-stained tumor sections from melanoma mice model treated with A) Vehicle, B) NM-LOx, C) NM­(dox)-LOx-i and D) NM­(dox)-LOx. Necrotic regions are clearly observed in NM­(dox)-LOx-treated tumors (marked with an arrow). Scale bars: A-D, 100 μm and Ai-Di, same area enlarged to 50 μm. B-i) Quantification of tumor CD8^+^ T cells. B-ii) Quantification of tumor CD4^+^ T cells. B-iii) Resulting CD8^+^ /CD4^+^ ratio. C) Immunofluorescence analysis of tumor-infiltrating CD8^+^ T cells. C-i) CLSM images of tumor sections (green: CD8^+^ lymphocytes; blue: nucleus), scale bar: 50 μm. C-ii) Quantification of positive cells. Data are expressed as mean ± SEM, n = 5. Statistical significance was determined by a one-way ANOVA, followed by Tukey’s test (*p < 0.05, **p < 0.01, ***p < 0.001, ****p < 0.0001).

Moreover, to determine whether coupling lactate depletion with self-propelled nanomotors could additionally potentiate immune-mediated tumor clearance we evaluated the impact of nanomotor treatment on tumor-infiltrating lymphocytes. To this end, tumors excised at the experimental end point were processed for analysis by flow cytometry (Figures 6B and S23) and immunofluorescence (Figure 6C). Flow cytometry revealed a distinct shift in the immune cell population following the different treatments. Specifically, NM­(dox)-LOx-treated tumors exhibited a marked increase in CD8^+^ T cell infiltration, approximately 1.6-fold higher than in the inactive NM­(dox)-LOx-i control and the unloaded NM-LOx nanomotor, and 2-fold higher than in untreated tumors, indicative of a substantially enhanced cytotoxic immune response (Figure 6B-i). In contrast, CD4^+^ T cell frequencies were significantly reduced (∼1.7-fold relative to untreated tumors), suggesting a decrease in immunosuppressive or regulatory subsets (Figure 6B-ii). This remodeling resulted in a markedly higher CD8^+^/CD4^+^ ratio in NM­(dox)-LOx-treated tumors compared to controls (Figure 6B-iii). A higher ratio reflects a shift toward a more cytotoxic and less immunosuppressive T-cell landscape, a hallmark of a strengthened antitumor immune response. Similarly, e*x vivo* immunofluorescence staining of the tumor sections confirmed an increased presence of CD8^+^ T cells in tumors exposed to NM­(dox)-LOx compared to controls (Figure 6C), consistent with flow-cytometry results. These findings indicate that tumor lactate depletion mediated by LOx activity in the nanomotor promotes the recruitment and activation of effector T cells, contributing to the observed tumor growth suppression.

### 2.7 Nanomotor Biocompatibility and Biodistribution

Regarding the biocompatibility of the nanoparticles in B16–F10 melanoma-bearing C57BL/6 mice, no body weight loss was observed during the treatment period (5 days), suggesting the absence of nanomotor-induced toxicity upon systemic administration (Figure S24). Moreover, hematological and biochemical analyses performed at the study end point confirmed the absence of systemic toxicity. Liver and kidney function markers revealed no signs of organ dysfunction. The hematological levels remained within the established reference ranges for healthy murine models (there was no thrombocytopenia or notable abnormalities), indicating that the treatment with NM­(dox)-LOx did not induce adverse physiological alterations (Figure S25). Likewise, H&E staining of major organs (heart, kidney, liver, lung, and spleen) revealed no apparent histopathological abnormalities across the treatment groups. All organs displayed preserved histological architecture, including intact myocardial fibers, normal hepatic lobular organization, regular splenic white and alveolar morphology, and renal cortical and medullary organization. No evident necrosis or structural disorganization was observed in any group, confirming the absence of systemic toxicity induced by the treatments (Figure S26).^43^

To complement the above study, we further evaluated the biodistribution and potential long-term toxicity of the nanoparticles, using NM-LOx (without Dox), over a 15-day period. To this end, we intraperitoneally administered a single dose of NM-LOx (lacking Dox) to healthy C57BL/6 mice (n = 6) at 60 mg kg^–1^ (the cumulative dose administered during the therapeutic study in tumor-bearing C57BL/6 mice). We monitored the body weight and well-being of the animals for 15 days, without observing any clinical signs of distress or weight loss (Figure S27). Then, we collected blood samples to perform hematological and biochemical analyses and sacrificed the animals to harvest their major organs. Blood tests showed no relevant differences in the parameters studied in treated animals compared to controls (Figure S28). Similarly, H&E staining of major organs did not show any apparent morphological alterations in animals receiving NM-LOx (Figure S29). In addition, nanoparticle biodistribution was assessed by quantifying platinum levels in different organs using inductively coupled plasma mass spectrometry (ICP-MS) (Figure S30). Pt was detected in the liver and spleen (69.3 ± 14.4 and 231.3 ± 17.7 mg per kg of tissue, respectively). This outcome is expected, as Pt and MSN nanoparticles^44–47^ commonly accumulate in detoxification organs associated with the reticuloendothelial system (RES), indicating partial clearance and/or degradation of the nanodevice, likely through hepatic processing. Altogether, these results suggest that the nanomotor does not induce adverse effects in mice at the dosage regimen used.

## 3 Conclusion

In summary, NM­(dox)-LOx was successfully synthesized, thoroughly characterized, and validated both *in vitro* and *in vivo* using human SK-MEL-103 and murine B16–F10 melanoma models (2D cultures, 3D tumor spheroids, and animal studies). This lactate-powered nanomotor integrates catalytic self-propulsion, using lactate as a biocompatible and readily available metabolic fuel, with protease-responsive doxorubicin delivery. Through LOx functionalization, the nanomotor consumes tumor-derived lactate to generate H_2_O_2_, which is subsequently decomposed on its Pt face into oxygen and water. This catalytic cascade, together with the anisotropic Janus architecture, drives enhanced diffusive propulsion via a self-diffusiophoretic mechanism. Additionally, LOx functions as a protease-sensitive gatekeeper, enabling efficient and spatially confined Dox release upon exposure to lysosomal proteases.

Consequently, NM­(dox)-LOx exhibits a remarkable therapeutic effect, significantly reducing tumor volume in different *in vivo* melanoma models under both local and systemic administration. This antitumor efficacy arises from the synergistic combination of the nanomotor capabilities. Its engineered nanoarchitecture and lactate-driven propulsion promote efficient diffusion across the tumor ECM, resulting in deep tissue penetration and enhanced cellular internalization. The improved reach to cancer cells (located in otherwise inaccessible tumor areas) together with its ability to intracellularly deliver Dox, leads to their efficient elimination. In parallel, the nanomotor ability to generate ROS (which also induces cancer cell death) and local O_2_ production in the TME (partially reversing tumor hypoxia and restoring normoxic conditions) further contributes to its therapeutic performance. Notably, the catalytic depletion of lactate reprograms the TME toward an immunostimulatory state, promoting cytotoxic immune infiltration, as evidenced by increased CD8^+^ T cell infiltration and a concomitant reduction in CD4^+^ T cell populations.

Overall, NM­(dox)-LOx emerges as a promising multifunctional nanoplatform capable of coupling metabolic remodeling with immune reactivation and chemotherapy, offering an approach for the development of self-propelled therapeutic nanosystems for cancer treatment. By simultaneously improving drug delivery through the ECM and promoting immune activation, NM­(dox)-LOx effectively overcomes structural and metabolic barriers of solid tumors, resulting in pronounced tumor regression without systemic toxicity, highlighting its potential to improve therapeutic outcomes and guide the design of future precision nanomedicine strategies.

## 4 Experimental Section

### 4.1 Synthesis of Nanoparticles

#### 4.1.1 Mesoporous Silica Nanoparticles (MSNs)

480 mL of deionized water were used to dissolve 1 g (2.74 mM) of n-cetyltrimethylammonium bromide (CTABr). The pH was raised by adding 3.5 mL of NaOH 2 M and the temperature was elevated to 80 °C. Then, 5 mL of the tetraethyl orthosilicate (TEOS) were dropped into the solution and the mixture reacted for 2 h under magnetic stirring. The resulting solid was centrifuged, rinsed with deionized water until neutral pH and dried at 70 °C. Finally, the solid was subjected to a 5-h calcination process at 550 °C in an oxidizing atmosphere to obtain the final mesoporous silica nanoparticles (**MSNs**).

#### 4.1.2 Platinum Nanoparticles (PtNPs)

164 mg of H_2_PtCl_6_ and 20 mg of polyvinylpyrrolidone (PVP) were dissolved in 20 mL of deionized water. Then, 10 mL of a 0.2 M ascorbic acid aqueous solution were added drop by drop and the mixture was heated at 45 °C for 3 h under magnetic stirring. The resulting black color solution indicated the successful synthesis of platinum nanoparticles (**PtNPs**).

#### 4.1.3 Janus Pt-MSN (NM)

180 mg of MSNs were dispersed in 10 mL of a solution containing 6.7% ethanol. Then, 208 μL of CTAB (1 μM) solution were added to the silica suspension, and the mixture was heated to 80 °C with stirring. Then, 1 g of paraffin was incorporated and melted. The mixture was homogenized with an Ultra-Turrax T-8 (IKA, Germany) at 25,000 rpm for 15 min. To form a stable Pickering emulsion, the suspension was magnetically stirred for 1 h. After cooling to room temperature, the mixture was sonicated and transferred to a round-bottom flask. Then, 180 μL of 3-(mercaptopropyl)­trimethoxysilane and 10 mL of methanol were added, and the reaction was maintained under stirring for 3 h. The resulting silanized solid was centrifuged out and rinsed with methanol. Then, it was suspended in 68 mL of methanol and combined with 30 mL of PtNPs. After overnight stirring at room temperature, the solid was washed with ethanol and chloroform, filtered under vacuum and dried to obtain Janus Pt-MSN nanoparticles (**NM**).

#### 4.1.4 NM­(dox)-LOx

Twenty-five mg of NM were dispersed in 3 mL of anhydrous acetonitrile (ACN), followed by the addition of 25 μL of (3-aminopropyl)­triethoxysilane (APTES). The suspension was stirred for 5.5 h and the resulting material (NM-NH_2_) was washed with ACN, ethanol, and 100 mM sodium phosphate buffer (PBS, pH 7.5). Drug loading was carried out by dissolving 12.5 mg of doxorubicin (Dox) in 3 mL of PBS and adding it to the APTES functionalized solid. The mixture was kept under magnetic stirring for 24 h in the dark.

For the enzyme-gating step, the acidic groups of lactate oxidase (LOx) were activated by mixing 6.25 mg of LOx, 6.25 mg of *N*-hydroxysuccinimide (NHS) and 6.25 mg of N-(3-(dimethylamino)­propyl)-N′-ethylcarbodiimide hydrochloride (EDC) in 3 mL of PBS. The activation proceeded at 4 °C for 30 min with gentle stirring, after which the enzyme was washed with 100 mM PBS (pH 6) using Amicon Ultra 0.5 mL 10 K filters. Subsequently, the activated LOx was coupled to 25 mg of the amino-functionalized material. The reaction was allowed to proceed overnight at 4 °C with magnetic stirring in PBS. The final product (**NM­(dox)-LOx**) was obtained after centrifugation and washing with PBS.

#### 4.1.5 NM­(dox)-LOx-i

these control nanoparticles were synthesized as described for NM­(dox)-LOx but were subjected to a heat treatment to deactivate the catalytic activity of LOx. To do this, NM­(dox)-LOx was exposed at 70 °C for 1 h. The resulting solid was **NM­(dox)-LOx-i**.

#### 4.1.6 NM-LOx

these control nanomotors were synthesized fusing a procedure analogous to that of NM­(dox)-LOx, except that the drug loading step was omitted. The resulting material was designated **NM-LOx**.

#### 4.1.7 NM*-LOx

the fluorophore for labeling was synthesized as an APTES-functionalized 3,4,9,10-perylenetetracarboxylic dianhydride diimide derivative (PTDA) following the protocol described in a previous study.^47^ Then, 5 mg of NM-NH_2_ were dispersed in 2 mL of chloroform, and 5 mg of PTDA were added. The mixture was stirred magnetically for 5.5 h. The labeled nanomotors were then purified by centrifugation with chloroform, ethanol, and PBS. Enzyme immobilization was subsequently performed following the same procedure as described for NM­(dox)-LOx, producing (NM*-LOx).

#### 4.1.8 NM*-LOx-i

these control nanoparticles were synthesized as described as NM*-LOx but were subjected to heat treatment to deactivate the catalytic activity of LOx. To do this, NM*-LOx was exposed to at 90 °C for 15 min to obtain NM*-LOx-i.

### 4.2 Characterization of Nanoparticles

#### 4.2.1 Determination of LOx Enzyme

The amount of the enzyme LOx bound to the mesoporous face of the nanoparticles was determined using the BCA technique. For 10 min at 60 °C, 200 μL of B/A solution at a proportion 1:50 was incubated with 10 μL of the corresponding nanoparticles (1 mg mL^–1^). Then, the samples were centrifuged, and the supernatant’s absorbance was measured at a wavelength of 570 nm. The calibration line was created using various free BSA concentrations in PBS 100 mM at pH 7.5 (from 7.8 to 1000 μg mL^–1^).

#### 4.2.2 Controlled Doxorubicin Release Assays

In the presence of proteases from *Streptomyces griseus:* for the *in vitro* controlled release studies, 1 mg of NM­(dox)-LOx were dispersed in 2 mL of PBS (100 mM, pH 7.5) and separated into two equal portions. Both suspensions were incubated at 37 °C under gentle shaking. To one of the fractions, 4 mg of protease from *Streptomyces griseus* were added to trigger enzyme-mediated cargo release. At predetermined intervals (5, 10, 15, 30, 60, and 120 min), aliquots were collected and centrifuged (12,000 rpm, 3 min) to remove the nanoparticles. The supernatants were analyzed by fluorescence spectroscopy to determine the amount of doxorubicin released to the medium (λ_exc_ = 470 nm and λ_em_ = 555 nm).

Forced release: to quantify the total doxorubicin content within the nanoparticles, forced-release experiments were performed. In this assay, 0.5 mg of the corresponding nanoparticles were suspended in 1 mL of DMSO and stirred in the dark for 24 h to ensure complete drug extraction. After centrifugation, the absorbance of the supernatant was measured at λ_abs_ = 495 nm, and the doxorubicin concentration was calculated from a calibration curve, using Lambert–Beer’s law (eq 1).

1 A=ε·c·l

Equation 1. A represents absorbance; c represents the molar concentration (M); l represents the optical light path (cm); ε represents the doxorubicin molar absorption coefficient (9.250 mM^–1^ cm^–1^).

In the presence of lysosomal extract: to investigate cargo release under conditions mimicking the cellular endocytic environment, 1 mg of NM­(dox)-LOx were dispersed in 1 mL of purified lysosomal extract. The lysosomal extract was prepared from fresh rabbit liver following the instructions provided in the Lysosome Isolation Kit (LYSISO1) and diluted 1:4 prior to use. As a control, an equivalent amount of nanoparticles were suspended in 1 mL of 10 mM PBS (pH 7.5). Both suspensions were incubated on a shaker at 37 °C, and at designated time points (5, 10, 15, 30, 60, 120, and 240 min), aliquots were removed and centrifuged (12,500 rpm, 3 min) to separate the nanoparticles. The supernatants were analyzed by fluorescence spectroscopy to determine the amount of doxorubicin released to the medium (λ_exc_ = 470 nm and λ_em_ = 555 nm).

#### 4.2.3 Determination of Catalytic Activities

Lactate oxidase activity assay: an enzymatic activity assay based on UV–visible spectrophotometry was carried out to ensure that the immobilization of LOx to nanoparticles did not impair its capacity to produce H_2_O_2_ from lactate. The ability of peroxidase from horseradish (HRP) to oxidize 2,2-azino-bis­(3-ethylbenzothiazoline-6-sulfonic acid (ABTS) with H_2_O_2_ (generated by LOx from lactate) to a blue/green product (ABTS^2–^, λ_abs_ = 405 nm) is the basis for this test.

In a common procedure, solutions of lactate 1 M (112.3 mg mL^–1^) and ABTS (1 mg mL^–1^) were prepared in 100 mM PBS at pH 7.5. Then, 250 μL of each solution were mixed in a quartz cuvette, and 10 μL of PBS (blank or negative control), 10 μL of LOx enzyme (positive control) or 10 μL of the suspension of nanomotors (50 mg mL^–1^) were added. After shaking the samples, absorbance was measured at 405 nm in accordance with time (2 min at 25 °C). The following chemical processes occur during the LOx enzymatic activity assay:

**Reactions**:

$$L-Lactate+O_{2\;}\underset{⃗}{\;\mathrm{LOx}}\;Pyruvate+H_{2}O_{2}$$

$$ABTS+H_{2\;}O_{2}\underset{⃗}{\;\mathrm{HRP}}\;ABTS^{\cdot +}+H_{2}O$$

Equation 2 was used to determine enzymatic activity:

2 $$\frac{Enzyme\;Units}{g}=\frac{\left(\Delta A-\Delta A_{blank}\right)\cdot V_{T}\cdot F_{D}}{\varepsilon_{ABTS}\cdot l\cdot V_{S}\cdot C_{S}}$$

Equation 2. ΔA: slope of the graph (min^–1^); ΔA_blank_: slope of the graph for the blank (min^–1^); V_T_: total volume in the cuvette; F_D_: dilution factor; ε_ABTS_: molar extinction of ABTS at 405 nm (36.87 M^–1^ cm^–1^); L: optical path (1 cm); V_S_: volume of the added sample (mL); C_S_: concentration of the sample (g mL^–1^).

With the results of this experiment (Table S7), the LOx concentration (determined from data obtained in BCA assay, 6.41 μM) and the K_M_ of the enzyme (1 mM), the kinetic parameters of LOx in NM-LOx were calculated using eqs 3–5.

3 $$\frac{1}{v}=\frac{K_{M}}{V_{max}}\cdot \frac{1}{\left[S\right]}+\frac{1}{V_{max}}$$

Equation 3: v: reaction velocity; K_M_: Michaelis–Menten constant; Vmax: maximum reaction velocity; S: concentration of the substrate.

4 $$K_{cat}=\frac{V_{max}}{\left[E\right]}$$

Equation 4. K_cat_: turnover rate; *V*_max_: maximum reaction velocity; [E]: molar concentration of the nanomotor, calculated as previous studies.^47^

5 $$Catalytic\;efficiency=\frac{K_{cat}}{K_{M}}$$

Equation 5. K_cat_: turnover rate; K_M_: Michaelis–Menten constant.

H_2_O_2_-reducting activity assay: 40 μL of nanomotors (1 mg mL^–1^) were mixed with 500 μL of ABTS (1 mg mL^–1^) and 500 μL of H_2_O_2_ (0–30 mM), and the increment in absorbance (λ_abs_ = 405 nm) was recorded for 2 min. Enzymatic-like Michaelis–Menten parameter (K_M_), maximum velocity (*V*_max_), turnover rate (K_cat_), and catalytic efficiency were calculated by applying eqs 3–5.

Measurement of lactate consumption: the supernatant of melanoma cells that were seeded in 96-well plates for 72 h was collected and mixed with NM­(dox)-LOx (15, 30, or 50 μg mL^–1^) for 1 h at 37 °C. After incubation, samples were centrifuged (12,500 rpm, 3 min) to remove debris and nanoparticles. Lactate concentration was quantified using the L-Lactate Assay Kit (MAK329, Merck) according to the manufacturer’s instructions. Briefly, 20 μL of each sample or lactate standard (0–1 mM) were dispensed into a clear 96-well plate in triplicate. Reaction mix was freshly prepared by combining assay buffer, Enzymes A/B, NAD, and MTT (as supplied in the kit) and 80 μL were added to each well. Plates were incubated for 20 min at room temperature, and absorbance was measured at 565 nm before and after this incubation using a microplate reader. Lactate concentrations were interpolated from a standard calibration curve and corrected for sample dilution.

#### 4.2.4 Evaluation of Nanomotors Motion

Nanomotors and control nanoparticles movement were characterized using the Nanosight NS300 with NTA software 3.0. Increasing concentrations of lactate (0, 0.25 mM and 1 mM) were first introduced into the chamber to prevent drift and after ultrasonication, NM­(dox)-LOx or NM­(dox)-LOx-i were added at 2 μg·mL^–1^ in PBS (pH 7.5, total volume 800 μL, 25 °C) at every lactate concentration tested. The same procedure was followed to evaluate the nanoparticles movement in DMEM and melanoma cell supernatant. Thirty-second videos were recorded at 30 fps using the microscope and sCMOS camera. Coordinates (x, y) of 25 nanoparticles were tracked every 0.1 s, excluding particles smaller than 200 nm or larger than 500 nm.

Mean square displacement (MSD) was calculated for each particle assuming 2D motion (eq 6), and an in-house R script determined the diffusion coefficient for the corresponding nanoparticles (D_eff_) and for control/nonfueled nanoparticles (D_0_) from MSD vs Δ*t*ime plots (eq 7). Spatial trajectories were also visualized.

6 $$MSD=\left\{{\left(x_{t}-x_{0}\right)}^{2}\right\}=\frac{1}{N}\overset{N}{\underset{i=0}{\sum }}{\left(x^{i}\left(t\right)-x^{t}\left(0\right)\right)}^{2}$$

Equation 6: where MSD is the mean square displacement, N is number of nanoparticles, and x^i^ and x^t^ are particles’ vector positions at different times.

7 $$MSD=\left(4D_{eff}\right)\cdot \Delta t|MSD=\left(4D_{0}\right)\cdot \Delta t$$

Equation 7: where MSD is the mean square displacement, D_eff_ is the effective diffusive coefficient for the active nanoparticles, D_0_ is the diffusion coefficient for the nonpropelled nanoparticles and Δt is the time increment.

### 4.3 Evaluation of Nanoparticle’s Anticancer Capabilities

#### 4.3.1 Melanoma Cell Culture

Human melanoma cell line, SK-MEL-103 (ATCC), and mouse melanoma cell line B16–F10 (ATCC), were grown in DMEM supplemented with 10% fetal bovine serum (FBS) (Sigma-Aldrich). These cells were incubated in an atmosphere of 5% of CO_2_ at 37 °C.

#### 4.3.2 Lactate Detection Assay

Extracellular concentrations of lactate in melanoma cells were measured using Lactate Assay Kit (Sigma; MAK064). SK-MEL-103 cells were seeded in 96-well plates at a density of 5,000 cells well^–1^ and incubated for 24 or 72 h. Then, the supernatant was collected. After 15 min, samples were centrifuged (13,000 rpm, 10 min) and deproteinized with 10 kDa MWCO spin filters. Soluble fraction was mixed with Master Reaction Mix which contained a mixture of enzymes and a colorimetric reactive. Lastly, the reaction was incubated for 30 min at room temperature and absorbance signal was measured at 570 nm.

#### 4.3.3 ROS Detection

The production of reactive oxygen species (ROS) was induced by treatment with NM-LOx (lacking doxorubicin to avoid toxicity) and was analyzed using a fluorescent detection probe based on DCFDA-H2DCFDA (C6827, Fisher scientific) reactive (λ_exc_ = 495 nm, λ_em_ = 529 nm).

SK-MEL-103 cells were seeded in a 6-well plate with a density of 500,000 cells mL^–1^ and incubated for 24 h. Then, cells were treated with NM-LOx at 50 μg mL^–1^ for 30 min. After that, cells were washed with PBS to remove noninternalized nanoparticles and were incubated for 30 min. ROS presence was measured by CLSM and flow cytometry. In both assays, DCFDA-H2DCFDA was added in PBS for 30 min at 5 μM. Then, the cells were washed and DMEM was added for another 30 min to stabilize the fluorescence signal. For CLSM, Hoechst 33342 and wheat germ agglutinin (WGA) Alexa Fluor 488 were added for nuclei and membrane staining, and CLSM images were obtained with Leica TCS SP8 confocal microscope. For flow cytometry, propidium iodide (PI) was added to select live cells and cumene hydroperoxide (50 μM) was used as a positive control. Cells were sorted by CytoFlexS instrument (Beckman Coulter).

#### 4.3.4 Cytotoxicity Assays in 2D Culture

WST-1 viability tests (11644807001, Sigma) were used to assess the cytotoxic effects of different nanoparticles on cancer cells. In 96-well plates, SK-MEL-103 cells were plated at a density of 50,000 cells mL^–1^ and incubated for 24 h. NM­(dox)-LOx, NM-LOx, NM­(dox)­LOx-i and the disassembled system (DA, consisting of a mixture of MCM, Pt, dox and LOX at equivalent concentration than that in NM­(dox)-LOx) were added at various concentrations (0, 2, 5, 10, 15, and 30 μg mL^–1^) and incubated for 30 min. Following, the noninternalized nanoparticles were removed via a washing procedure, and cells were incubated for 24 h. Then, the cell proliferation reagent WST-1 was added and the absorbance signal at 595 nm was measured.

#### 4.3.5 Nanoparticle’s Cell Internalization in 2D Culture

To evaluate the internalization of nanoparticles by CLSM, SK-MEL-103 cells were seeded in 6-well plates at a density of 500,000 cell mL^–1^ and incubated for 24 h. Next, cells were treated with NM*-LOx and NM*-LOx-i at a concentration of 50 μg mL^–1^ for 30 min and subsequently were washed with PBS (100 mM, pH 7.4) to remove noninternalized nanoparticles. Then, cells were incubated for 1, 2, and 3 h. The DNA marker, Hoechst 33342, was used to identify the nucleus and WGA Alexa Fluor 488 to mark the membrane. Images were obtained by CLSM (λ_ex_ = 470 nm, λ_em_ = 555 nm, magnifications 40x and 63x). Cell penetration evaluation was also performed by TEM microscopy. In Chamber slide, SK-MEL-103 cells were seeded at a density of 40,000 cells mL^–1^ and incubated for 24 h. Then, cells were treated with 50 μg mL^–1^ of NM-LOx for 30 min and washed with PBS. After that, cells were incubated in a solution of 3% glutaraldehyde for 10 min at 37 °C. Cells were washed and reincubated with 3% glutaraldehyde for 1 h at room temperature. Finally, cells were washed five times for 10 min with 100 mM PBS and were stained with uranyl acetate and osmium tetroxide to be included in epoxy resin.

#### 4.3.6 3D Tumor Spheroids Formation

To obtain 3D tumor spheroids of melanoma cells, we use the AggreWell 6-well plate system (17128081, Fisher Scientific). First, we pretreat wells with 500 μL of Anti-Adherence Rising Solution provided of starter kit of these plates. Plates were centrifugated at 300 rcf for 5 min. Then, antiadherence rising solution was aspired from wells after ensuring that bubbles have been removed from the microwells. One mL of DMEM with 10% FBS was added to wells. Cells were seeded at 1.8 × 10^6^ cells well^–1^ and complete with medium to achieve a final volume of 2 mL well^–1^. AggreWell plate was centrifugated at 100 rcf for 3 min to capture cells in the microwells. Cells were incubated at 37 °C and 5% CO_2_. Finally, successful 3D tumor spheroids formation was confirmed by optical microscopy after 24 h.

#### 4.3.7 Nanoparticle’s Cytotoxicity on Tumor Spheroids

To evaluate nanoparticleś cytotoxicity in melanoma spheroids, we proceed similarly to 2D cell culture assay. Spheroids were incubated with NM­(dox)-LOx, NM-LOx, NM­(dox)-LOx-i, and DA at concentrations from 0 to 100 μg mL^–1^. The next day, CellTiter-Glo Luminescent Cell Viability (G7571, Promega) assay was performed and the luminescence generated was measured to assess the cell viability.

#### 4.3.8 Nanoparticle’s Penetration on Tumor Spheroids

The penetration capacity of nanomotors within tumor spheroids was analyzed via CLSM. Spheroids of SK-MEL-103 cells were incubated with either NM*-LOx or NM*-LOx-i at 50 μg mL^–1^ for 24 h. After treatment, WGA (2 μg mL^–1^, 2 h) was added to stain the cell membrane. Imaging was carried out using a Leica TCS SP8MP multiphoton CLSM system, and fluorescence signals were quantified using ImageJ software. It should be noted that, due to the high opacity of spheroids, the microscope laser penetration was limited to 80% of the total spheroid diameter.

### 4.4 *In Vivo* Antitumoral Effect of Nanoparticles

#### 4.4.1 Animal Models

Female and male (10 and 8-week-old) Hsd: Athymic Nude-Foxn1nu nude and C57BL/6JOlaHsd mice were acquired from Envigo (Inotiv) and housed at the Príncipe Felipe Research Centre (València, Spain) under pathogen-free conditions. Animals were left in a room at controlled temperature inside ventilated polyethylene cages with stainless steel lids, were administered food and water *ad libitum* and subjected to alternate light/dark cycles lasting 12 h. Mice were treated according to the Guide for Care and Use of Laboratory Animals. All the procedures were approved by the Ethical Committee for Research and Animal Welfare of the Generalitat Valenciana, Conselleria d’Àgricultura, Medi Ambient, Canvi Climàtic i Desenvolupament Rural (2023-VSC-PEA-0049–01 and 2025-VSC-PEA-0027).

#### 4.4.2 Establishment of the Tumor Mouse Models

To generate the tumor mouse models, mice were first anesthetized with isoflurane before the implantation of cancer cells. Subsequently, 5 × 10^5^ SK-MEL-103 cells suspended in DMEM were subcutaneously administered into both flanks of each animal in Hsd: Athymic Nude-Foxn1nu (immunosuppressed tumor model) or 5 × 10^5^ B16–F10 cells were inoculated in one flank in C57BL/6JOlaHsd (immunocompetent tumor model). After injection, the mice were randomly allocated into the designated experimental groups. Tumor progression (length × width) was assessed every 2 days using a digital caliper. Tumor volume (mm^3^) was calculated according to next equation (eq 8):

8 $$Tumor\;volume=\frac{\left(length\cdot \left(width^{2}\right)\right)}{2}$$

#### 4.4.3 Mice Treatments

Immunosuppressed tumor model: therapeutic interventions commenced on day 6 postinoculation of SK-MEL-103 cells (designated as day 0), when the tumors were palpable (average volume of ∼25 mm^3^). Mice were randomly assigned into five groups (n = 6): the untreated control; the final nanomotor, NM­(dox)-LOx; the control nanomotor lacking doxorubicin, NM-LOx; the disassembled nanomotor (DA) and the inactive nanoparticles, NM­(dox)-LOx-i. Each treatment consisted of intratumoral injections at a dose of 2 mg kg^–1^ (50 μg and 25 μL *per* tumor in PBS 100 mM, pH 7.5) administered on alternate days for 1 week. On day 6 after the start of therapy, mice were euthanized via CO_2_ atmosphere, and tumors and major organs were collected for histopathological evaluation.

Immunocompetent tumor model: treatments started on day 9 following B16–F10 cell inoculation surgery (set as day 0), once tumors were palpable (approximately 30 mm^3^). The experimental groups were the untreated control, NM­(dox)-LOx, NM-LOx, and NM­(dox)­LOx-i, (n = 11). The nanodevices were administered intraperitoneally, three times over 1 week, at a dose of 20 mg kg^–1^ (100 μL *per* injection in PBS 100 mM, pH 7.5). On day 5 post-treatment initiation, animals were sacrificed by cervical dislocation, and blood, tumors, and major organs were harvested for subsequent analyses.

Toxicological evaluation: NM-LOx was administered to healthy C57BL/6 mice (n = 6) by intraperitoneal injection at a single dose of 60 mg kg^–1^ (100 μL per injection in PBS 100 mM, pH 7.5). Untreated mice were in the study as a control (n = 6). On day 15 post-treatment, animals were euthanized by cervical dislocation, and blood and major organs were collected for further analyses.

Highlight that the body weight and welfare of the animals were evaluated during all the experimental procedures according to the criteria of the Morton and Griffiths scale.

#### 4.4.4 Lactate Measurement in Tumor

Tumor lactate was determined by a standard colorimetric kit (MAK329) following the manufacture instructions and by NMR. For NMR, tumor tissues underwent pulverization in liquid nitrogen, perchloric acid treatment and freeze-drying to extract metabolites. Lyophilized samples were resuspended in 2 mL of D_2_O and centrifuged at 10,000 rpm for 5 min. 450 μL of the supernatant were transferred to a 5 mm NMR tube and 50 μL of D_2_O with deuterated 3-(trimethylsilyl)-1-propanesulfonic acid sodium salt (DSS-*d*_6_) 1 mM were added to the NMR tube, providing a final concentration of DSS-*d*_6_ of 0.1 mM as an internal standard for chemical shift and concentration referencing.

^1^H NMR spectroscopy experiments were performed in a 600 MHz spectrometer (Bruker Avance DRX 600) equipped with a triple resonance probe (^1^H/^13^C/^31^P) (Bruker GmbH, Rheinstetten, Germany) at U26 NMR: Biomedical Applications II platform from Nanbiosis (Research Infrastructures & Services of CIBER-BBN). Noesy sequence was used for the acquisition of the 1D ^1^H NMR spectra at 300 K with 128 scans and a spectral width of 14 ppm. Afterward, spectra underwent phase, baseline and chemical shift correction with TopSpin 4.1.3 software (Bruker BioSpin Corporation). The quantitation was carried out using Chenomx NMR suite V12.0 (Chenomx Inc.), with automatic phase and baseline correction (Whittaker Spline). Lactate was fitted and concentration regards DSS was calculated.^48,49^

#### 4.4.5 TEM Analysis of Tumor Sections

For the TEM imaging of tumors obtained for the immunodeficient model, tumors were fixed with 2% PFA for 24 h and then washed twice with 100 mM PBS, pH 7.4. Then, tumors were cut with a Leica vibratome into rectangular sections of 1 mm^3^ (n = 4). These pieces were subjected to dehydration using ethanol. To finish, samples were stained with 1% uranyl acetate and 1% osmium tetroxide, included in Araldite epoxy resin, and sectioned into 50–70 nm nanoslices.

#### 4.4.6 Histology Processing

Tumor tissues from both models were embedded in OCT compound, rapidly frozen on dry ice, and cut into 10 μm sections using a cryostat. Sections were mounted on glass slides and processed following the corresponding specific protocol in each trial. Subsequently, nuclei were counterstained with Hoechst 33342 under light-protected conditions to preserve fluorescence integrity. Mice’s organs from tumor-bearing and healthy C57BL/6 mice, and some tumors from both immunocompetent and the immunocompromised models (n = 4) were fixed using 4% PFA, embedded in paraffin, and cut in 5 μm sections for processing using the hematoxylin and eosin staining technique.

#### 4.4.7 Evaluation of Doxorubicin in Tumor Sections

CLSM was employed to record images of the tumor sections showing the release of doxorubicin (λ_ex_ = 488 nm, λ_em_ = 555 nm) of both models (immunosuppressed and immunocompetent) using 20× and 40× objectives. Mean fluorescence intensity corresponding to doxorubicin was quantified with ImageJ software and analyzed with GraphPad Prism 9 program (*n* ≥ 4).

#### 4.4.8 Detection of Apoptosis in Tumor Sections

To investigate the pro-apoptotic effects induced by the different nanoparticle formulations, a TUNEL staining was conducted. Sections were mounted on glass slides, subsequently fixed, permeabilized, and processed according to the In Situ Cell Death Detection Kit (Merck) protocol. CLSM was employed to image the samples (λ_ex_ = 488 nm, λ_em_ = 550 nm) using 20× and 40× objectives. Whole-section scans were performed with an ImageJ software, and apoptotic cells were quantified and analyzed with GraphPad Prism 9 program (*n* ≥ 4).

#### 4.4.9 Hypoxia Measurement in Tumor Sections

Hypoxic regions within tumor tissues in the immunosuppressed model were identified using the EF5 Hypoxia Detection Kit (Alexa Fluor 488, Merck Millipore). Tumor sections were blocked 1 h with 100 mM PBS, pH 7.4, with 5% FBS to minimize nonspecific binding and washed with PBS + 0.3% Tween. Then tumor slides were incubated with the anti-EF5 monoclonal antibody (clone ELK3–51) conjugated to Alexa Fluor 488 (diluted to a working concentration of 75 μg mL^–1^) overnight at 4 °C of temperature in a humid chamber. After washing with PBS, sections were mounted using a fluorescence-compatible mounting medium and covered with glass coverslips.

Fluorescent imaging was performed using a fluorescence microscope equipped with filters appropriate for Alexa Fluor 488. Control sections included EF5-untreated tissue, sections processed without primary antibody, and positive controls consisting of tissues previously validated as hypoxic. Images were obtained by CLSM (λ_ex_ = 488 nm, λ_em_ = 520 nm) at the 20×, 40×, and 63× magnifications. Hypoxic tumor regions were measured by scanning the sections with an ImageJ software and analyzed with GraphPad Prism 9 program (*n* ≥ 4).

#### 4.4.10 Immunophenotyping of T-Cell Subsets (CD4+, CD8+, and CD3+) in Fresh Tumor Tissue

To study the lymphocyte infiltration, the tumors from the immunocompetent model were excised immediately after the mice were sacrificed. Tumors were mechanically minced into small fragments (∼1–2 mm) using sterile scissors and scalpels. Minced tissues were digested in 2 mL of a digestion buffer (DMEM supplemented with HEPES, inactivated FBS, collagenase, and DNase I) at 37 °C with constant agitation for 1 h. The digested tissue was passed through a 70 μm cell strainer to obtain a single-cell suspension. Cells were centrifuged at 400 × g for 5 min and resuspended in 3 mL of cold red blood cell (RBC) lysis (00–4300–54, Invitrogen) buffer for 5 min on ice. The reaction was stopped by adding complete medium, and cells were centrifuged again at 400 × g for 7 min. The resulting pellet was resuspended in PBS supplemented with 2% FBS. Cell counts were performed using a hemocytometer, and aliquots of 1.5 × 10^6^ cells were prepared for staining. Then, cells were washed and stained with Zombie Violet (BioLegend, 423114) viability dye at room temperature for 20 min in the dark. Subsequently, cells were fixed in intracellular fixation buffer for 20 min at room temperature in the dark. For flow cytometric analysis, cells were incubated with surface antibody panels in PBS + 2% FBS. The panel for general lymphocyte immunophenotyping included CD45-APC (Invitrogen, 17–0451–82), CD3-Cy7 (BD Biosciences, 560590), CD8-PE (Invitrogen, MA1–10304), and CD4-Alexa Fluor 488 (Invitrogen, 53–0041–82). All samples were finally resuspended in PBS and analyzed by flow cytometry. Appropriate fluorescence-minus-one and unstained controls were included for gating and compensation.

#### 4.4.11 Immunofluorescence Staining of CD8+ T-Cells in Tumor Sections

Tumor cryosections from the immunocompetent model were thawed for 15 min at room temperature and fixed in prechilled acetone (−20 °C) for 10 min, followed by air drying for 10 min. Sections were rehydrated in PBS for 5 min and blocked with 10% goat serum in PBS for 60 min. Slides were incubated with rat antimouse CD8 (Invitrogen, 14–0081–85) primary antibody (1:50 in 1% goat serum/PBS) for 24 h at room temperature, washed twice in PBS (5 min each), and subsequently incubated with Alexa Fluor 488–conjugated goat antirat secondary (Invitrogen, A48262TR) antibody (1:300 in 1% goat serum/PBS) for 1 h. After two additional PBS washes, nuclei were counterstained with DAPI. Sections were mounted using Mowiol. Samples were stored at 4 °C until imaging by CLSM (λ_ex_ = 488 nm, λ_em_ = 520 nm, magnifications 20x and 40x).

#### 4.4.12 Biodistribution of Platinum

Organs from healthy C57BL/6 mice treated with NM-LOx (n = 5) and organs from control mice (n = 3) were weighed and digested in a microwave oven to study the biodistribution of Pt by ICP-MS. Data are expressed as mg Pt per kg tissue.

### 4.5 Statistical Analysis

Data was analyzed using GraphPad Prism 9 software. Group comparisons were performed employing one-way or two-way ANOVA, followed by Tukey’s multiple comparison posttest. Statistical significance was defined as *p* < 0.05, with the following notation: *p* < 0.05 (*), *p* < 0.01 (**), *p* < 0.001 (***), and *p* < 0.0001 (****).

## Supplementary Material



## Notes

Chemicals, instruments, and techniques, description of prepared nanoparticles (Table S1), PXRD patterns, N_2_ adsorption–desorption isotherms (Table 2), FTIR, amount of LOx attached in nanomotors by BCA (Table 3), hydrodynamic diameter, z-potential, analysis of batch-to-batch reproducibility (Table 4), cargo-controlled delivery in response to proteases, DMSO (Table 5) and lysosomal components, lactate in SK-MEL-103 cells and other cancer cell lines, LOx activity assay, H_2_O_2_-reducting activity test, nanomotors’ trajectories, motion characterization of NM­(dox)-LOx-i, ROS generation, cell internalization by CLSM and TEM, tumor penetration of nanomotors by CLSM (3D reconstruction) and quantification, cytotoxicity profile of SK-MEL-103 cells treated with nanodevices, IC_50_ values in SK-MEL-103, cytotoxicity profile of B16–F10 spheroids treated with nanodevices, IC_50_ values in B16–F10; body weight monitoring, tumor volume at the beginning and end of the experiments, representative image of nanomotors’ in tumor by TEM, H&E analysis of tumors, extracellular staining of tumor-infiltrating lymphocytes, biosafety analysis of blood samples, H&E analysis of major organs, and nanoparticles biodistribution by ICP-MS (PDF)
