# Supplementary material for: Overcoming Barriers in Solid Tumors: Lactate-Fueled Nanomotors Enhance Penetration and Reverse Immunosuppression
Source: J Am Chem Soc. 2026 Aug 11;148(40):42949–68. doi: 10.1021/jacs.6c05546 (PMC13674273; doi:10.1021/jacs.6c05546)
Supplement: Supplementary file 1 [file ja6c05546_si_001.pdf]

Supporting Information

Overcoming Barriers in Solid Tumors: Lactate-Fueled Nanomotors Enhance Penetration and Reverse Immunosuppression

Juan José Esteve-Moreno,[1,2,3] Andrea Escudero,[1,2,3] Francisco J. Hicke,[1,3,4] Víctor Sanz-Álvarez,[1,3,4] María Miralles-Buleo,[1,2,4] María Dolores Marcos,[1,3,5] M. Carmen Martínez-Bisbal,[1,3,4,6] Alba García-Fernández,[1,2,3,4,*] Paula Díez[1,3,4,*] and Ramón Martínez-Máñez[1,2,3,4,5,*]

[1] Instituto Interuniversitario de Investigación de Reconocimiento Molecular y Desarrollo Tecnológico (IDM) Universitat Politècnica de València, Universitat de València. Camí de Vera, s/n. 46022, València, Spain.

[2] Unidad Mixta UPV-CIPF de Investigación en Mecanismos de Enfermedades y Nanomedicina, Universitat Politècnica de València, Centro de Investigación Príncipe Felipe. C/ Eduardo Primo Yúfera, 3. 46012, València, Spain.

[3] CIBER de Bioingeniería, Biomateriales y Nanomedicina (CIBER-BBN), Instituto de Salud Carlos III. 28029, Madrid, Spain.

[4] Unidad Mixta de Investigación en Nanomedicina y Sensores. Universitat Politècnica de València, Instituto de Investigación Sanitaria La Fe (IIS La Fe). Avenida Fernando Abril Martorell 106, Torre A. 46026, València, Spain.

[5] Departamento de Química, Universitat Politècnica de València. Camí de Vera, s/n. 46022, València, Spain.

[6] Departamento de Química-Física, Universitat de València. Burjassot. 46100, València, Spain.

***Chemicals**: n*-cetyltrimethylammonium bromide (CTABr), tetraethyl orthosilicate (TEOS), sodium hydroxide (NaOH), polyvinylpyrrolidone (PVP), chloroplatinic acid hydrate (H_2PtCl_6), ascorbic acid, paraffin, 3-(mercaptopropyl)trimethoxysilane (MPS), chloroform, 3-aminopropyltriethoxysilane (APTES), N-(3-dimethylaminopropyl)-N'-ethylcarbodiimide hydrochloride (EDC), N-hydroxysuccinimide (NHS), 2,2'-Azino-bis(3-ethylbenzthiazoline-6-sulfonic acid) (ABTS) and doxorubicin hydrochloride (Dox)

were provided from Aldrich. Ethanol and methanol were purchased from Scharlab. Acetonitrile (ACN), Dulbecco´s Modified Eagle Medium (DMEM), Fetal Bovine Serum (FBS), Dulbecco´s Phosphate Buffer Saline (PBS), peroxidase from horseradish (HRP), Bovine Serum Albumina (BSA), protease from *Streptomyces griseus*, Hoechst 33342, trypsin and cell proliferation reagent WST-1 were purchased from Sigma-Aldrich (Merck). Native Microorganism Lactate oxidase (LOx) was obtained from Genochem.

Instruments and techniques

Power X-ray diffraction (PXRD) was performed on a D8 Advance Seifert 3000TT diffractometer employing CuKα radiation at low angles ($1.5 < 2\theta > 7$, with steps of 0.04 degrees and 3s for step) and high angles ($35 < 2\theta > 80$ with steps of 0.04 degrees and 1 s for step). Dynamic light scattering (DLS): a ZetaSizer Nano ZS (Malvern) was used for DLS and zeta potential analysis. Transmission electron microscopy (TEM) images were acquired on a JEOL TEM-2100F electron microscope and scanning transmission electron microscopy coupled with electronic energy dispersive x-ray spectroscopy (STEM-EDX) on a JEM 2100F instrument. N_2 adsorption-desorption isotherms were measured using a Micromeritics TriStar II Plus automated analyzer, which degassed the nanoparticles overnight at 90 or 120 °C. Using the Brunauer-Emmett-Teller (BET) model and adsorption data in the low-pressure region, the specific surface area was calculated. The Barrett-Joyner-Halenda (BJH) model was used to determine the size and volume of the pores. A Tensor 27 (Bruker) equipment was used to perform Fourier transformed infrared spectroscopy (FTIR). Nanosight NS300 (Malvern) was used to analyze motion of nanomotors. A JASCO V-650 spectrophotometer was employed to measure the absorbance of activity assays. A JASCO FP-8500 fluorimeter was used to analyze fluorescence emissions in drug delivery assays. A Leica TCS SP8 AOBS inverted microscope was used to obtain confocal laser scanning microscopy (CLSM) images, and a Leica Aperio Versa digital pathology research scanner was used to obtain tissue fluorescent images. A digital camera and an FEI Tecnai Spirit G2 microscope operating at 80 kV were used to capture TEM images of cells and tissues. Cytotoxicity studies were carried out in a Wallac Victor2TM spectrophotometer and flow cytometry assays were performed in a CytoFLEX S instrument.

1. Characterization of nanoparticles

Table S1. Summary of the nanoparticles synthetized in this study.

Nanodevice	Illustration	Description and function
**MSN**	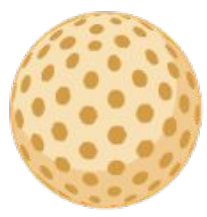	Mesoporous silica nanoparticles
**NM**	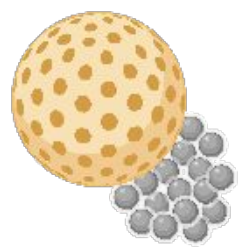	*Snowman*-like Janus nanoparticles of PtNPs and MSN
**NM(dox)-LOx**	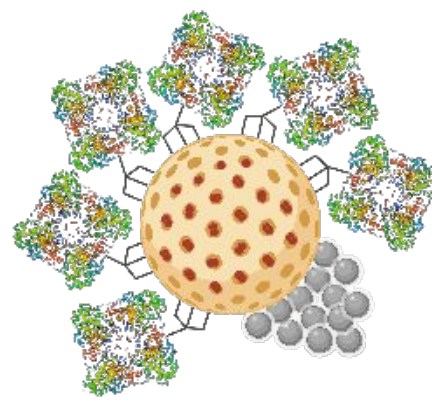	Janus Pt-MSN loaded with Dox and capped with LOx
**NM-LOx**	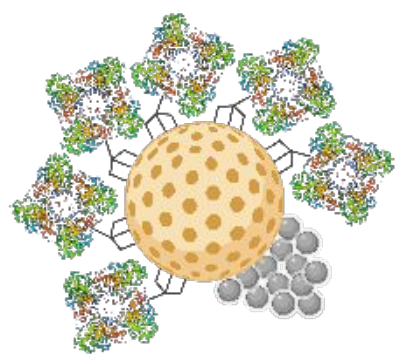	JanusPt-MSN capped with LOx (not loaded with Dox)
**DA**	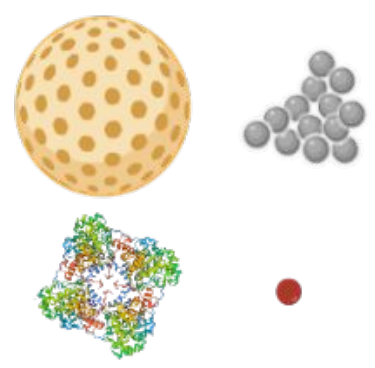	Unassembled components of the nanomotor
**NM(dox)-LOx-i**	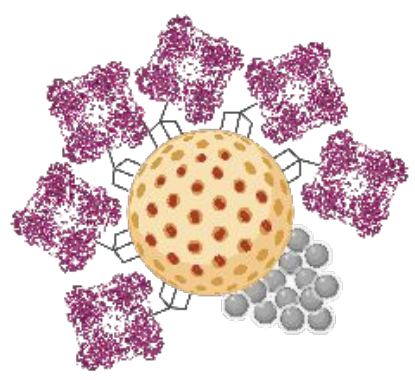	Janus Pt-MSN loaded with Dox and capped with inactivated LOx by heat
**NM*-LOx**	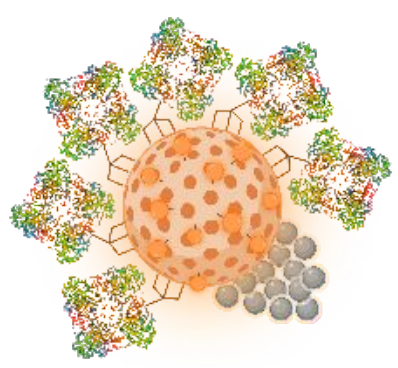	Labelled nanomotor capped with LOx
**NM*-LOx-i**	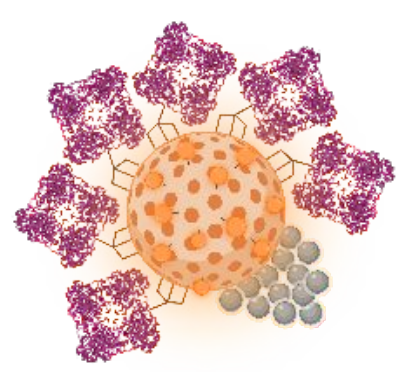	Labelled nanoparticle capped with inactive LOx

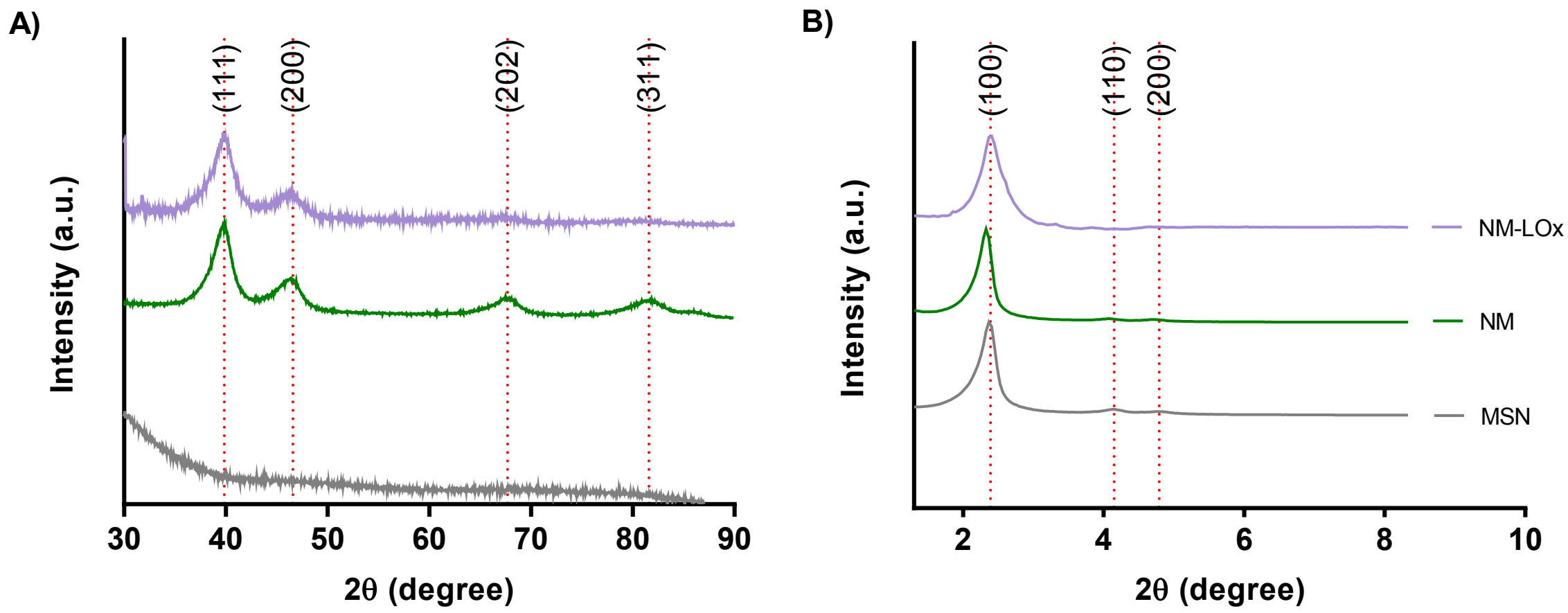

Figure S1. PXRD patterns of calcined MSNs (grey), NM (dark green) and NM-LOx (purple) at high ($30 < 2\theta > 90$) (A) and low ($1.5 < 2\theta > 10$) (B) angles. The standard theoretical reference lines for platinum and MCM-41 are shown in red. For the platinum phase, we used the reference lines from JCPDS card No. 04-0802, corresponding to face-centered cubic (fcc) Pt. For the MCM-41 phase, which does not have a specific JCPDS card, we calculated the reference peak positions based on the hexagonal lattice parameters derived from experimental d-spacings of MSN of the (100), (110) and (200) reflections characteristic of a well-ordered MCM-41 structure.

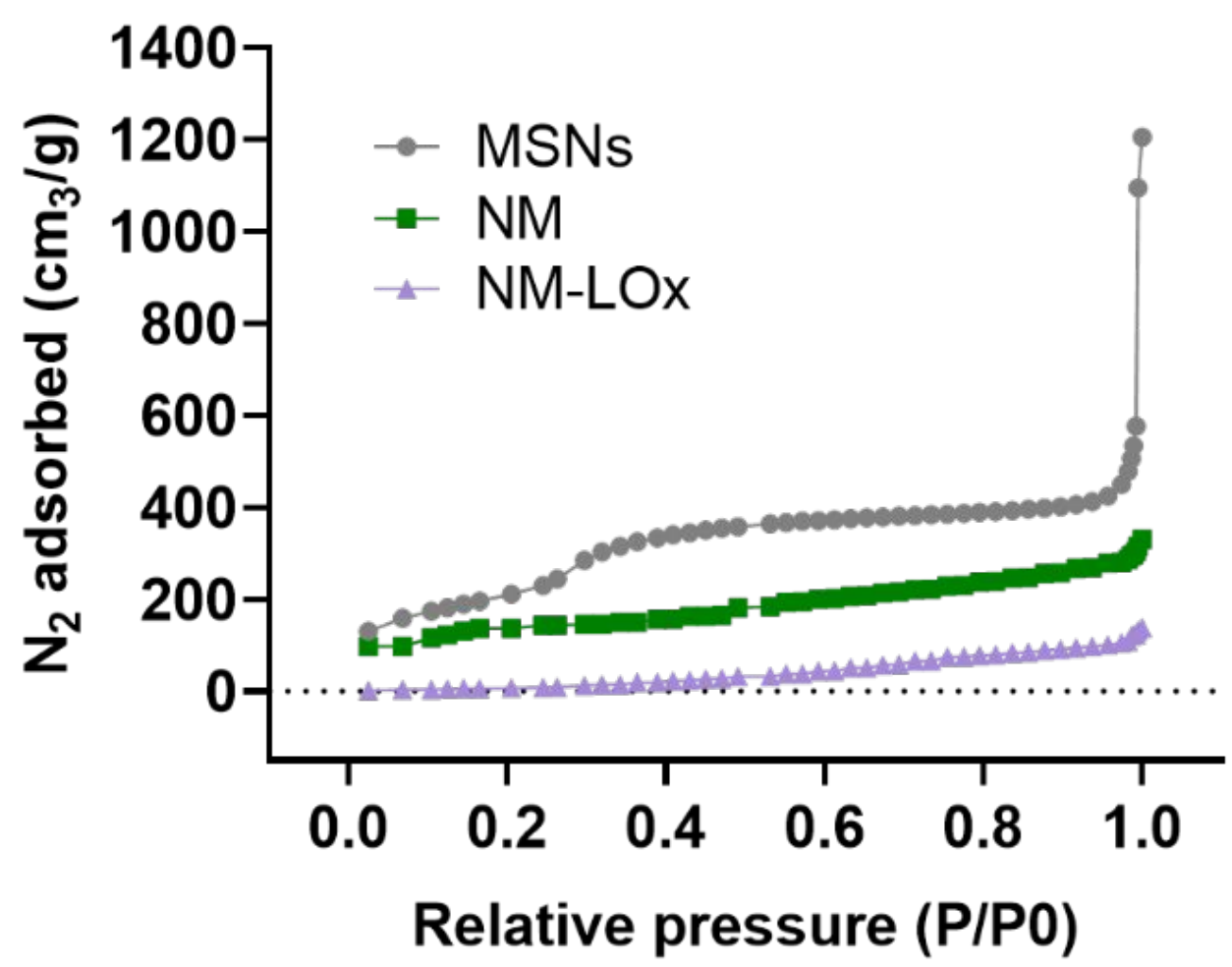

Figure S2. N_2 adsorption-desorption isotherms of calcined MSNs (grey), NM (dark green) and NM-LOx (purple).

Table S2. Pore volume, pore size and specific surface of MSN, NM and NM-LOx calculated from the adsorption-desorption isotherms and applying the BJH and BET models.

Nanoparticle	Pore volume (BJH) ($cm^3\ g^{-1}$)	Pore size (BJH) (nm)	Specific surface (BET) ($m^2\ g^{-1}$)
MSNs	0.98	2.69	1329

NM	0.32	2.49	633
**NM-LOx**	**0.06**	**-**	**70**

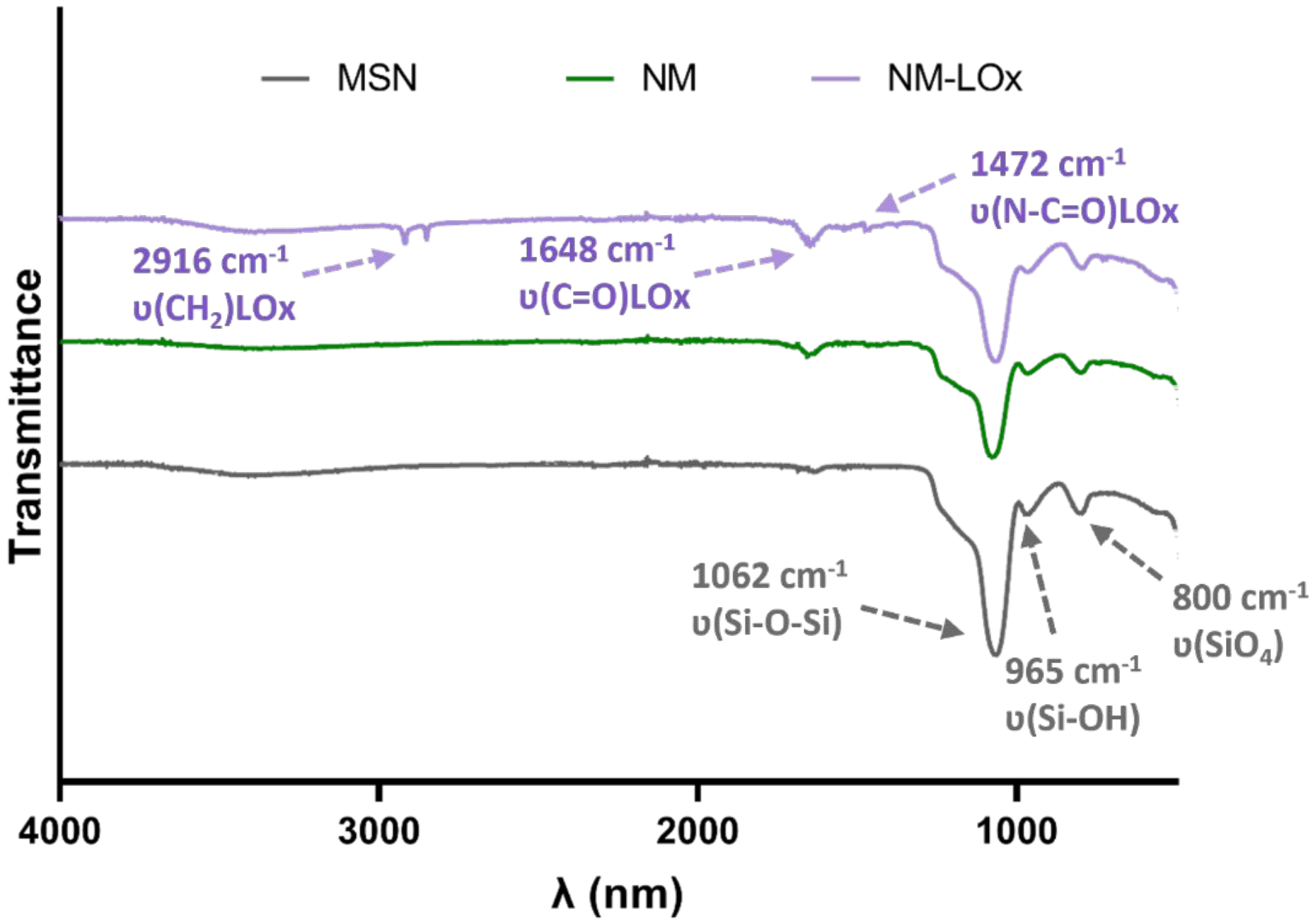

Figure S3. FTIR spectrum of calcined MSNs (grey), NM (green) and NM-LOx (purple).

Table S3. Determination of the amount of LOx attached in the nanoparticles by BCA.

Nanoparticle	BCA (µg mg NP $^{-1}$)
NM-LOx	314
**NM(dox)-LOx**	**356**

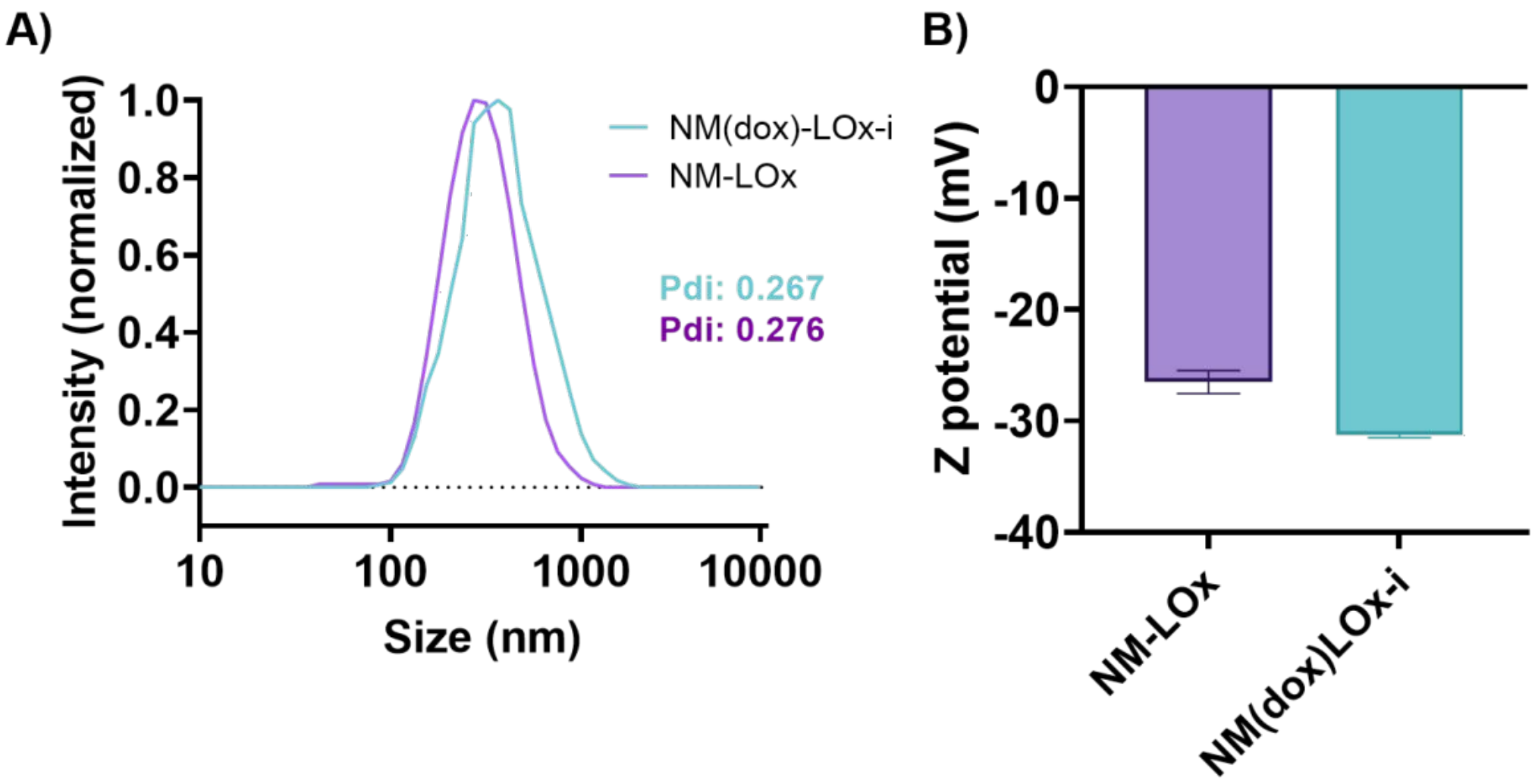

Figure S4. Hydrodynamic diameter (A) and z-potential (B) of control nanoparticles (purple: NM-LOx; blue: NM(dox)-LOx-i), determined by DLS. Data represent mean ± SEM, n = 3.

Control nanoparticles, both the unloaded (NM-LOx) and the inactive nanomotor (NM(dox)-LOx-i), show sizes and z- potential values comparable to those of the complete nanomotor. DLS measurements showed hydrodynamic diameters of 377.8 ± 11.4 nm for NM(dox)-LOx-i and 318 ± 7 nm for NM-LOx, respectively. Both control nanoparticles exhibited negative ζ-potential values, NM-LOx (purple), - 25.6 ± 2.7 mV and NM(dox)-LOx-i, -31.3 ± 0.5 mV.

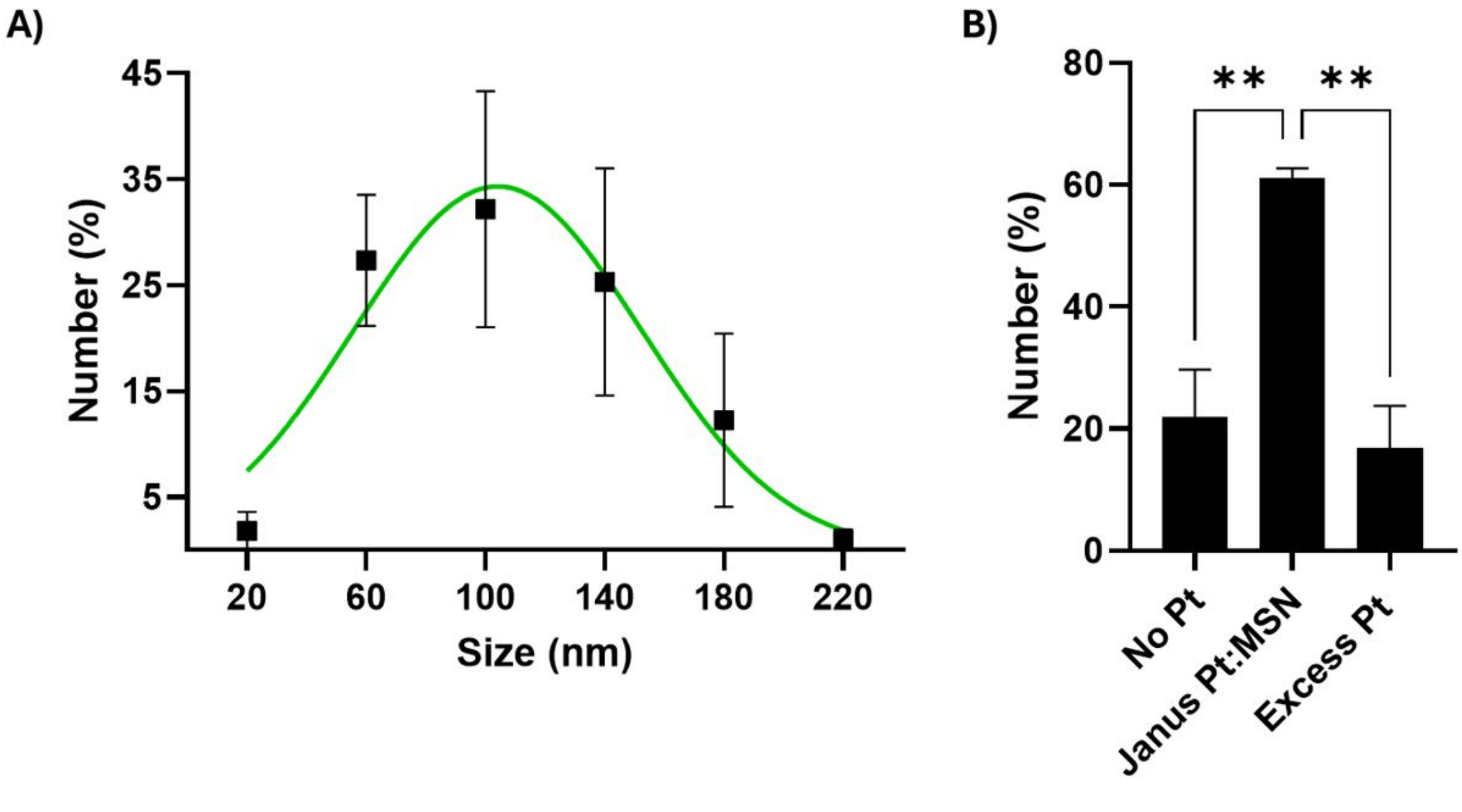

Figure S5. TEM analysis of the NM batch-to-batch reproducibility, prepared from three independent Pickering emulsions. (A) Size distribution analysis, measuring the dimensions of at least 18 nanoparticles of each synthesis. The mean size of NM was determined to be 128 ± 28 nm. (B) Efficiency of the synthesis process, quantifying the

MSN without Pt, Janus Pt-MSN morphology and MSN with an excess of Pt from at least 39 nanoparticles of each synthesis. The optimal MSN:Pt ratio was observed in 61.2 ± 2.6 % of the particles analyzed. Data is presented as mean ± SEM, n = 3. Statistical significance was assessed via one-way ANOVA (** $p < 0.01$).

Table S4. Batch-to-batch reproducibility of NM(dox)-LOx synthesis prepared from three independent Pickering emulsions (S1, S2 and S3). Data represents mean ± SD, n = 3.

Janus Pt-MSN batch	Hydrodynamic diameter (nm)	Z- Potential (mV)	Activity (U mg^{-1})	Doxo (µg Doxo/mg NP)
NM(dox)-LOx, S1	392.5	-24.4	34.9	117.3
NM(dox)-LOx, S2	409.5	-27.4	30.9	107.0
NM(dox)-LOx, S3	427.1	-29.9	35.4	92.4
**Mean ± SD**	**409.7 ± 17.3**	**−27.2 ± 2.8**	**33.7 ± 2.5**	**105.6 ± 12.5**

2. Nanomotor capability to deliver doxorubicin in a controlled manner

Table S5. Quantification of Dox release from NM(dox)-LOx using fluorescence spectroscopy. Data represents mean ± SD, n = 3.

Doxorubicin Release	Protease-responsive release	Forced-Release (DMSO)
**Dox (µg mg NM^{-1})**	72.3 ± 14.3	105.6 ± 12.5
**% Release**	67.8 ± 0.3	100

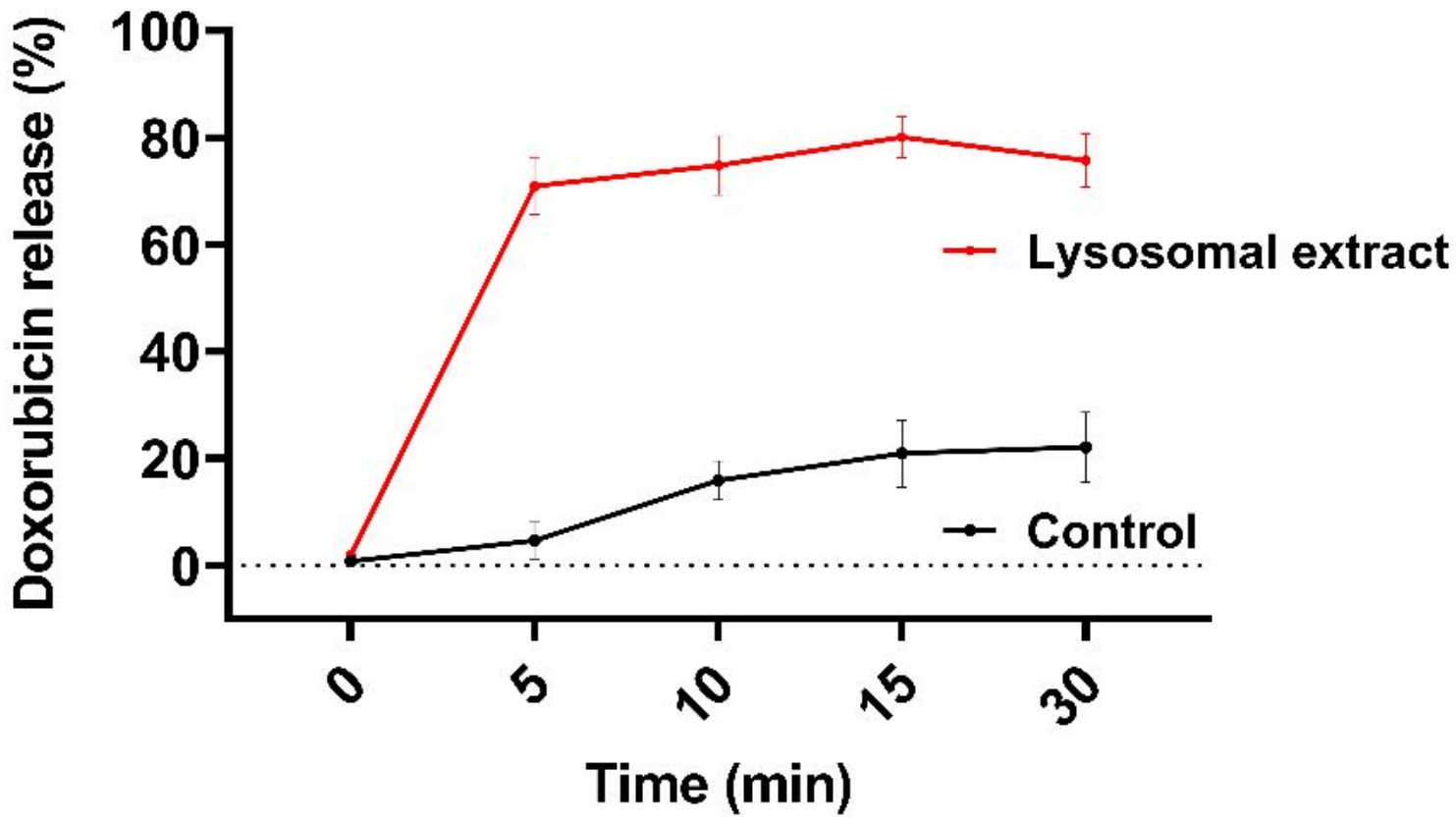

Figure S6. Normalized Dox release vs time (min) from NM(dox)-LOx monitored by fluorescence spectroscopy (λ_{em}, 555 nm; λ_{ex}, 470 nm) in presence of lysosomal extract (red) or PBS (black). Data represent mean ± SEM, n = 3.

3. Nanomotor functionalities in response to lactate: substrate depletion and propulsion

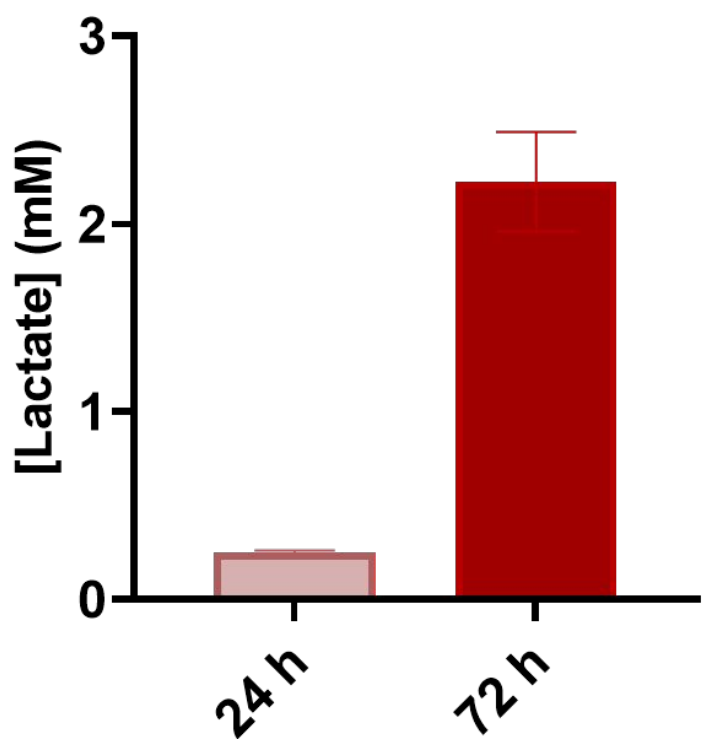

Figure S7. Lactate concentration in SK-MEL-103 cancer cell culture at 24 h (light red) and 72 h (dark red) measured with Lactate assay Kit (Sigma; MAK064). Soluble fractions of extra-cellular lactate were mixed with Master Reaction Mix and after 30 min of incubation, absorbance was measured at 570 nm. Data represents mean ± SD, n = 3.

Table S6. Lactate concentration in different cell lines at 24 h of growth. Data represents mean ± SD, n = 3.

Cell Line	[Lactate] (µg mL^{-1})	[Lactate] (mM)
WI-38	8.9 ± 1.4	0.07 ± 0.01
A549	15.8 ± 4.1	0.14 ± 0.04

**SK-MEL-103**	**28.1 ± 1.1**	**0.25 ± 0.01**

Table S7. Measurements of LOx activity by a spectrophotometric assay in selected nanoparticles and free LOx. Data represent mean ± SD, n ≥ 3.

**Nanoparticle**	**Activity ($U\ mg^{-1}$)**
NM*-LOx	24.8 ± 2.5
NM*-LOx-i	0.7 ± 0.1
NM-LOx	27.5 ± 1.5
NM(dox)-LOx-i	1.1 ± 0.9
Free LOx	121.1 ± 29.4
**NM(dox)-LOx**	**33.7 ± 2.5**

Table S8. Catalytic efficiency of LOx and Pt in NM-LOx, calculated under the experimental conditions used in the LOx and peroxidase-like activity assays.

**Catalytic parameters**	**LOx**	**Pt**
K_M (mM)	1	15.35
V_{max} ($M\ s^{-1}$)	4.5×10^{-7}	6.7×10^{-4}
k_{cat} (s^{-1})	0.07	1.5×10^{6}
**k_{cat}/K_M** ($M^{-1}\ s^{-1}$)	**70.2**	**9.9×10^{7}**

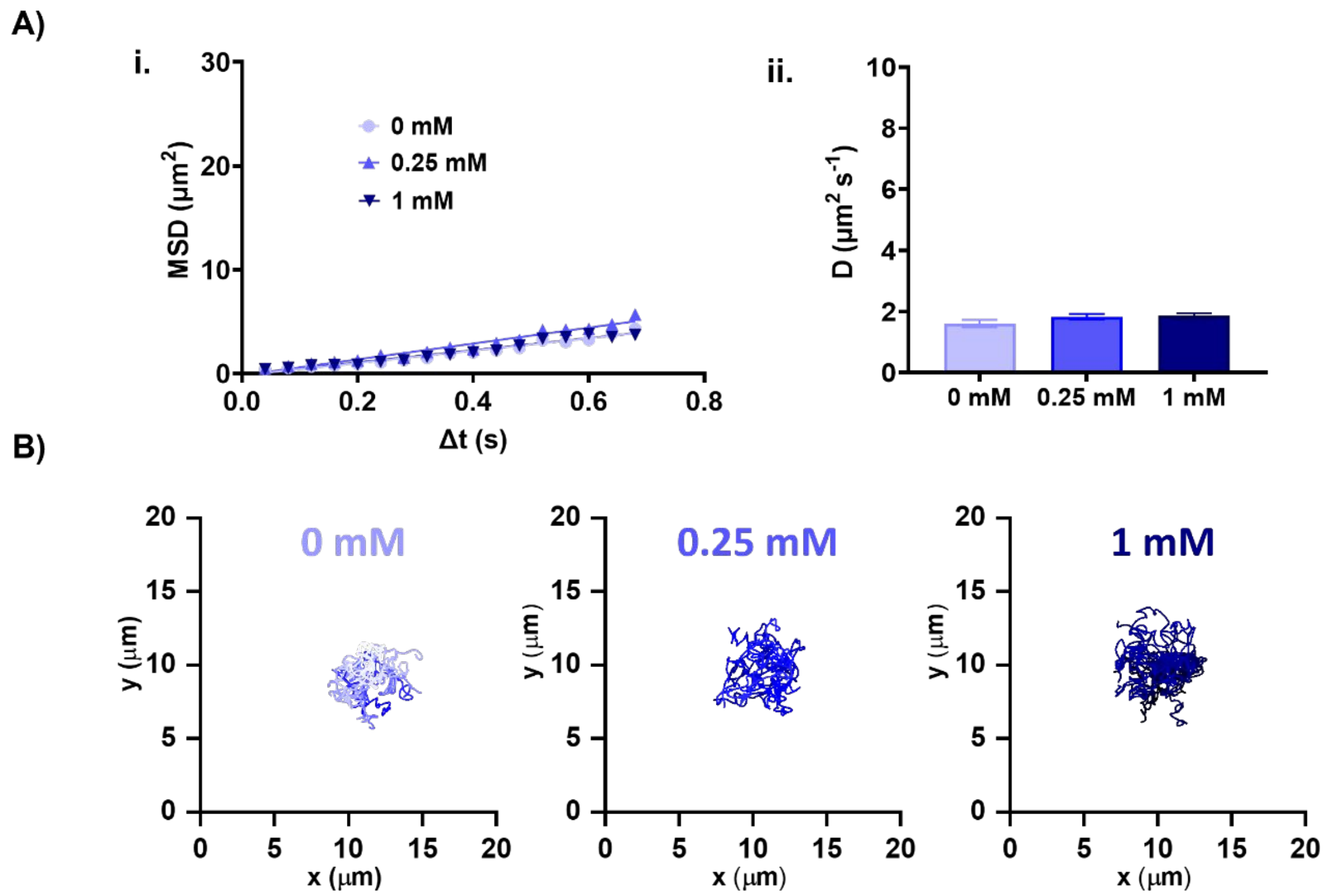

Figure S8. Evaluation of NM(dox)-LOx-i motion by NTA. (A-i) MSD vs Δt at different lactate concentrations (0 mM, light blue; 0.25 mM, blue 1 mM, dark blue), (A-ii) D_0 values calculated from MSD plots and (B-iii) representative spatial trajectories. Data are expressed as mean ± SEM for 25 nanodevices with sizes from 250 to 450 nm. Statistical significance was determined using one-way ANOVA. The differences were not statistically significant and, therefore, were not indicated.

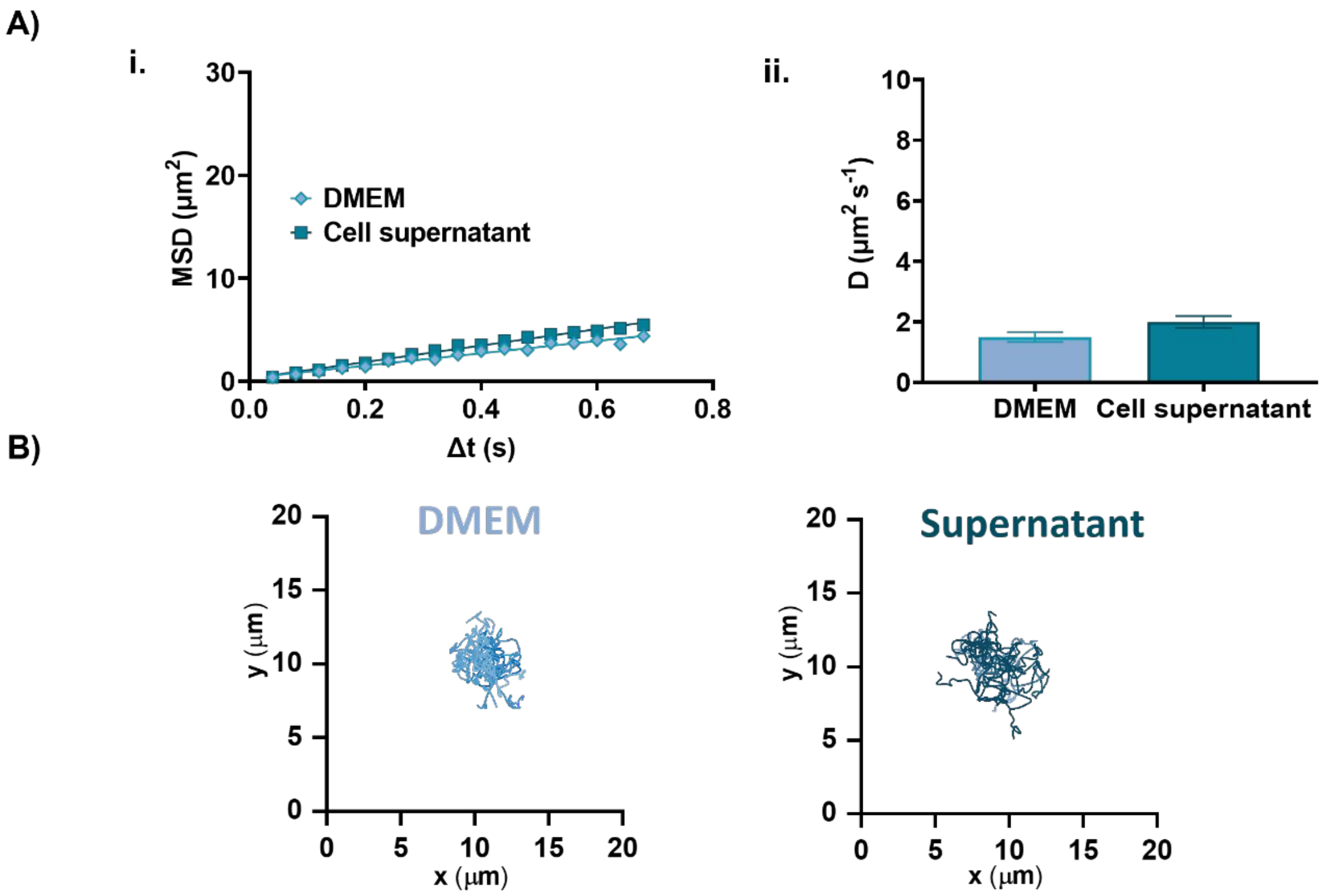

Figure S9. Evaluation of NM(dox)-LOx-i motion by NTA in biological media. (A-i) MSD vs Δt with DMEM (light turquoise) or with SK-MEL-103 cell supernatant (dark turquoise), (A-ii) D_0 values calculated from MSD plots and (B) representative spatial trajectories. Data are expressed as mean ± SEM for 25 nanodevices with sizes from 250 to 450 nm. Statistical significance was determined using one-way ANOVA. The differences were not statistically significant and, therefore, were not indicated.

4. **Nanomotor-mediated cytotoxicity, cellular uptake in 2D and tissue penetration in 3D melanoma models**

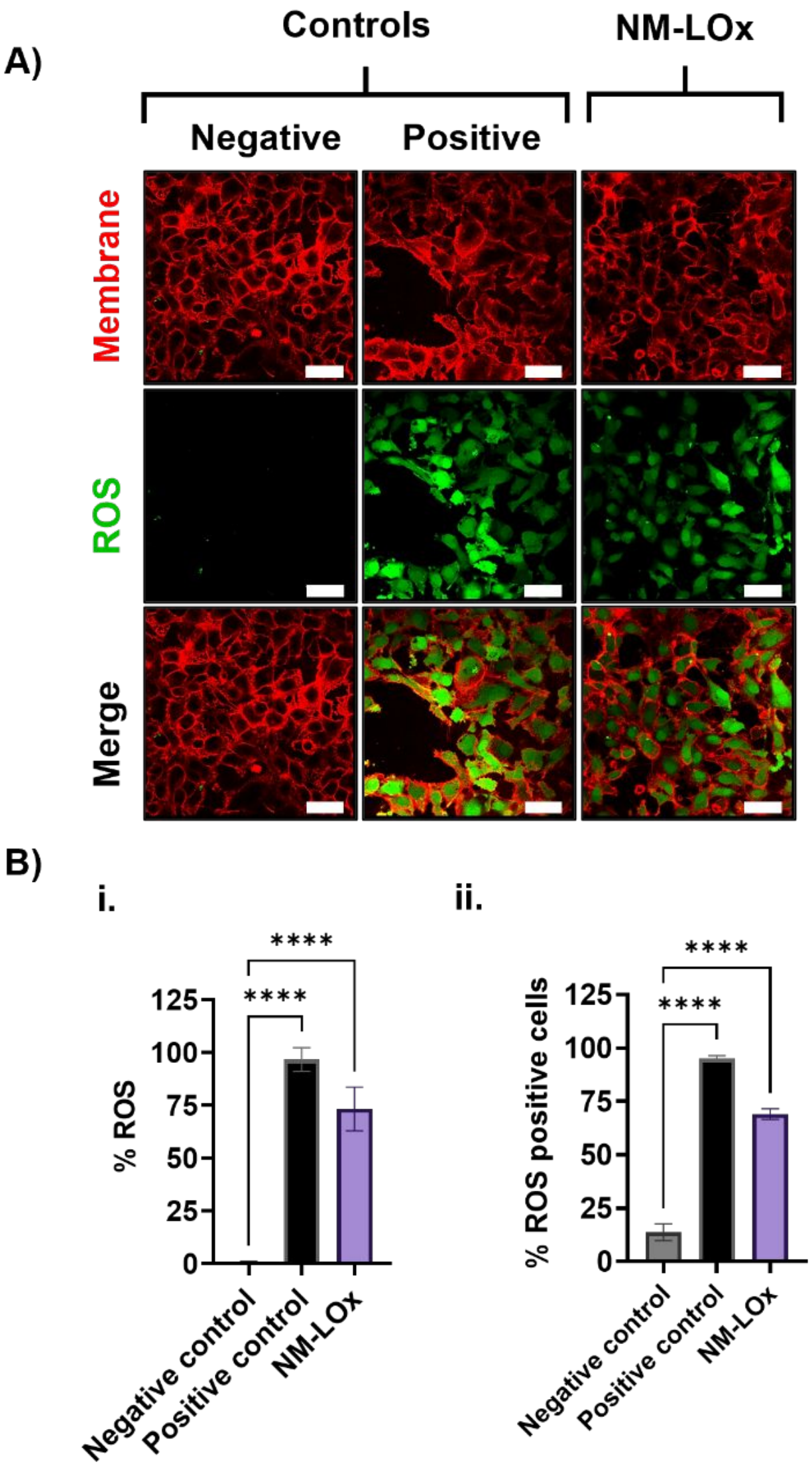

Figure S10. Intracellular ROS production by using DCFDA-H2DCFDA as detection probe. (A) SK-MEL-103 cells were treated with 50 µg mL^{-1} of NM-LOx and incubated for 30 min before visualization by CLSM (membrane, red; DCF oxidized by ROS, green). Cumene hydroperoxide (50 µM) was used as a positive control. Scale bar: 50 µm. (B-i) Quantification of fluorescence intensity of green emission that corresponds to ROS generation in CLSM images and (B-ii) quantification of the percentage of ROS-positive cells (green fluorescence) analysed by flow cytometry. Data are expressed as mean ± SEM, n = 6. Statistical significance was determined by a one-way ANOVA (* $p < 0.05$, ** $p < 0.01$, *** $p < 0.001$, **** $p < 0.0001$).

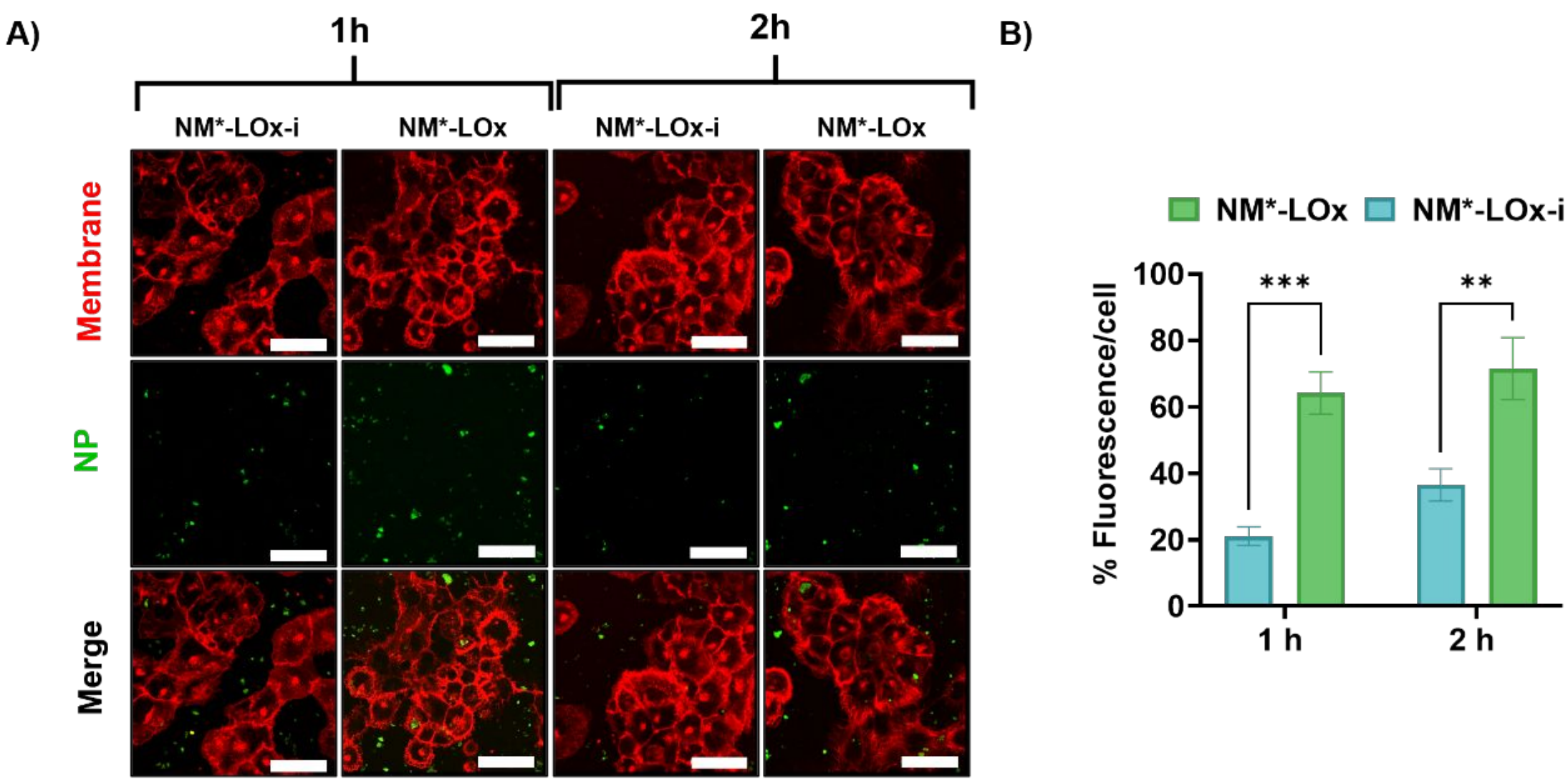

Figure S11. Comparison of NM*-LOx (labelled active nanomotor) and NM*-LOx-i (labelled inactive control) cell internalization in 2D cell culture. (A) CLSM images of SK-MEL-103 cells treated with both nanoparticles at 50 µg mL^{-1} and incubated for 1 h or 2 h (membrane, red fluorescence; nanomotors, green emission). (B) Subsequent quantification of fluorescence relative to nanoparticles within each cell using Image J (quantifying at least 12 images per group). Scale bar: 50 µm. Data are expressed as mean ± SEM, n = 4. Statistical significance was determined by a two-way ANOVA (* $p < 0.05$, ** $p < 0.01$, *** $p < 0.001$, **** $p < 0.0001$).

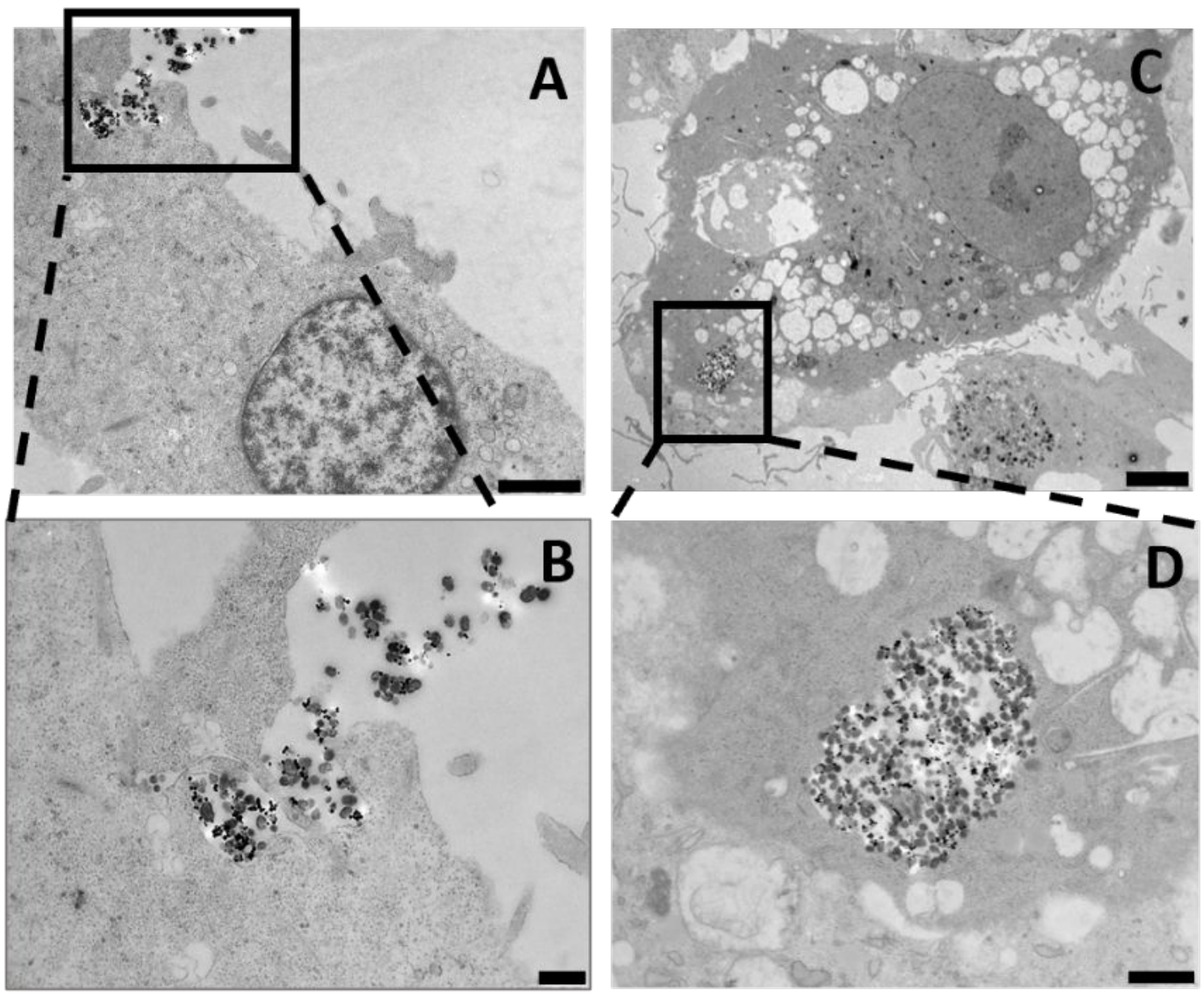

Figure S12. TEM images of SK-MEL-103 cells incubated with NM-LOx (50 µg mL^{-1}, 30 min) that are located within endocytic vesicles and in the perinuclear region, indicating active internalization. Scale bars: (A) 2 µm, (B) 500 nm, (C) 5 µm and (D) 1 µm.

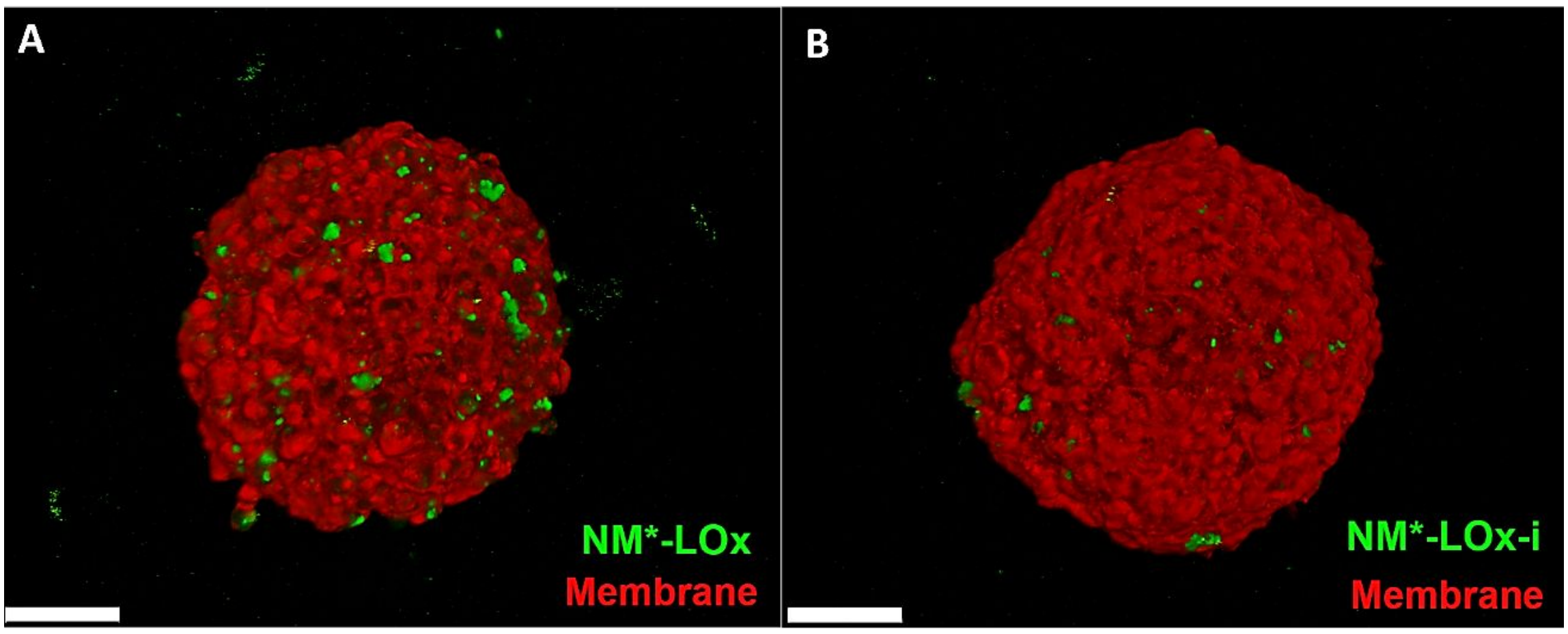

Figure S13. Three-dimensional reconstruction of a representative spheroid (red fluorescence, membrane) showing the penetration of each treatment, (A) NM*-LOx and (B) NM*-LOx-i (green fluorescence, nanoparticles). Analyzed by multi-photon confocal microscopy. Spheroids were treated with nanoparticles for 1 h at 50 µg mL^{-1}. Scale bars: 50 µm.

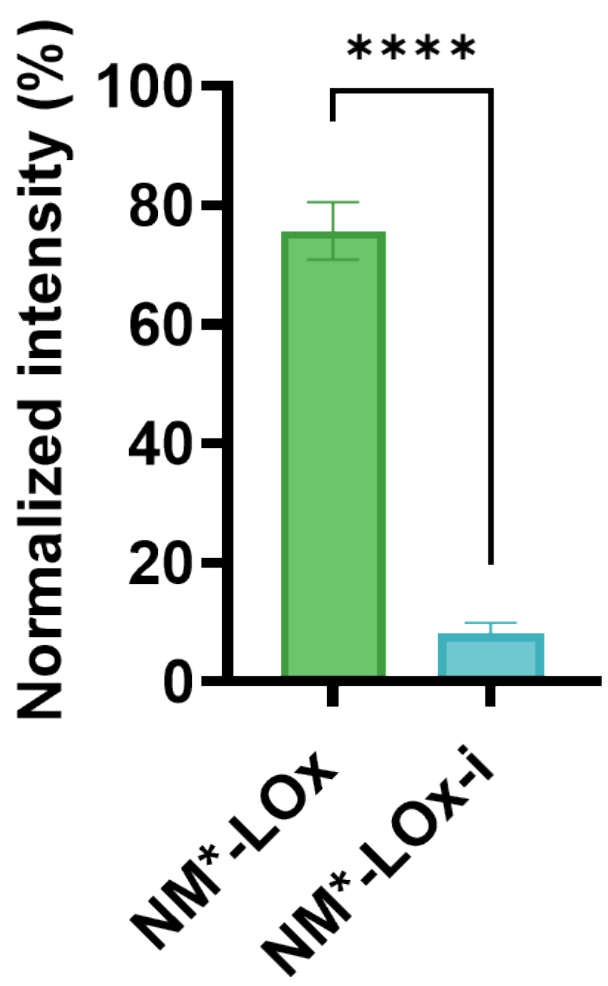

Figure S14. Quantification of fluorescence intensity of 3 randomly selected sections from 74 µm to 123 µm depth of the spheroid (sum of each section), corresponding to 48 % and 80 % of the longitudinal axis (from top to bottom). Data are presented as mean ± SEM, n = 3. Statistical significance was assessed using a two-way ANOVA Test (**** $p < 0.0001$).

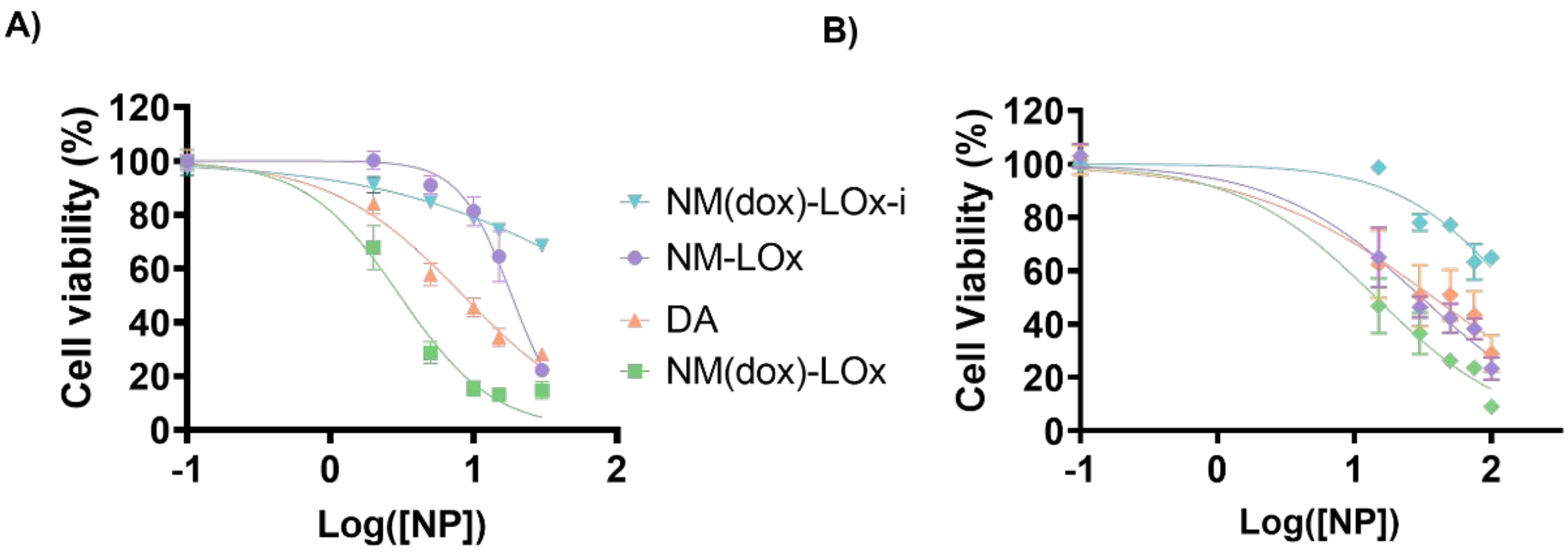

Figure S15. Cytotoxicity profile of SK-MEL-103 melanoma (A) cells and (B) spheroids treated with increasing concentrations (logarithmic scale) of the nanodevices: NM(dox)-LOx (green), NM-LOx (purple), NM(dox)-LOx-i (blue), and unassembled components (DA) (orange). Cell viability was determined after 24 h of incubation using the WST-1 assay. Data are expressed as mean ± SEM, n = 4. Dose-response curves were fitted by nonlinear regression to calculate IC_{50} values for each treatment (**Table S9**).

Table S9. Half maximal inhibitory concentration (IC_{50}) values of the different treatments against 2D SK-MEL-103 cells and 3D spheroids. NM(dox)-LOx displayed a significantly lower IC_{50} compared to NM-LOx, unassembled components (DA), and NM(dox)-LOx-i. Data represents IC_{50}, n = 4.

**IC_{50}** (µg mL^{-1})	**NM(dox)-LOx**	DA	NM-LOx	NM(dox)-LOx-i
2D SK-MEL-103	**3.0**	8.4	18.4	119.3
3D SK-MEL-103	**14.3**	37.6	30.9	155.4

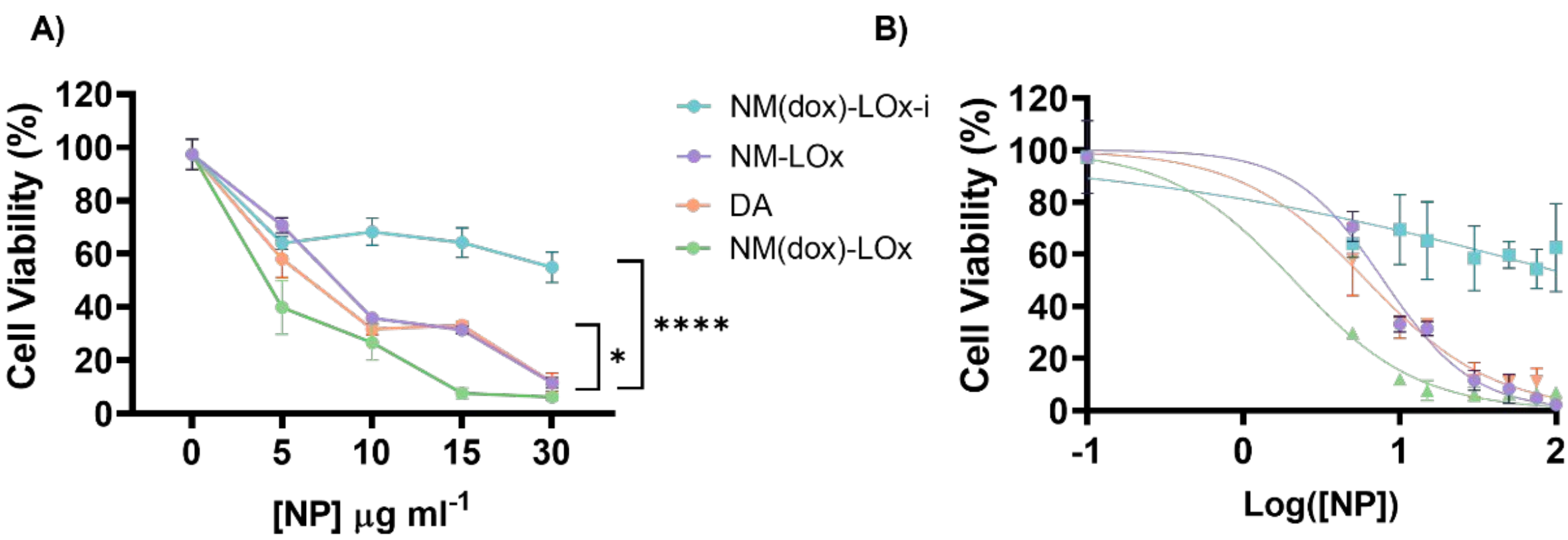

Figure S16. Effect of nanoparticles on the cell viability of B16-F10 murine melanoma spheroids. (A) Spheroids were treated with increasing concentrations (from 5 to

30 µg mL^{-1}) of NM(dox)-LOx (green), NM(dox)-LOx-i (blue), NM-LOx (purple), and unassembled components (DA) (orange) for 24 h. (B) Cytotoxicity profile of B16-F10 melanoma spheroids treated with increasing concentrations (logarithmic scale; from 5 to 100 µg mL^{-1}) of the indicated system. Cell viability was determined after 24 h of incubation using GloTitle assay. Data are expressed as mean ± SEM, n = 4. Statistical significance was determined by two-way ANOVA followed by Tukey's post hoc test (*p < 0.05, ****p < 0.0001). Dose-response curves were fitted by nonlinear regression to calculate IC_{50} values for each treatment (**Table S10**).

Table S10. Half maximal inhibitory concentration (IC_{50}) values of the different treatments against B16-F10 3D spheroids. NM(dox)-LOx displayed a significantly lower IC_{50} compared to NM-LOx, unassembled components (DA), and NM(dox)-LOx-i. Data represents IC50, n = 4.

**IC50** (µg mL^{-1})	**NM(dox)-LOx**	DA	NM-LOx	NM(dox)-LOx-i
3D B16-F10	**2.1**	6.1	7.9	164.0

5. Nanomotor anticancer activity in an *in vivo* human melanoma xenograft model

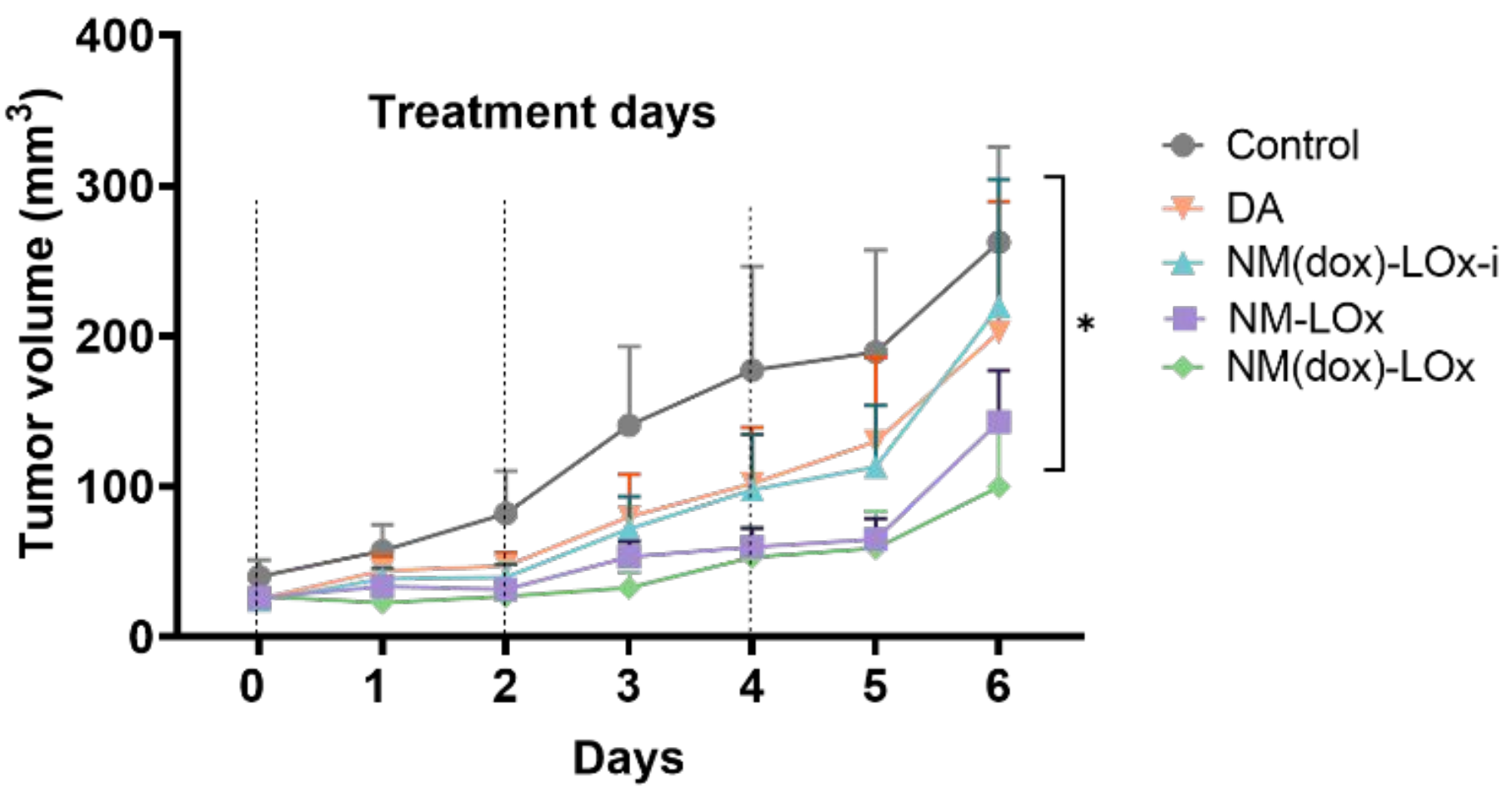

Figure S17. Monitorization of tumor volume after treatment with NM(dox)-LOx (green), NM-LOx (purple), NM(dox)-LOx-i (blue) or DA (orange), compared to control (gray). Data are expressed as mean ± SEM, n = 6. Statistical significance was determined by a two-way ANOVA, followed by Tukey's test (*p < 0.05).

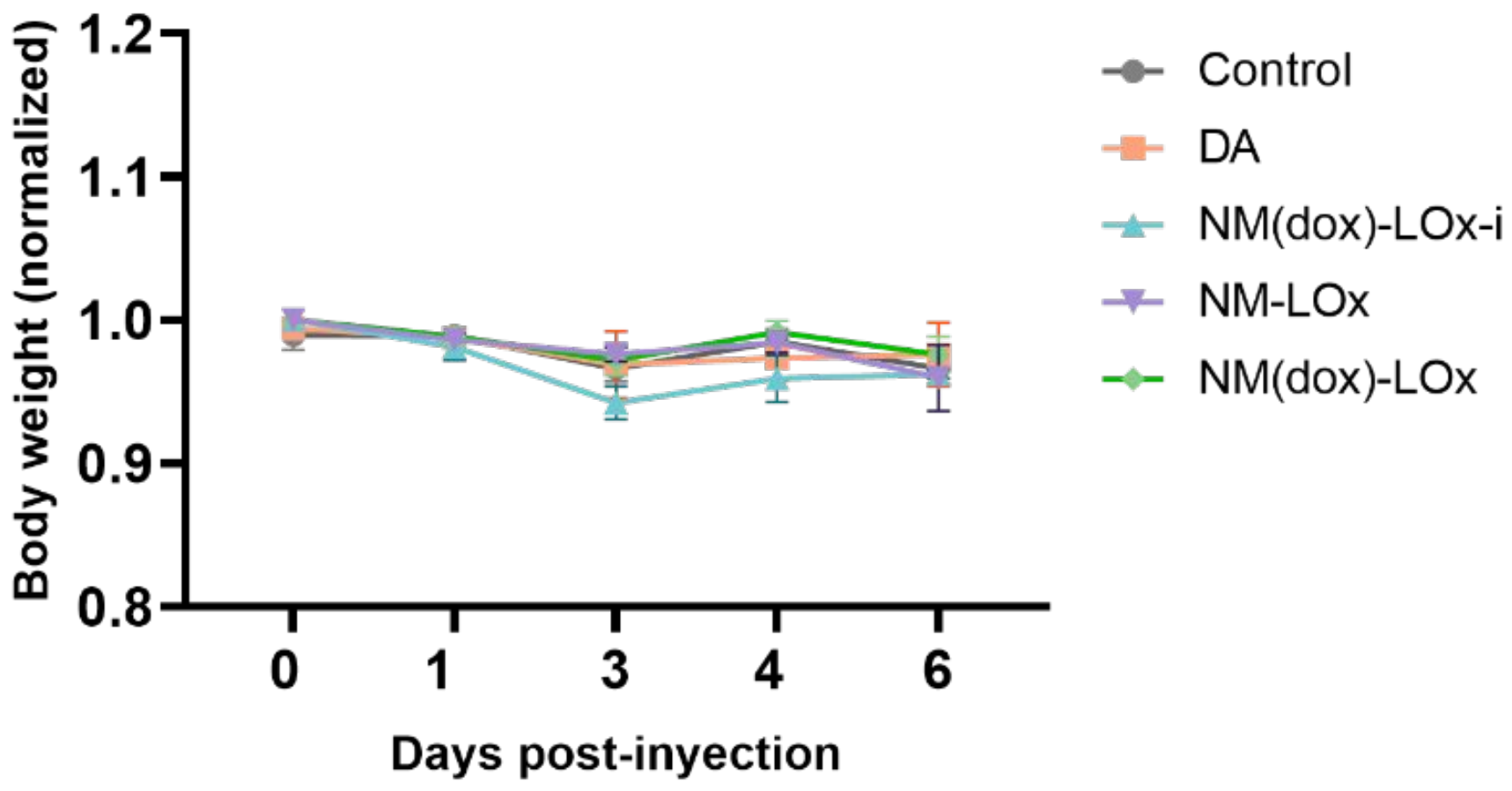

Figure S18. Body weight monitoring of tumor-bearing mice during treatment with NM(dox)-LOx, NM-LOx, NM(dox)-LOx-i, and DA. The average body weight was expressed as percentage relative to the initial value at day 0. Data are expressed as mean ± SEM, n = 4. Statistical significance was determined using two-way ANOVA. The differences were not statistically significant and, therefore, were not indicated. No significant body weight loss (>10 %) was observed in any treatment group, indicating good tolerability and systemic safety of the formulations.

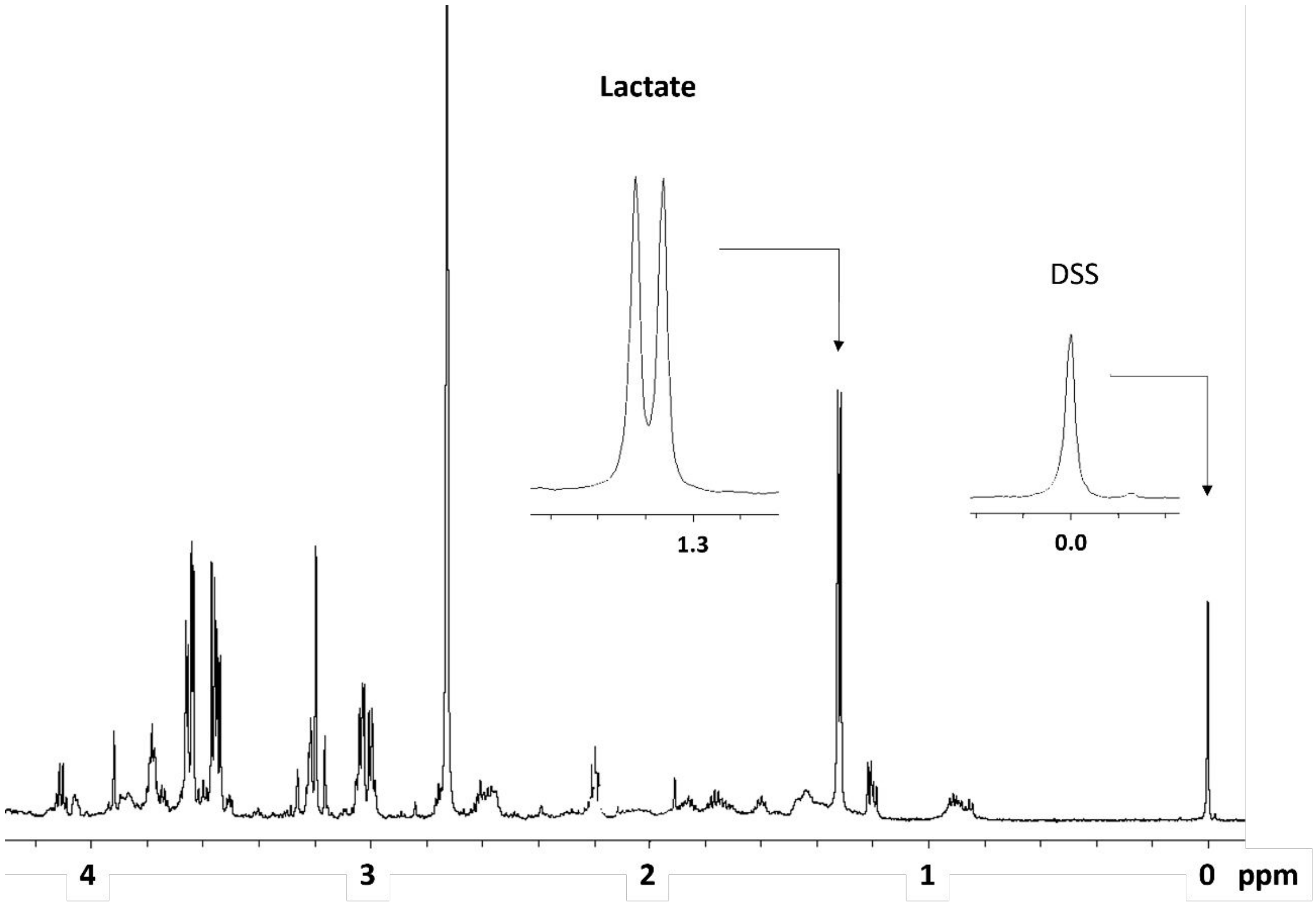

Figure S19. H-NMR noesy spectrum of a sample tissue extracted from SK-MEL-103 tumor xenografts in perchloric acid, resuspended in D_2O 0.1 mM in DSS-d_6. Lactate protons are observed in the spectrum (CH_3 doublet at 1.33 ppm and CH quadruplet at 4.11 ppm) enabling the assignment. The only non-deuterated protons of DSS-d_6 are observed in a singlet at 0.00 ppm, providing both chemical shift and concentration reference. Quantification was performed using Chenomx nmr suite V12.0, fitting Lactate signals regards DSS-d_6.

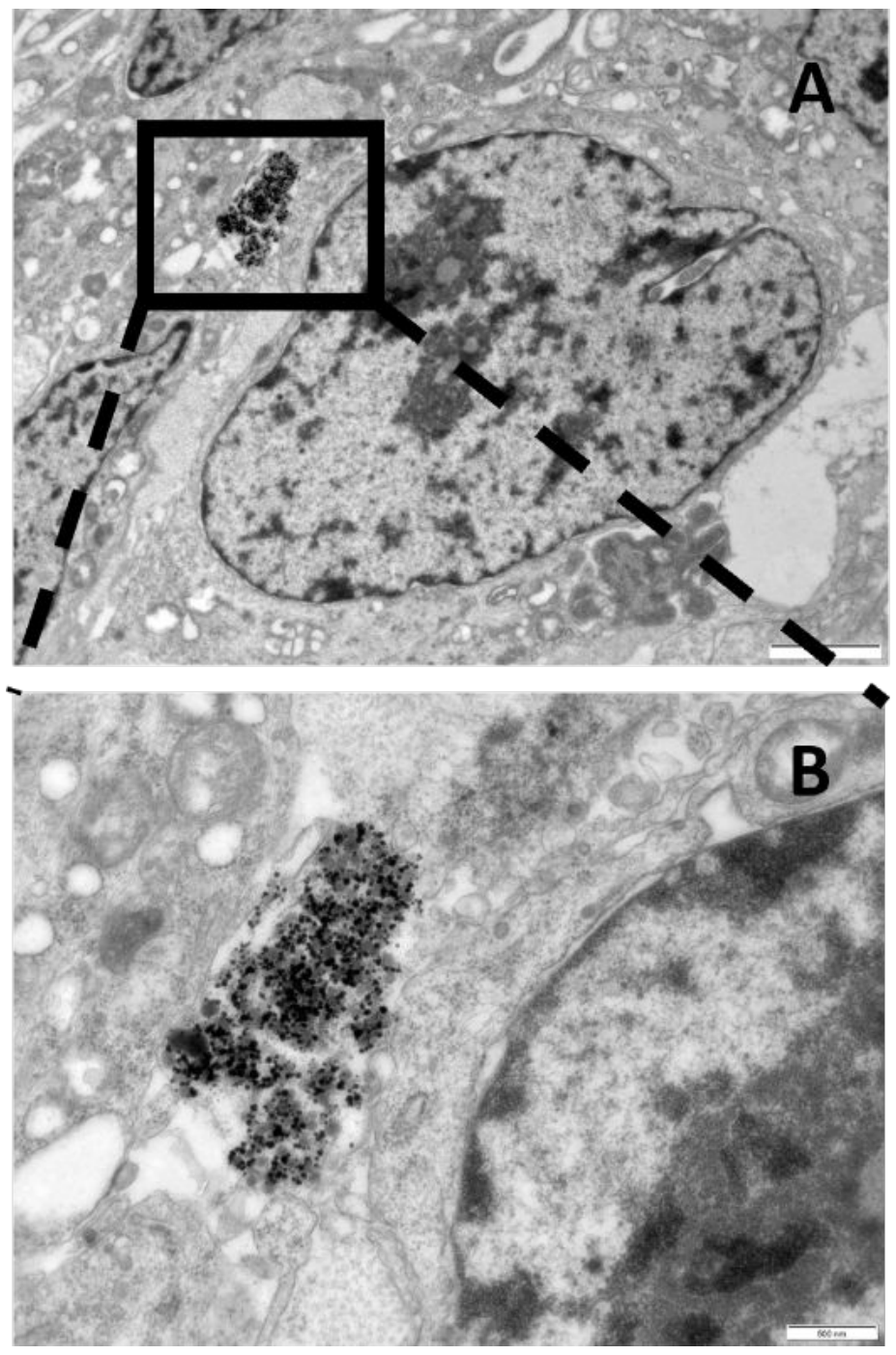

Figure S20. TEM images of SK-MEL-103 tumors treated with NM-LOx at 50 µg mL^{-1}, showing the presence of nanoparticles inside cells. Scale bars: (A) 2 µm and (B) 500 nm.

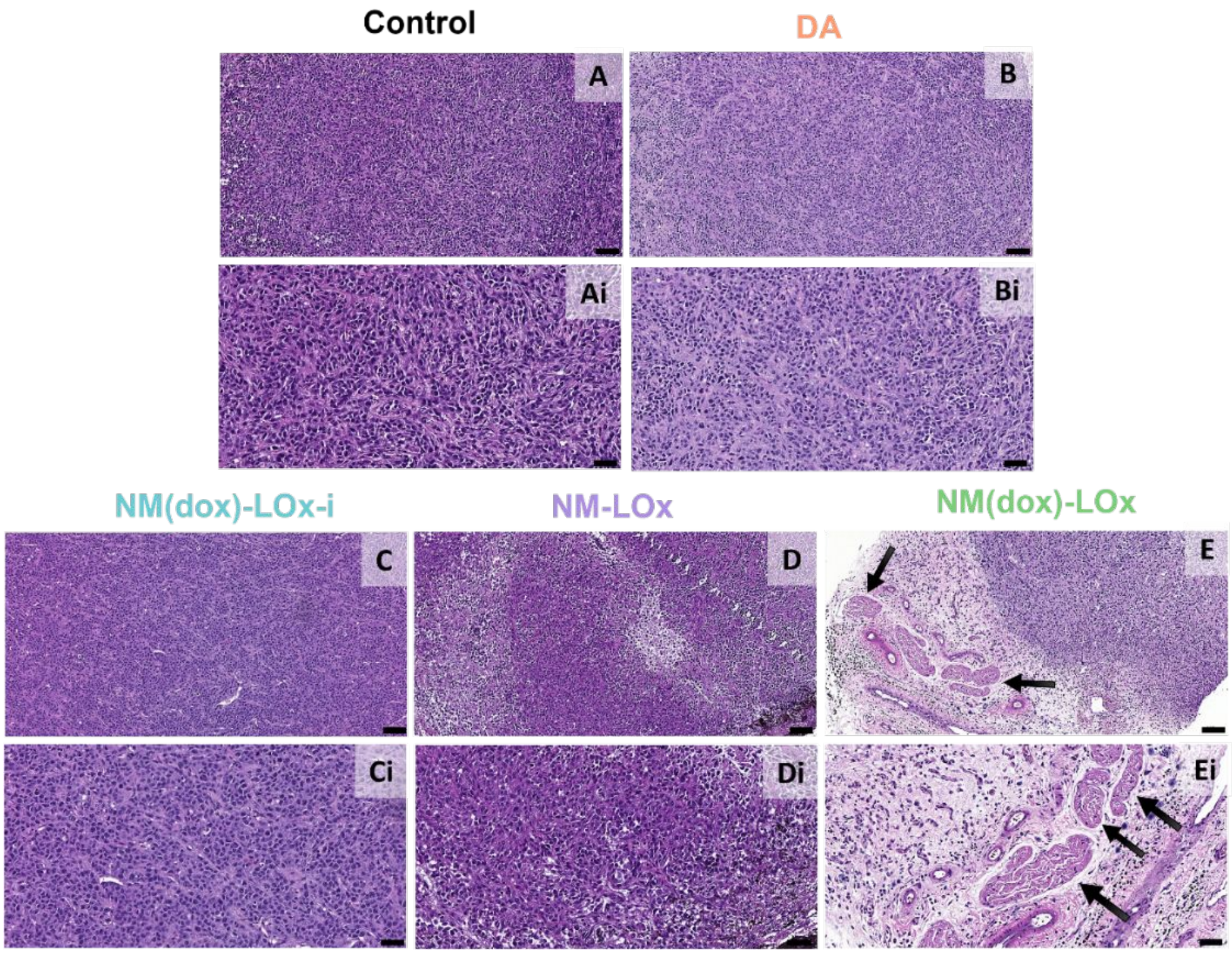

Figure S21. H&E-stained tumor sections from mice treated with: (A) vehicle, (B) DA, (C) NM(dox)-LOx-i, (D) NM-LOx and (E) NM(dox)-LOx. Necrotic regions are clearly observed in NM(dox)-LOx–treated tumors (marked with an arrow), with extensive loss of tissue architecture and eosinophilic debris. A-Ai are within the same anatomical region,

but Ai represents a smaller and more specific surface area. Scale bars: 100 µm (A-E) and 50 µm (Ai-Ei).

6. Nanomotors-induced immune system activation via lactate consumption in a syngeneic melanoma mouse model

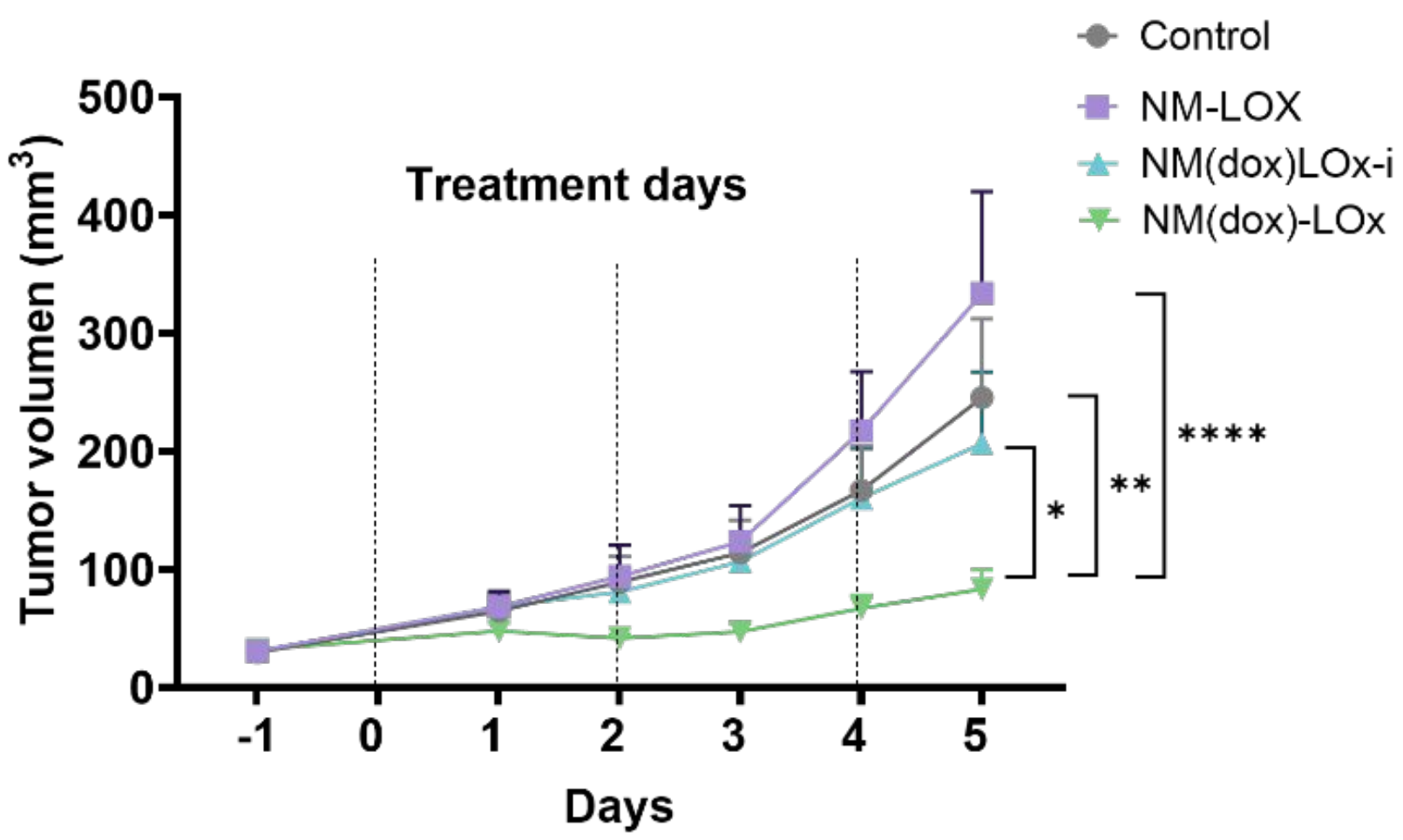

Figure S22. Monitoring of tumor volume after treatment with NM(dox)-LOx (green), NM-LOx (purple) or NM(dox)-LOx-i (blue), compared to control (grey). Data are expressed as mean ± SEM, n = 11. Statistical significance was determined by a two-way ANOVA, followed by Tukey's test (* $p < 0.05$, ** $p < 0.01$, ****$p < 0.0001$).

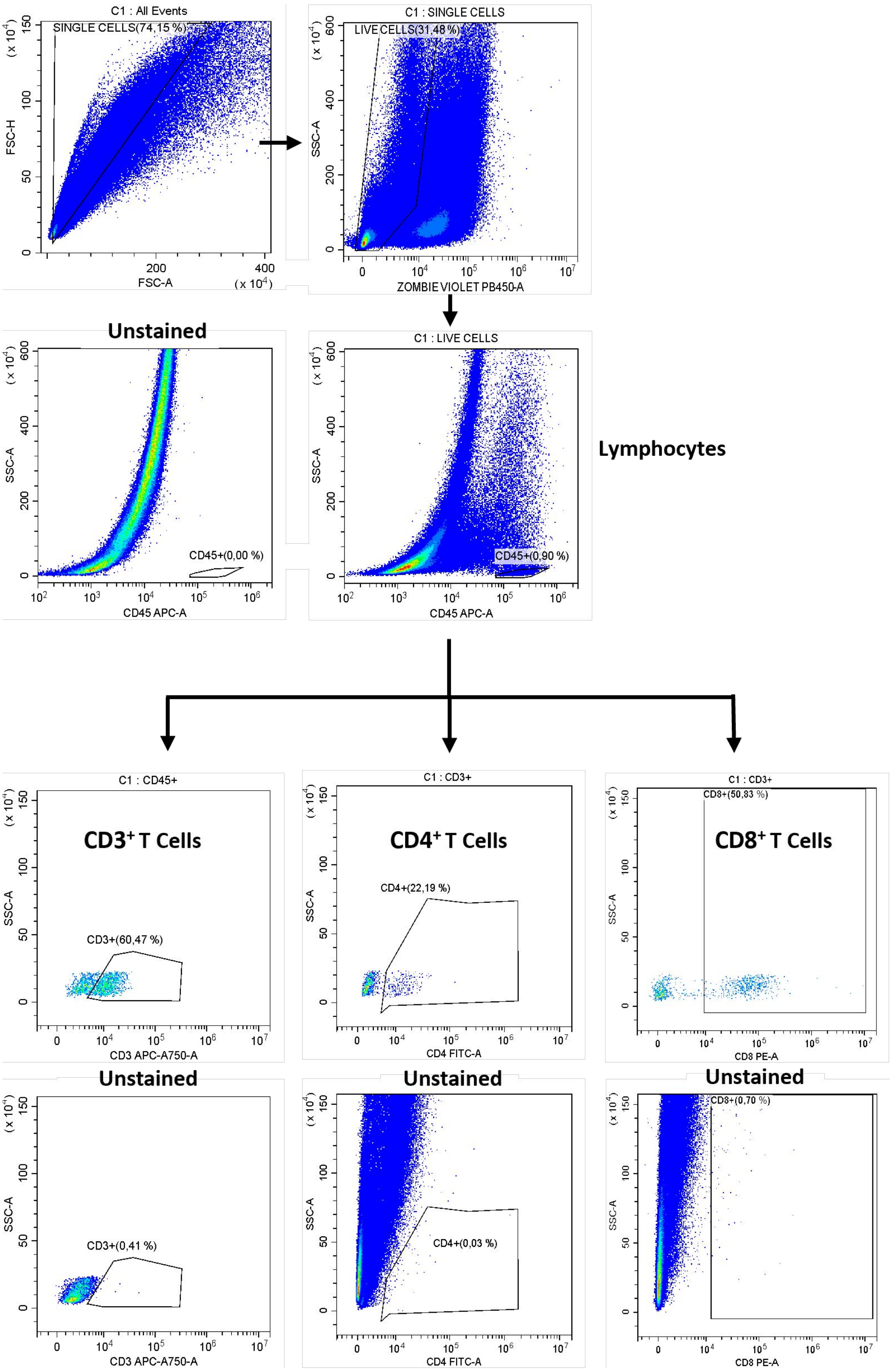

Figure S23. Extracellular staining of tumor-infiltrating lymphocyte. This is a representative gating strategy used for CD3$^+$, CD4$^+$, and CD8$^+$ T cells, n = 5 tumors per group. Unstained cells are shown as a control.

7. Nanomotor biocompatibility and biodistribution

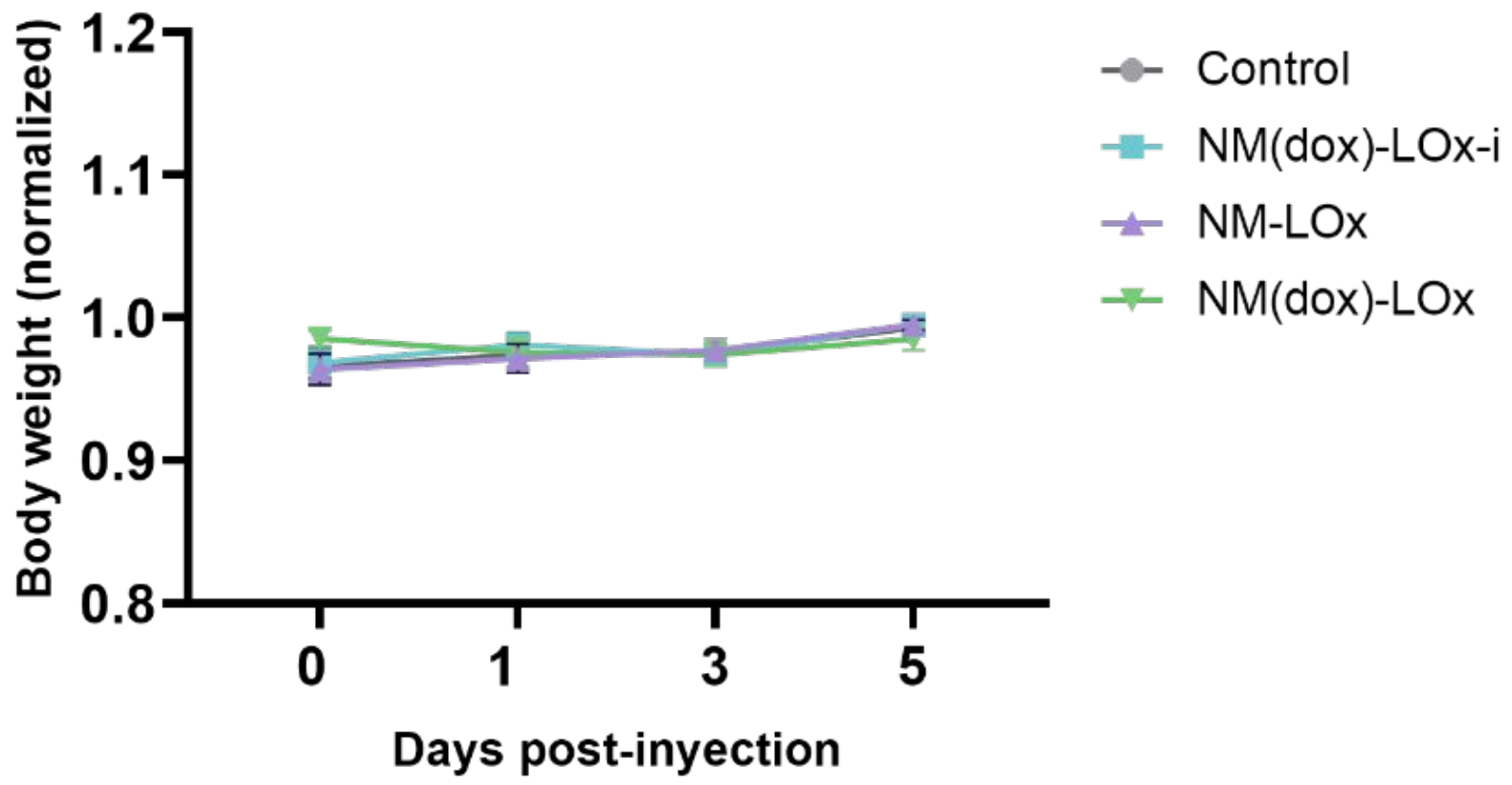

Figure S24. Body weight monitoring of melanoma mice model (C57BL/6 mouse bearing B16-F10 tumors) during treatment with NM(dox)-LOx, NM-LOx and NM(dox)-LOx-i. The average body weight was expressed as percentage relative to the initial value at day 0. Data are expressed as mean ± SEM (n = 11). Statistical significance was determined using two-way ANOVA. The differences were not statistically significant and, therefore, were not indicated.

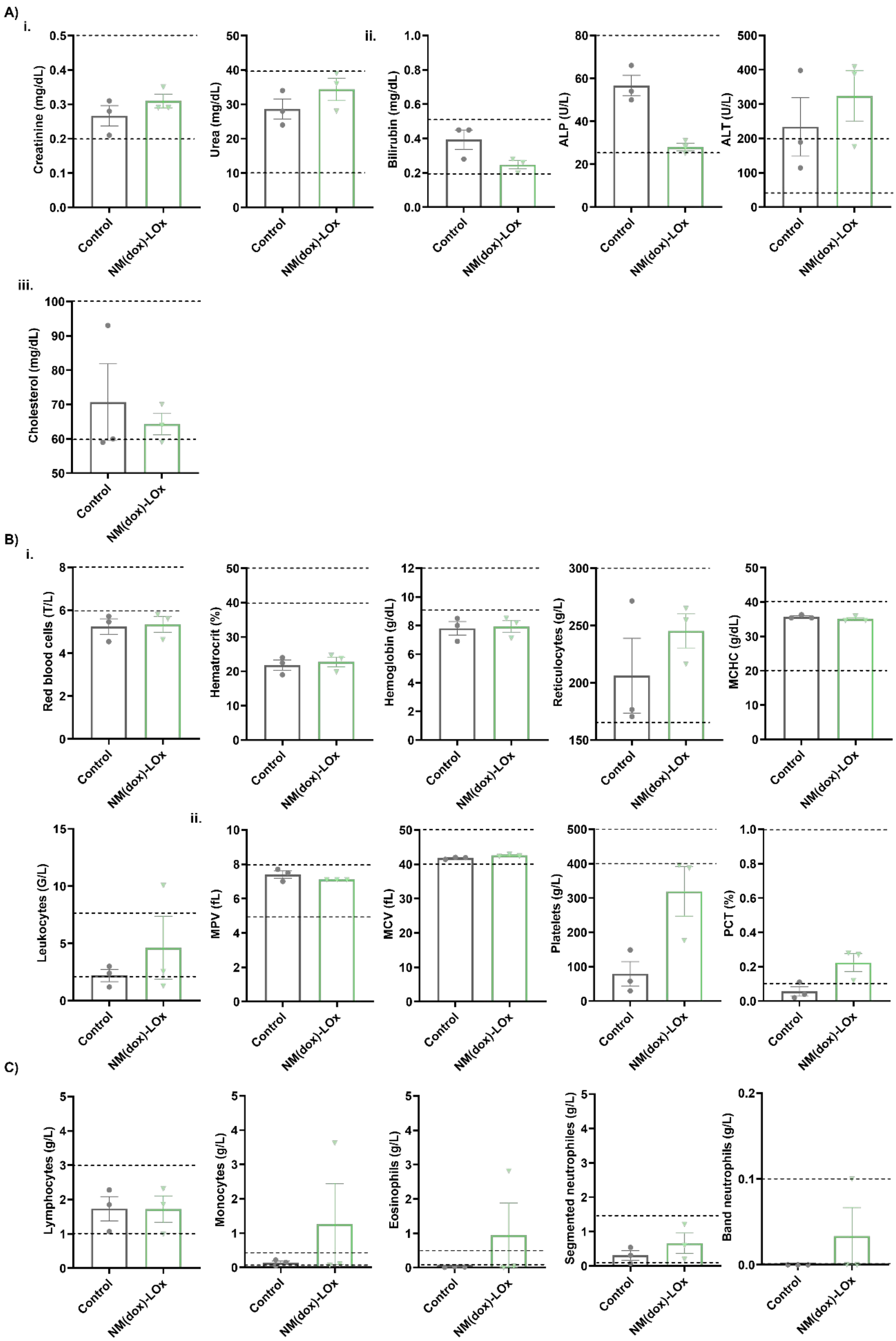

Figure S25. Hematological and biochemical analysis of mice blood samples collected at the study endpoint following systemic administration of NM(dox)-LOx (green) and

control (gray) to C57BL/6 mouse bearing B16-F10 tumors. (A) Biochemical markers of i) kidney and ii) liver function, including creatinine, blood urea nitrogen (Urea), bilirubin, alkaline phosphatase (ALP) and alanine aminotransferase (ALT) and iii) metabolic markers like cholesterol. (B) Hematological parameters including i) red blood cell count (RBC), hematocrit (HCT), hemoglobin (HGB), reticulocytes, mean corpuscular hemoglobin concentration (MCHC), leukocytes and ii) platelet recount that include mean platelet volume (MPV), mean corpuscular volume (MCV), platelet count (PLT), and plateletcrit (PCT). (C) White blood cell counts of lymphocytes, monocytes, eosinophiles, segmented neutrophils and band neutrophils. Data are expressed as mean ± SEM, n = 3. Statistical analysis was performed using one-way ANOVA with Tukey's post hoc test. The differences were not statistically significant and, therefore, were not indicated.

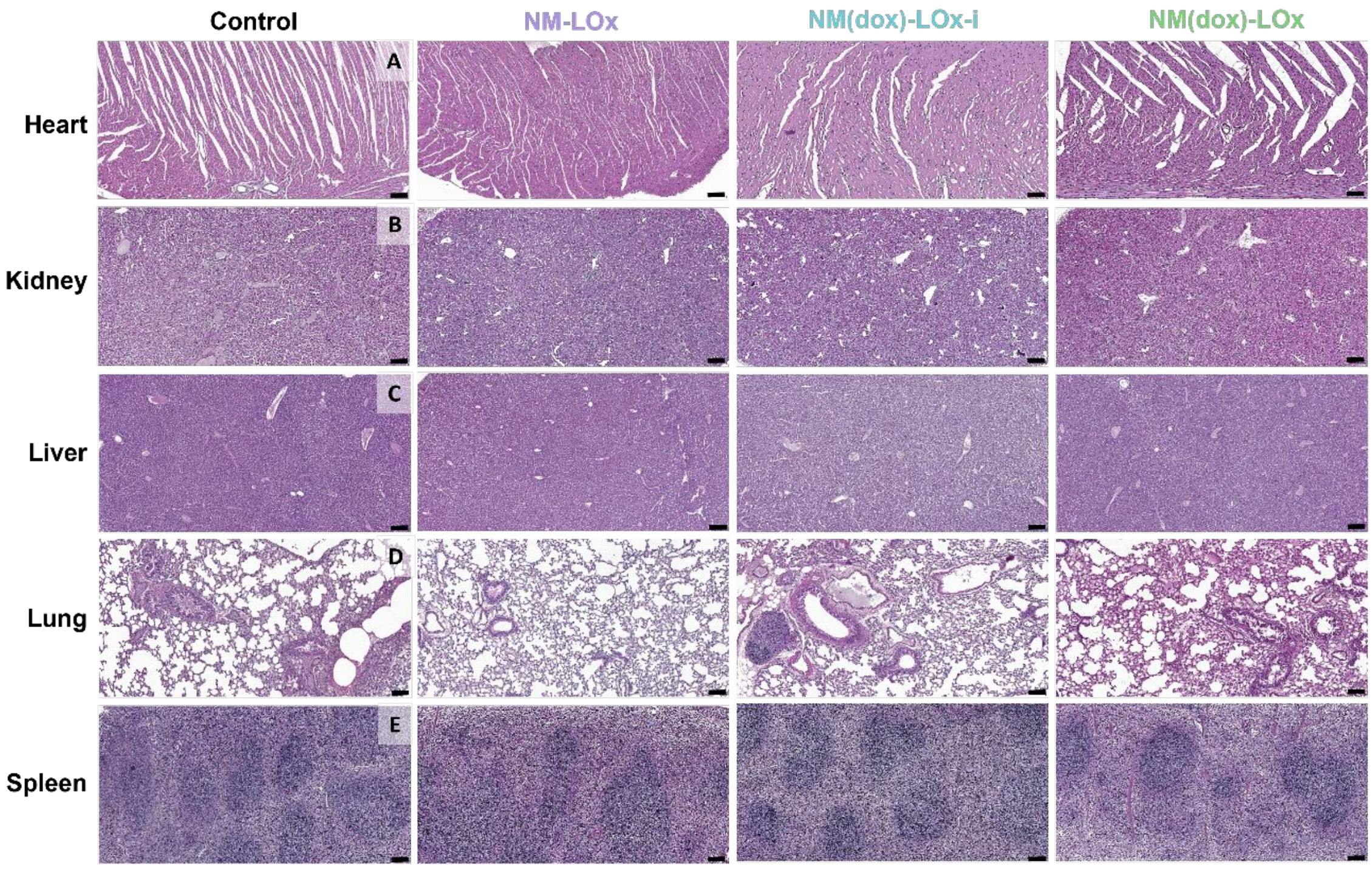

Figure S26. Representative H&E-stained sections of major organs: (A) heart, (B) kidney, (C) liver, (D) lung and (E) spleen from melanoma mice model (C57BL/6 mouse bearing B16-F10 tumors) treated with vehicle, NM-LOx, NM(dox)-LOx-i and NM(dox)-LOx. Scale bars: (A) Heart, 100 µm; (B) Kidney, 200 µm; (C) Liver, 200 µm; (D) Lung, 100 µm; (E) Spleen, 100 µm.

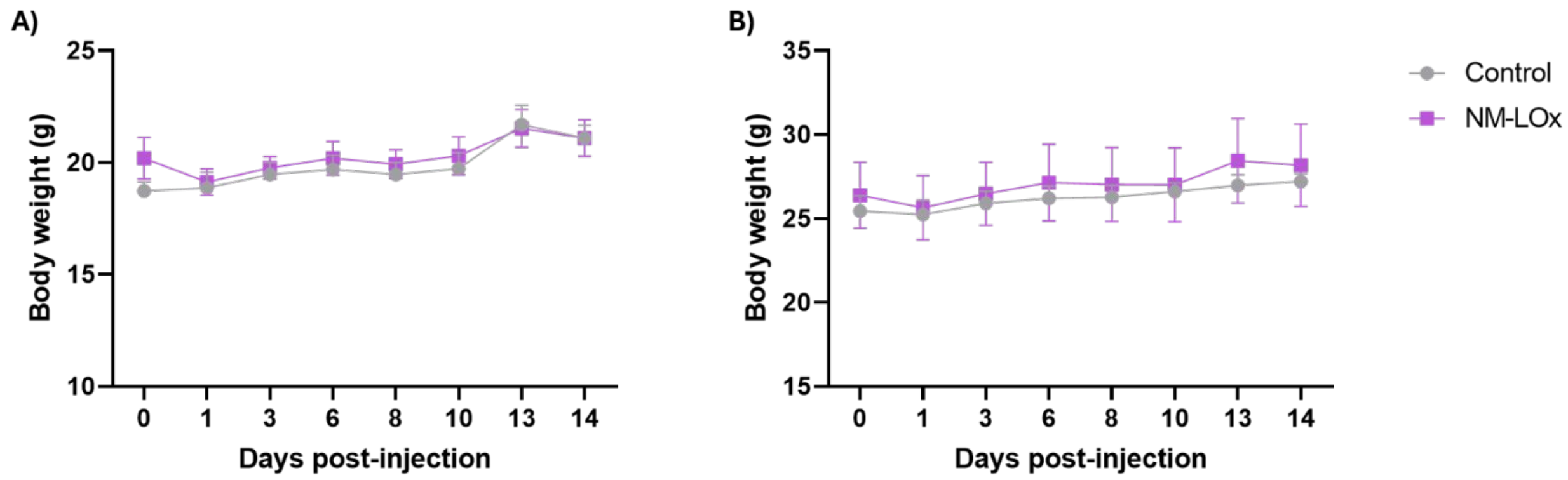

Figure S27. Body weight monitoring of healthy C57BL/6 mice during treatment with NM-LOx (purple) for 15 days compared to controls (gray). (A) Female, (B) male. Data are expressed as mean ± SEM (n = 6; 3 female and 3 male). Statistical significance was determined using two-way ANOVA. The differences were not statistically significant and, therefore, were not indicated.

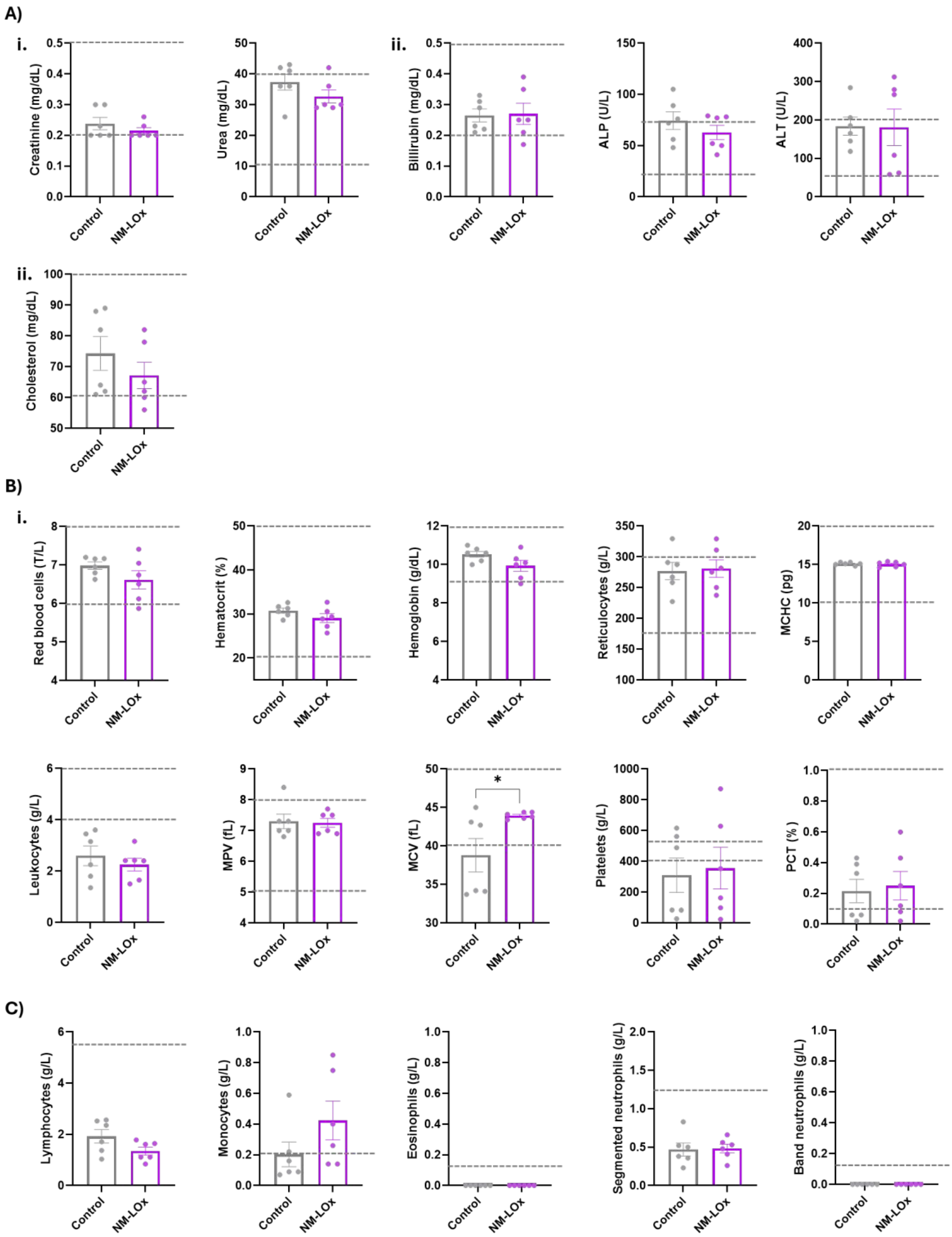

Figure S28. Hematological and biochemical analysis of mice blood samples collected 15 days after the systemic administration of NM-LOx (purple) and control (gray) to healthy C57BL/6 mice. (A) Biochemical markers of i) kidney and ii) liver function, including creatinine, urea, bilirubin, ALP and ALT and iii) metabolic markers like cholesterol. (B) Hematological parameters including i) RBC, HCT, HGB, reticulocytes, MCHC, leukocytes and ii) platelet recount that include MPV, MCV, PLT, and PCT. (C) White

blood cell counts of lymphocytes, monocytes, eosinophiles, segmented neutrophils and band neutrophils. Data are expressed as mean ± SEM, n = 6. Statistical analysis was performed using a T-Test (* $p < 0.05$). The differences that were not statistically significant were not indicated.

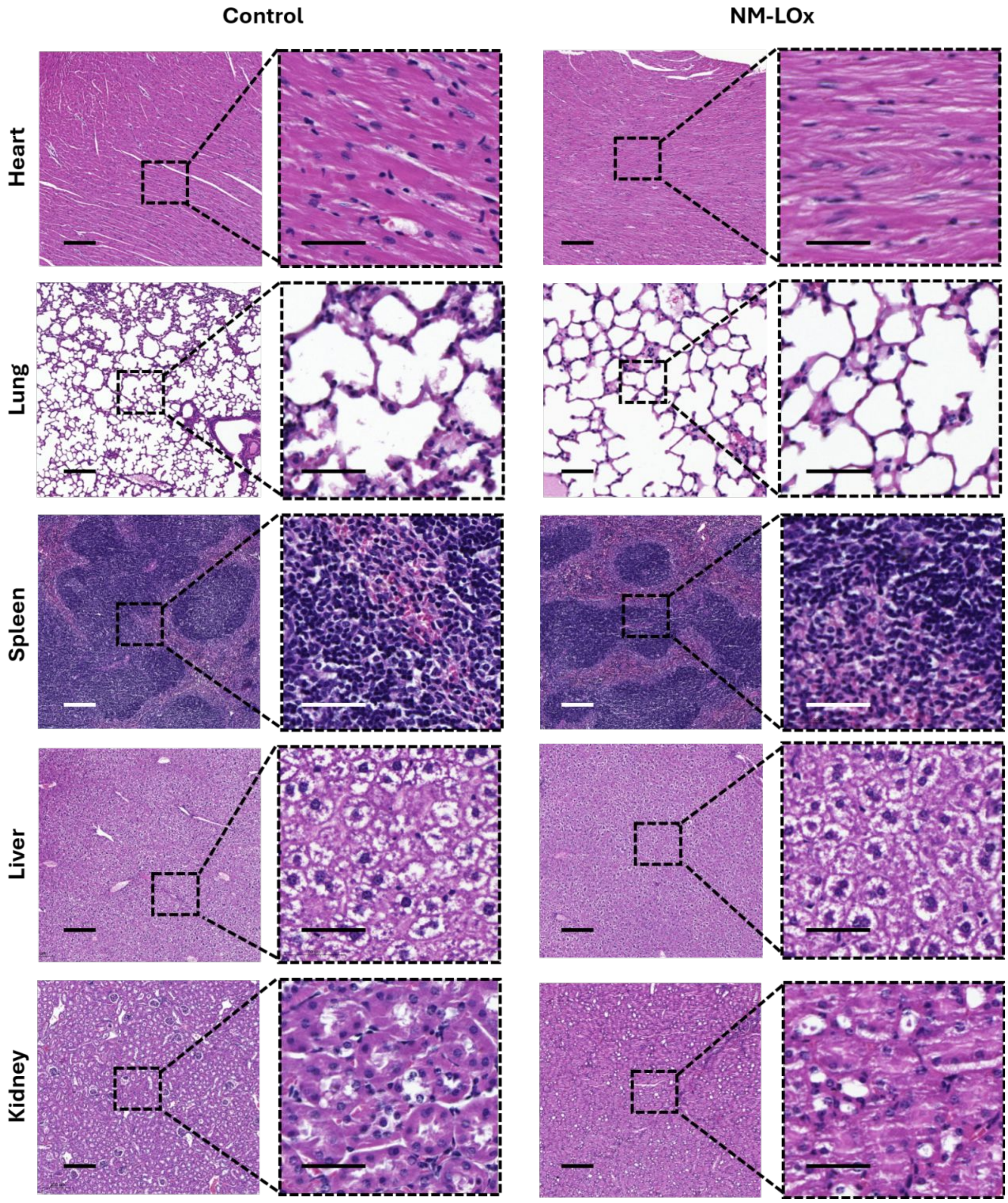

Figure S29. Representative H&E-stained sections of major organs: heart, lung, spleen, liver, and kidney from healthy C57BL/6 mice treated with NM-LOx for 15 days compared to controls. Scale bars = 200 µm and 50 µm (zoomed image).

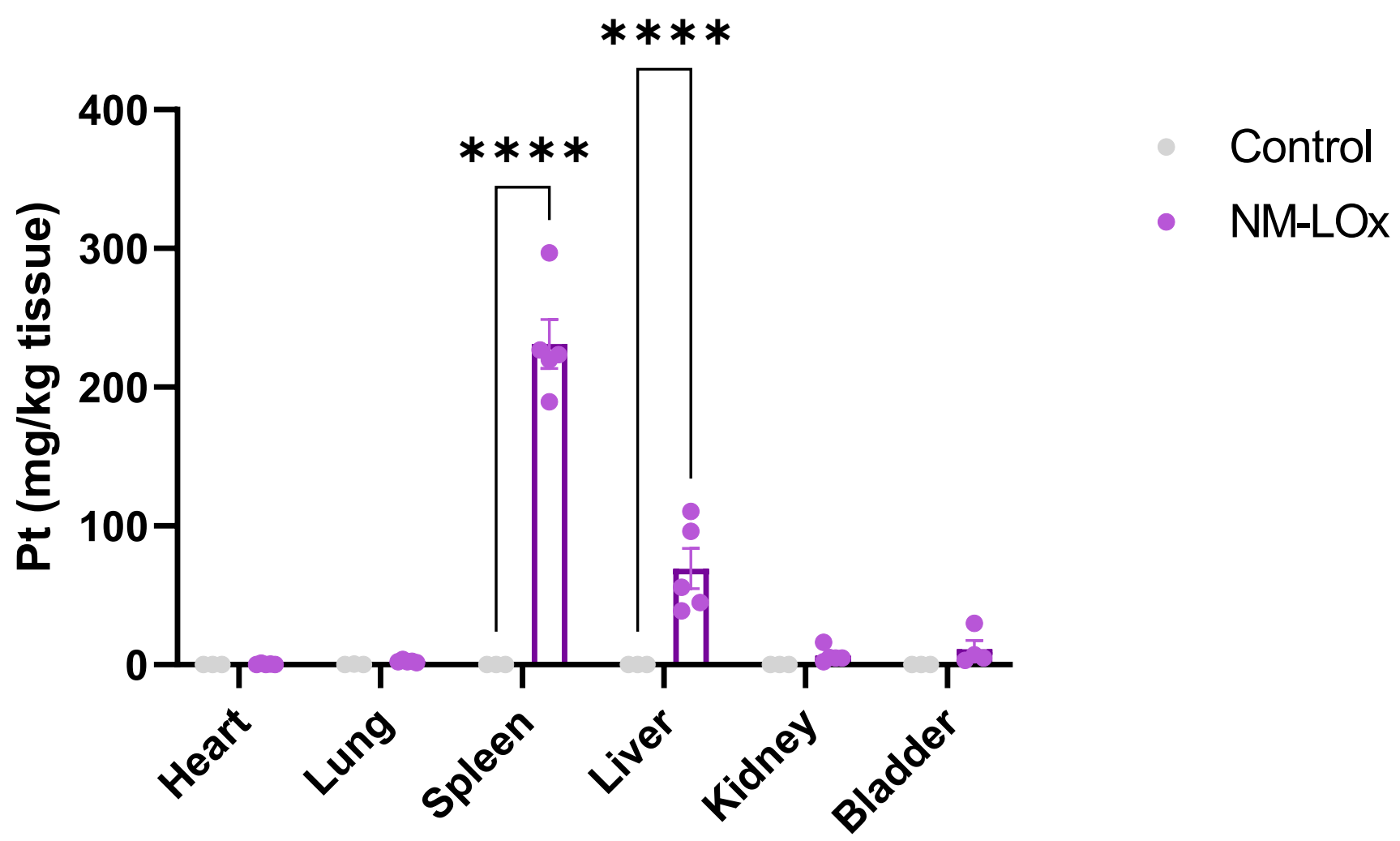

Figure S30. NM-LOx biodistribution in organs (heart, lung, spleen, liver, kidney and bladder) of healthy C57BL/6 mice 15 days after NM-LOx administration (60 mg kg^{-1}). Pt levels were analyzed by Inductively Coupled Plasma Mass Spectroscopy (ICP-MS). Data are expressed as mean ± SEM (n = 3 for controls and n = 5 for NM-LOx-treated mice) and represented as mg Pt per kg of tissue (Control: gray; NM-LOx: purple). Statistical significance was determined using two-way ANOVA (****$p < 0.000$).
